# From Molecular State to Clinical Outcomes After Spinal Cord Injury: A Scoping Review of Selected Fluid Biomarkers and Rehabilitation Relevance

**DOI:** 10.3390/ijms27219610

**Published:** 2026-10-27

**Authors:** Rocco Salvatore Calabrò, Angelo Quartarone, Andrea Calderone, Alessio Ardizzone, Loredana Raciti, Elisabetta Morini

**Affiliations:** 1 IRCCS Centro Neurolesi Bonino Pulejo, Via Palermo S.S. 113, Contrada Casazza, 98124 Messina, Italy; roccos.calabro@irccsme.it (R.S.C.); angelo.quartarone@irccsme.it (A.Q.); elisabetta.morini@irccsme.it (E.M.); 2 Departmental Faculty of Medicine, UniCamillusSaint Camillus International University of Health Sciences, 00131 Rome, Italy; alessio.ardizzone@unime.it; 3 Unipolar Spinal Unit, AOE Cannizzaro, 95126 Catania, Italy; loredana.raciti79@gmail.com

**Keywords:** spinal cord injury, fluid biomarkers, neurological prognosis, rehabilitation, scoping review

## Abstract

Human fluid biomarkers may complement neurological assessment after spinal cord injury (SCI), but their relevance to rehabilitation depends on the clinical relationship tested. This scoping review mapped selected neuroendocrine, inflammatory, neurotrophic and neural injury biomarkers in serum, plasma or cerebrospinal fluid. Five databases were searched on 8 October 2026; 17,317 records yielded 41 included reports. Findings were charted by phenotype, sampling window, assay, outcome and analytical denominator, with question-appropriate critical appraisal. Two reports randomized interventions, and 39 reports contributed nonrandomized biomarker analyses. Acute neural injury markers mainly addressed initial severity and later neurological status. Prognostic signals included neurofilament light, glial fibrillary acidic protein and tau, but all 10 prediction-model reports had high overall risk of bias; independent validation remained insufficiently established. Chronic endocrine, mobility and pain studies examined different constructs. Partial integration included biomarker–imaging models and trial-embedded associations; no identified report validated biomarker-guided rehabilitation dosing or a causal molecular–circuit–functional pathway. Reports with age-related eligibility uncertainty or combined neurotrauma were interpreted separately. Heterogeneous assays, overlapping cohorts, incomplete follow-up and incompletely quantified rehabilitation exposure limited inference. These data support focused prospective validation rather than routine biomarker-directed rehabilitation. Mandatory marker, matrix and outcome search terms constrain coverage; absence claims apply to the identified reports.

## 1. Introduction

Spinal cord injury (SCI) disrupts motor and sensory pathways while altering autonomic regulation, systemic physiology, and everyday independence [1]. Recovery depends on the extent and location of damage, the neural pathways that remain available, and the opportunities for rehabilitation. Patients with similar neurological classifications may nevertheless differ in pain, physiological stability, and functional progress. This variability creates a clinical need to understand whether fluid biomarkers can complement examination by describing biological states associated with subsequent outcomes or responses to rehabilitation.

Following the initiating injury, vascular compromise, cellular damage, and inflammatory responses contribute to secondary pathology [2]. Proteins released from neurons and glia may become detectable in cerebrospinal fluid (CSF) or blood, whereas endocrine and inflammatory measurements describe additional aspects of the organism’s response. These signals have different sources and timescales. An acute marker of tissue injury, a persistent inflammatory concentration, and a hormone response during rehabilitation cannot be interpreted as interchangeable indicators of recovery. Their clinical meaning depends on phenotype, biological matrix, sampling window, and outcome.

Neurological severity, recovery, and function must therefore be distinguished. The International Standards for Neurological Classification of Spinal Cord Injury (ISNCSCI) and American Spinal Injury Association Impairment Scale (AIS) characterize neurological impairment [3]. AIS conversion and changes in motor scores describe different aspects of neurological change, whereas the Spinal Cord Independence Measure (SCIM) assesses independence in daily activities [4]. Established prediction models demonstrate that baseline examination and age already provide substantial information about later walking ability [5]. Biomarkers should consequently be evaluated for the information they add to clinical assessment, with attention to the outcome predicted and the population studied.

Interpreting this information also requires distinguishing tissue preservation from circuit function. Magnetic resonance imaging (MRI) characterizes lesion morphology and structural preservation [6]. Motor-evoked potentials provide information about descending transmission, while functional performance reflects the interaction between remaining neural resources, task demands, and assistance. Improvements may arise through restitution, compensation, or adaptation, each with different implications for neuroplasticity [7]. A molecular measurement could help characterize the environment in which these processes occur, but its relationship with residual circuitry requires direct investigation. Neither reduced injury-marker concentrations nor increased neurotrophic-factor concentrations independently establish effective neural reorganization.

The hypothalamic–pituitary–adrenal (HPA) axis provides a rationale for examining stress biology within this framework. Its regulation of glucocorticoid secretion links physiological and psychological challenges to endocrine responses [8]. After SCI, pain, illness, medication, disrupted autonomic regulation and the demands of rehabilitation may influence how these responses are interpreted. Basal cortisol and adrenocorticotropic hormone (ACTH)-stimulated cortisol address different aspects of adrenal physiology. Neither is a direct substitute for perceived stress, and an isolated concentration cannot establish persistent HPA dysregulation. These distinctions are essential when endocrine measurements are related to inflammatory activity, neurological status or recovery.

Experimental evidence provides a biological basis for considering neuroplasticity in this context. In mice, circadian glucocorticoid oscillations influence the formation and stabilization of learning-associated cortical synapses, whereas prolonged excessive exposure can disrupt these processes [9]. Such findings motivate investigation of stress-related endocrine activity alongside rehabilitation, while remaining distinct from evidence of neurological recovery in people with SCI. Likewise, brain-derived neurotrophic factor (BDNF) is relevant to plasticity-related biology, but a circulating concentration does not demonstrate where or how neural adaptation occurs. Establishing a clinical link requires an independently assessed structural or physiological plasticity outcome and appropriately timed measurements.

Rehabilitation exposure is another component of the same question. Repeated practice can engage remaining neural pathways, but prescribed treatment does not necessarily capture delivered intensity, repetitions, assistance, or adherence. Biological measurements obtained after one exercise bout address different questions from measurements during a sustained program. Technological interventions may improve performance while active, whereas persistent improvement after assistance or stimulation has ceased represents a separate endpoint [10]. Biomarker associations should therefore be interpreted alongside actual treatment exposure and assessment conditions, with randomized treatment contrasts distinguished from observational relationships within trials.

A clinically useful synthesis must establish what each biomarker measures, which outcome it informs, and whether different biological domains have been examined jointly. This is necessary to distinguish potentially informative integration from co-measurement and to identify the evidence needed before biomarkers can guide rehabilitation.

This scoping review primarily aims to map human evidence relating selected neuroendocrine, inflammatory, neurotrophic, and neural injury biomarkers in serum, plasma, or CSF to injury severity, neuropathic pain, neurological change, and functional outcomes. Traumatic and non-traumatic SCI are considered, with compressive myelopathy and perioperative cord injury treated as distinct clinical contexts. The secondary aim is to identify relationships actually tested between biological domains and their integration with imaging, residual neurological function, and rehabilitation exposure. Within this aim, an exploratory question asks whether stress-related HPA measures have been linked to independently assessed neuroplasticity after SCI. By separating biological plausibility, concurrent association, prognosis, and treatment-related findings, the review seeks to clarify rehabilitation relevance and identify priorities for prospective validation.

## 2. Materials and Methods

### 2.1. Review Design, Registration and Reporting

A scoping review was undertaken to characterize selected fluid biomarkers, clinical relationships, and analytical designs after SCI, rather than estimate a common treatment effect [11,12]. Reporting follows the Preferred Reporting Items for Systematic Reviews and Meta-Analyses extension for Scoping Reviews (PRISMA-ScR) [13]. The completed checklist is provided as a separate document. PRISMA 2020 informs the flow diagram [14], and PRISMA-S informs the documented search reporting [15]. The Open Science Framework (OSF) was used for protocol registration [16].

The protocol was registered on 4 July 2026, before the initial search on 6 July 2026, under DOI 10.17605/OSF.IO/M3JDT. Amendments were decided and registered on OSF on 7 October 2026, before the updated search and screening began on 8 October 2026. They followed an earlier evidence map and examination of the literature included in that version. The amendments refocused a broad stress, autonomic, imaging, neurophysiological and intervention scope on selected biomarkers in serum, plasma or CSF linked to specified clinical relationships. They also specified an adult-focused population, English-language eligibility, a target-based search with explicit specimen and clinical-concept blocks, and more detailed coding of timing, relationships, analytical denominators and rehabilitation exposure. Structured tool-specific appraisal supplemented the descriptive methodological charting in the original protocol. Table S1 summarizes these post-registration changes and their chronology.

### 2.2. Review Question, Analytical Framework and PICOS(T)

The review was guided by three questions. Which clinical relationships have been examined for eligible fluid biomarkers after SCI, and how do injury phenotype, sampling time, and endpoint definition affect their interpretation? When biomarkers are assessed alongside another biological domain, imaging, residual neurological function, or rehabilitation exposure, which relationships are explicitly tested? Do eligible human studies connect HPA-axis measurements with independently assessed indicators of neuroplasticity, and is psychological stress measured directly or inferred from endocrine physiology?

Population, Intervention or exposure, Comparator, Outcome, Study design and Timing (PICOS(T)) structure eligibility and data charting (Table S2). In observational analyses, exposure denotes biomarker concentration, longitudinal trajectory, or stimulated response. Comparisons may involve clinical groups, continuous associations, or within-person change. Neither a separate control arm nor longitudinal follow-up is mandatory. Comparisons with healthy participants alone are insufficient without an eligible clinical relationship within SCI. Concurrent severity, subsequent neurological status, and recovery from baseline are treated as different outcomes. HPA activity is distinguished from perceived stress, and affective pain components do not substitute for dedicated assessments of mood, cognition, or learning.

Neuroendocrine charting distinguishes basal cortisol, ACTH-stimulated responses, adrenal androgens, and thyroid measures. Challenge dose, sampling time, acute illness, and medication are retained because these variables may affect interpretation independently of perceived psychological stress.

### 2.3. Eligibility Criteria and Biomarker Domains

#### 2.3.1. Inclusion Criteria

The review focuses on adults aged 18 years or older with traumatic or non-traumatic spinal cord injury. Compressive or degenerative myelopathy, acquired spinal cord tethering and perioperative cord injury are also eligible, provided that spinal cord involvement is identifiable. These conditions are characterized separately because their mechanisms, clinical trajectories and sampling contexts differ from those of acute traumatic injury. Acute, subacute and chronic presentations are eligible.

Eligible publications are peer-reviewed original research reports published in English. Study designs include randomized and non-randomized intervention studies, prospective or retrospective cohorts, case-control studies, cross-sectional analyses, physiological experiments and case series. Reports combining human and preclinical investigations contribute only separately extractable human findings.

Biomarker eligibility requires measurement of at least one target from four specified domains. The neuroendocrine domain includes cortisol, adrenocorticotropic hormone (ACTH), dehydroepiandrosterone (DHEA), dehydroepiandrosterone sulphate (DHEAS) and thyroid hormones. Inflammatory targets comprise C-reactive protein (CRP), interleukin (IL)-6 and IL-8. The neurotrophic domain includes brain-derived neurotrophic factor (BDNF), nerve growth factor (NGF) and glial cell line-derived neurotrophic factor (GDNF). Neural and glial injury markers comprise glial fibrillary acidic protein (GFAP), neurofilament light and heavy chains, phosphorylated neurofilament heavy chain, S100 calcium-binding protein B, neuron-specific enolase, tau and contactin-1.

The rationale for the focused target list is to map complementary biological constructs within a defined fluid-biomarker scope. Adrenal and thyroid measures characterize endocrine physiology, CRP and IL-6/IL-8 provide selected acute-phase and cytokine measures, BDNF/NGF/GDNF represent neurotrophic signalling, and the neural proteins reflect distinct cellular components of injury. Their selection does not imply superiority or clinical validation relative to unlisted markers. The original protocol named several of these analytes and allowed broader biological and non-molecular measures. The exact current list and mandatory serum/plasma/CSF criterion were specified in the October amendment, before the updated screening but after the earlier literature review. The map is therefore selective within each domain.

The target must be assayed in serum, plasma or CSF. Assays of CSF-derived exosomal fractions are eligible when the specimen and fraction are explicitly identified. Additional analytes can be charted when measured within a panel containing an eligible target, but their presence does not independently establish eligibility or demonstrate that every panel constituent has a clinical association. Targets assayed below the detection limit are distinguished from biomarkers yielding quantifiable concentrations.

A qualifying report must examine a clinical relationship involving injury severity, neuropathic pain, neurological status or change, task performance or everyday independence. Eligible analyses include severity-group contrasts, biomarker–pain associations, longitudinal biomarker–outcome relationships and intervention-related comparisons. Repeated sampling qualifies when linked to an eligible clinical construct. Eligibility does not depend on the direction or statistical significance of the finding.

Five reports were retained as explicit eligibility exceptions. Ahadi et al. [17] contributes serum neural injury marker analyses against initial SCI severity. Its reported 16–64-year age range covers all 35 trauma participants, including nine controls, without a separate range for the 26 participants with SCI. Itodo et al. [18] and Seijas et al. [19] contribute serum adrenal androgen analyses from SwiSCI. Recruitment permitted ages above 16 years in Itodo and ages 16 years or older in Seijas, without separately reported adult-only estimates. Their findings are therefore retained as documented age-related exceptions and cannot establish adult-only effects. Itodo examines injury completeness and hormone trajectories, whereas Seijas examines functioning during initial rehabilitation. Possible cohort overlap is documented in Table S6c. Pu et al. [20] contributes serum neuron-specific enolase findings in patients with SCI and concomitant traumatic brain injury, although its global neurotrauma outcomes cannot isolate spinal recovery. Ding et al. [21] contribute circulating CRP associations with neurological severity and later AIS status despite an unresolved serum/plasma designation, which limits matrix-specific interpretation. These exceptions and their applicability limits are documented in Tables S1 and S3.

These were case-by-case retention decisions made after examination of the individual reports, rather than applications of a prespecified exception rule. Their contribution was retained to map the reported biomarker–clinical relationships, while population and specimen limitations remained explicit. Dependence of the synthesis on these decisions was examined separately, as described in Section 2.8.

#### 2.3.2. Exclusion Criteria

Reviews, editorials, commentaries, protocols, registry-only records, conference abstracts without an eligible full report, and single-case reports are excluded. Exclusively animal, in vitro, or tissue-based investigations are outside the clinical scope. Paediatric-only populations and reports without identifiable spinal cord involvement are ineligible, subject to the documented population exceptions above.

Human studies are excluded when they do not assay a specified target in an eligible fluid matrix or do not examine an eligible clinical relationship. Comparisons restricted to spinal cord injury versus healthy controls are insufficient without a qualifying analysis within the spinal cord injury population. Reports describing only biochemical concentrations or serial biomarker trajectories, without linkage to clinical status or outcomes, are likewise ineligible.

Measurement across multiple biological domains is not required. Absence of a HPA measurement, imaging assessment, or rehabilitation intervention is not an exclusion criterion. Independently assessed neural reorganization or adaptation is required to classify a finding as evidence concerning neuroplasticity. Biomarker changes or functional gains alone do not meet that definition, although the report may remain eligible through another clinical relationship.

### 2.4. Information Sources and Search Strategies

Searches were conducted on 8 October 2026 in PubMed, Web of Science, Embase, Cochrane Library and Scopus. These sources were selected to address the clinical and biological scope of the review through complementary coverage. PubMed provides access to biomedical and life sciences literature, including MEDLINE-indexed publications. Embase offers additional biomedical and pharmacological coverage relevant to biomarker research. Web of Science and Scopus extend retrieval across disciplines relevant to SCI, including neuroscience, rehabilitation and molecular biology. The Cochrane Library was included to identify controlled-trial reports through the Cochrane Central Register of Controlled Trials (CENTRAL). Eligibility was determined from the content of each report rather than its database classification. Search results were exported for study selection.

R.S.C. and A.C. conducted the searches independently. Database-specific formulations and their implementation were compared and discussed to establish consensus. Disagreements concerning terminology, query adaptation or search procedures were resolved through discussion, with A.Q. acting as the third reviewer when agreement could not be reached.

The search strategy comprised four mandatory conceptual blocks representing spinal cord injury, eligible biomarker targets, biological fluid matrices and relevant clinical constructs. Alternative terms within each block were combined using OR, and the four blocks were combined using AND. The biomarker block allowed retrieval through any specified biological domain. Compared with the earlier supplementary strategy, the updated expressions removed the human-participant term block and replaced the broader autonomic, imaging, stimulation and drug terminology with selected molecular targets and an explicit matrix block. Clinical-concept vocabulary and searchable fields were also revised. Because these changes combined extensions and restrictions, the updated search was a reformulation of scope rather than a demonstrated increase in retrieval sensitivity. Database-specific adaptations retained the four-block structure while accommodating interface syntax and vocabulary.

All-fields searches were used, with syntax and field scope adapted to each database interface. No publication-date, language, age, human-only, or study-design filters were applied at retrieval. The English-language restriction was applied during eligibility assessment. Scopus was searched through its all-fields query builder, rather than TITLE–ABS–KEY. Its all-fields scope includes reference metadata as well as titles, abstracts, keywords and other indexed fields, and double quotation marks specify approximate phrases. PubMed used an untagged expression with automatic term mapping for eligible unquoted terms and phrase-index handling for quoted terms. Consequently, identical conceptual blocks do not imply equivalent searchable content or term processing. Tables S4a and S4b specify the fields, settings and retrieval counts, and Table S5 reproduces the executed expressions. The mandatory biomarker, matrix and clinical-concept blocks can miss otherwise eligible reports when relevant details are absent from searchable metadata. Broader field coverage does not remove this limitation.

### 2.5. Study Selection and Data Charting

Screening of the updated search began on 8 October 2026. Study selection comprised title and abstract screening followed by full-text assessment against the amended eligibility criteria. R.S.C. and A.C. performed both stages independently. Disagreements were resolved through discussion and consensus, with A.Q. acting as the third reviewer when agreement could not be reached. The selection process distinguished bibliographic records from publication reports. Reports that could not be retrieved were classified separately from those excluded after full-text assessment, and exclusion reasons were documented according to the eligibility criteria.

R.S.C. and A.C. independently charted data at the report level using a structured framework aligned with the review questions. Clinical and methodological fields included setting, participant characteristics, injury aetiology and level, neurological classification, study design, comparators, and recruitment details. Biomarker fields captured the analytes assessed, specimen matrix, assay platform, sampling schedule, and specimen handling. Detection limits, storage conditions, haemolysis, measurement blinding, and procedures for handling concentrations below the detection limit were recorded when reported. These characteristics are organized in Tables S6a and S6b. Recruitment, biological sampling, intervention exposure, and clinical assessment were recorded separately to preserve their temporal relationships.

For each analysis, charting distinguished enrolled, randomized, assayed, longitudinally paired, and outcome-assessed participants. Biomarkers and clinical denominators were retained separately, including differences arising from specimen availability, missing measurements, or incomplete follow-up. Repeated specimens, visits, and observations were distinguished from individual participants.

Missing information was coded according to its meaning. NR indicates information not reported, NM identifies a construct not measured, and NA denotes a field that is not applicable. Potential cohort overlap was examined using recruitment periods, study settings, and participant descriptions and classified as established or possible in Table S6c. Reports drawing on overlapping cohorts were retained as separate publications but were not treated as independent participant samples or independent replications of the same finding.

### 2.6. Temporal Classification and Relationship Coding

Acute SCI is defined operationally as ≤14 days after injury, subacute SCI as >14 days to ≤6 months, and chronic SCI as >6 months. These review-specific boundaries provide consistent charting rather than a universal biological staging system. Recruitment, biomarker sampling, intervention delivery, and outcome assessment are recorded separately. Exact intervals take precedence over author labels. Windows crossing boundaries are classified as mixed, while insufficiently specified intervals remain unclassified. For perioperative injury or acute neurological deterioration, timing is anchored to the new event while the preceding disease course remains separately classified. Biomarker-domain definitions are provided in Table S7, and exact sampling and outcome windows are organized in Table S8a.

Relationships are coded as co-measurement, concurrent association, longitudinal prognosis, within-person intervention change, randomized treatment contrast, formal mediation, or treatment-by-biomarker interaction (Table S8b). Co-measurement alone does not establish an examined association. Prognostic coding requires biomarker assessment before the relevant outcome. Association with a subsequent neurological grade does not establish prediction of grade conversion or motor-score change. Mediation and interaction are coded only when corresponding analyses are reported.

Imaging and physiological measures retain their specific meanings. MRI morphology represents structural information, sensory zones of partial preservation represent clinically preserved sensation, motor-evoked potentials and D-waves address descending transmission, reflex measures address segmental physiology, and electromyography characterizes muscle activation. These measurements contribute to integration only when linked to findings within otherwise eligible reports. Fluid-factor change or functional improvement alone are insufficient evidence of neuroplasticity.

Biological-domain counts are non-exclusive because reports may assess several targets. Domain membership indicates assessment rather than a positive association, a validated biomarker, or an independent replication of a clinical finding. Table S9 records source-reported estimates, uncertainty, null findings, and analytical denominators; these data were charted without recalculation or imputation.

### 2.7. Result-Specific Critical Appraisal

Critical appraisal was used to qualify interpretation and did not determine eligibility. Formal instruments were applied to explicitly defined randomized results, prognostic-factor relationships, prediction models or descriptive analyses. For report-level allocation summaries, the principal unit was a developed multivariable model intended to predict an individual outcome when present, otherwise an early biomarker–later outcome association, or the concurrent, descriptive or intervention question represented in Table S9. This allocation reflects the relationship emphasized in the review rather than a global judgment of the report; it was not based on statistical significance or effect magnitude. Contextual criteria are specified in Table S10a. The revised Cochrane risk-of-bias tool for randomized trials (RoB 2) was applied to treatment-assignment contrasts [22]. Its five domains address randomization, deviations from intended interventions, missing outcome data, outcome measurement, and selection of the reported result. Clinical and laboratory outcomes were appraised separately (Table S10b). The crossover-specific version was used for the aquatic-therapy sequence comparison, including its additional domain for period and carryover effects (Table S10b2) [23]. Observational biomarker–outcome associations within randomized reports were distinguished from assigned-intervention effects.

The allocation of nonrandomized analyses to appraisal instruments is documented in Table S10c, with the domain and item summaries in Tables S10c1–S10c8. Prognostic-factor analyses were assessed with the Quality in Prognosis Studies (QUIPS) tool, covering participation, attrition, prognostic-factor measurement, outcome measurement, confounding, and statistical analysis/reporting (Table S10d) [24]. Multivariable prediction-model studies were assessed with PROBAST + AI, the updated Prediction model Risk Of Bias ASsessment Tool (Table S10e), with signalling-question responses in Table S10f [25]. Development quality, evaluation risk of bias, and applicability were distinguished; the original PROBAST provides the methodological foundation for this separation [26]. Model-specific extraction was also informed by the Critical Appraisal and Data Extraction for Systematic Reviews of Prediction Modelling Studies (CHARMS) framework [27].

JBI instruments were applied to analytical cross-sectional, case-control, descriptive cohort, case-series, and uncontrolled before–after analyses [28,29,30,31]. The corresponding evidence and rationales are presented in Tables S10g–S10k; instrument versions are specified in Table S10l. Signalling questions and checklist criteria retained their instrument-specific response options; they were not converted into a common numerical quality score. Risk-of-bias judgments were distinguished from limited precision and restricted applicability. For prognostic analyses, confounding was evaluated against the relationship examined, including baseline neurological status, age, concomitant trauma, infection, medication, and relevant treatment exposure. Prediction appraisal additionally considered sample selection, predictor availability, endpoint definition, missing data, model complexity, optimism, calibration, and validation.

The formal assessments cover the analytical units identified in Tables S10b–S10k, rather than every secondary estimate. Secondary biomarker–pain or biomarker–function associations emphasized in the synthesis were additionally examined for selection and missingness, measurement and timing, confounding, and analytical or reporting limitations using the contextual criteria in Table S10a (Table S10m). This structured examination does not assign a new instrument score or extend a randomized-effect judgment to an observational association. Concurrent severity and case–control discrimination findings within prognostic reports were retained to distinguish their clinical targets; they were not treated as independently validated diagnostic tests or as evidence of later recovery.

R.S.C. and A.C. independently performed the critical appraisals before comparing their judgments against the extracted evidence. Disagreements were resolved through discussion and, when needed, adjudication by A.Q. Domain judgments, item responses and supporting reasons are reported in the supplementary tables. Missing methodological information was coded according to each instrument and was not interpreted as evidence of low risk. Table S11 links report-specific limitations to their implications for attribution, prognosis, precision and applicability. No certainty grade across study designs or summed quality score was calculated.

### 2.8. Synthesis Without Meta-Analysis and Evidence Interpretation

Reporting of the descriptive synthesis was informed by applicable items of the Synthesis Without Meta-analysis (SWiM) guideline [32], adapted to the evidence-mapping objectives of this scoping review. Reports were grouped by biomarker domain and clinical question, with injury phenotype, sampling window, and outcome definition used to determine comparability within groups. Grouping followed the review questions and eligibility criteria, with concurrent clinical associations, longitudinal prognosis and intervention-related comparisons considered separately.

Synthesis consisted of structured textual comparison supported by the evidence tables. For each clinical question, the narrative identified the contributing reports, the comparison examined, the assessment timing, and the findings reported by the original investigators. Numerical findings retained their published metrics, units, uncertainty, and analytical denominators. No common standardized effect metric was calculated, and no effect estimates were pooled or reanalysed. Findings addressing substantially different endpoints were described separately. Where a question was addressed by a single report, its findings were summarized individually without implying consistency across studies.

Interpretive emphasis was guided by directness to the review question, study design, and the result-specific appraisal. All eligible reports remained represented in the evidence map. Statistical significance was not used to select findings for inclusion or to determine the conclusions through vote counting. Cohort overlap was considered when assessing whether apparently concordant findings represented independent evidence.

Variation between findings was examined descriptively in relation to clinical phenotype, sampling time, specimen matrix, assay platform, outcome measurement, and intervention exposure. These characteristics provided context for differences between reports without establishing the causes of those differences. Partial integration was recognized when explicit analyses linked relevant domains, while joint measurement alone remained classified as co-measurement. Rehabilitation findings were interpreted using the reported distinction between prescribed and achieved exposure and the conditions under which performance was assessed.

Rehabilitation or physiological exposure was charted as prescribed and achieved treatment, including active time, intensity, repetitions, assistance, progression, adherence, interruptions, and concurrent care (Table S12). Single challenges were distinguished from repeated training, and assessment during assistance or stimulation was separated from immediate and sustained performance after cessation. No universal washout duration was assumed. No formal certainty grading was undertaken. Interpretation remained bounded by methodological limitations, clinical comparability, and cohort overlap; statements about evidence gaps applied to the included reports. A completed PRISMA-ScR checklist is provided as a separate document.

The descriptive sensitivity comparison was undertaken post hoc, after the initial synthesis, to examine the influence of the five case-by-case eligibility exceptions. Ahadi, Itodo, Seijas, Pu and Ding were jointly set aside. It examined support for domain- and outcome-specific interpretations in the remaining 36 reports, without recalculating participant-level estimates or pooled effects. The contributions and consequences of omitting each exception are summarized in Table S13.

## 3. Results and Discussion

### 3.1. Study Selection and Characteristics of the Evidence Base

**Figure 1:**
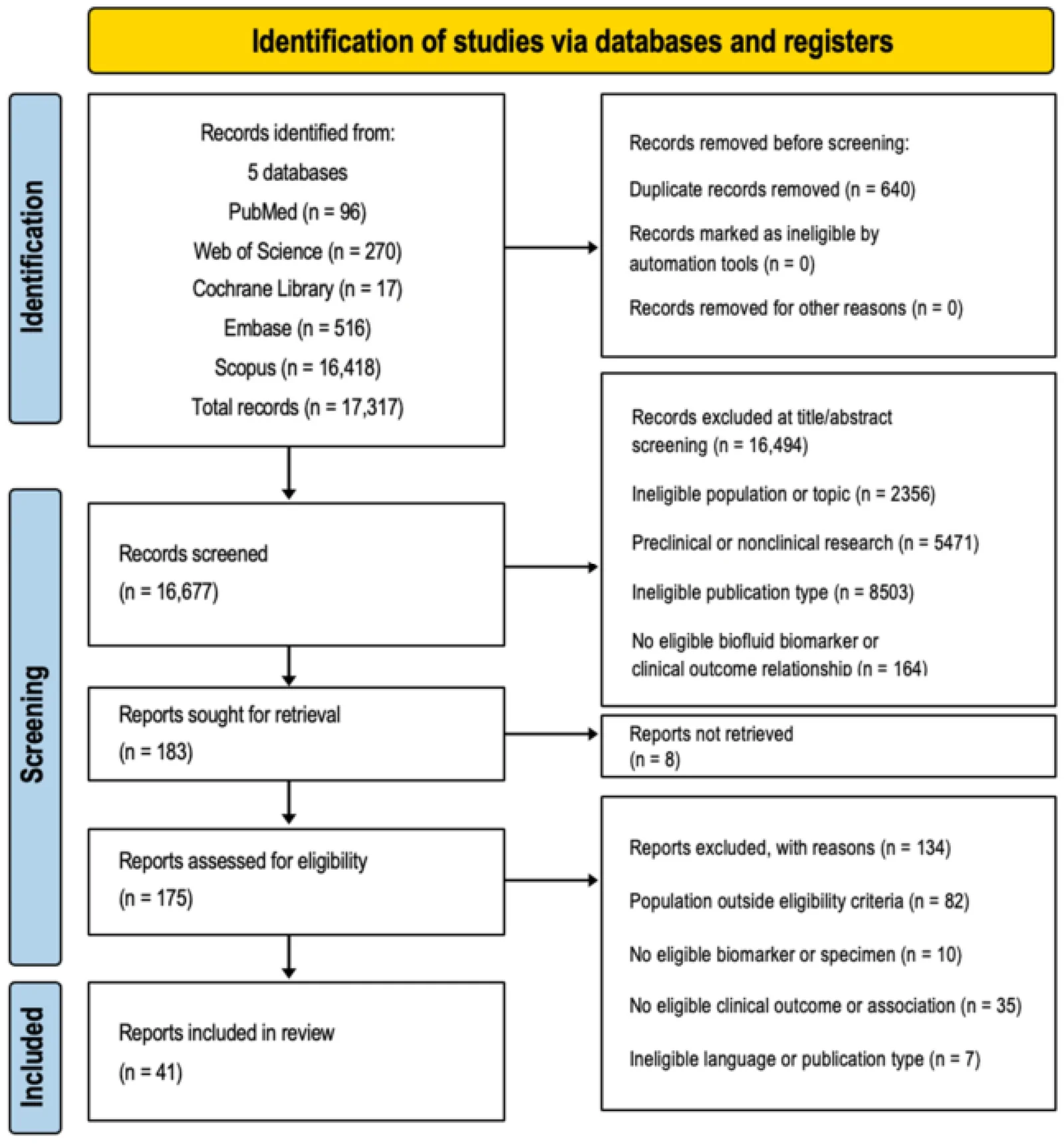
Selection of reports according to PRISMA. Identification and screening counts refer to records. Retrieval, eligibility, and inclusion counts refer to reports. The eight reports not retrieved are distinct from the 134 excluded after full-text assessment. The 41 included reports do not necessarily represent 41 independent participant cohorts. Abbreviation: PRISMA, Preferred Reporting Items for Systematic Reviews and Meta-Analyses.

The database searches identified 17,317 records: 96 from PubMed, 270 from Web of Science, 516 from Embase, 17 from the Cochrane Library, and 16,418 from Scopus. After removal of 640 duplicates, 16,677 records underwent title and abstract screening, of which 16,494 were excluded. Full texts were sought for 183 reports. Eight were not retrieved, leaving 175 reports assessed for eligibility. Of these, 134 were excluded because of the population (*n* = 82), biomarker or specimen (*n* = 10), clinical outcome or association (*n* = 35), or language and publication type (*n* = 7). Forty-one reports were included (Figure 1). Reports not retrieved were recorded separately from reports excluded after full-text assessment. Database-specific subtotals and the distinction between literature and registration records are documented in Table S4b.

The Scopus and PubMed totals reflect different indexed field scopes and term-processing rules, including reference metadata within Scopus all-fields retrieval. These platform characteristics provide context for unequal yields but do not establish the contribution of each field or explain the numerical difference quantitatively.

The included reports were published between 1997 and 2026. The exact number of independent participant cohorts represented by these 41 reports could not be established because several publications drew on overlapping recruitment programmes without sufficient information to resolve participant-level overlap (Table S6c). Two evaluated randomized interventions, while 39 contributed nonrandomized biomarker analyses. The latter category includes an observational biomarker analysis nested within a randomized care trial: randomization of clinical management does not randomize biomarker concentrations [33]. Table 1, Table 2 and Table 3 summarize the clinical populations, biomarker exposures, comparators, outcomes, analytical designs, and assessment times. Five reports assessed eligible neuroendocrine targets, 19 inflammatory targets, and 26 neural injury targets. Eight assayed eligible neurotrophic factors; two Kwon reports did not provide reliable quantitative growth-factor findings, leaving six reports with usable quantitative neurotrophic measurements [33,34]. These domain counts overlap and indicate measurement rather than a positive association or independent replication.

Clinical and analytical heterogeneity extended beyond biomarker identity. Acute traumatic SCI, chronic SCI, degenerative or compressive myelopathy, acquired tethering, combined neurotrauma, and perioperative tumour-related cord injury addressed different biological events and recovery opportunities. Serum, plasma, CSF, and CSF-derived exosome assays also represent distinct compartments and measurement processes. Accordingly, the synthesis proceeds by clinical question and biological domain, preserving endpoint-specific denominators and the distinction between concurrent association, prognosis, and randomized treatment effects.

Setting aside the five eligibility exceptions left 36 reports and narrowed the evidence map unevenly. Concurrent neural injury-marker severity comparisons and exploratory prognosis remained represented, as did inflammatory relationships with mobility and pain. The specific model combining CRP with the Brain and Spinal Injury Center (BASIC) MRI score was removed, although other biomarker–imaging and clinical combinations remained. Neuroendocrine coverage fell from five to three reports: the SwiSCI androgen trajectories and androgen–SCIM-III analyses were no longer represented. Other reports retained Functional Independence Measure (FIM) or SCIM-III assessments, principally with nonsignificant biomarker associations, but did not provide an alternative adrenal-androgen–independence analysis. No report in the narrower subset established biomarker-guided rehabilitation dosing or a stress-related endocrine pathway linked to independently assessed neuroplasticity. The comparison therefore preserved these clinical boundaries while reducing the breadth of the endocrine and independence-related mapping (Table S13).

### 3.2. Risk of Bias and the Strength of Clinical Inference

#### 3.2.1. Randomized Treatment-Assignment Contrasts

**Figure 2:**
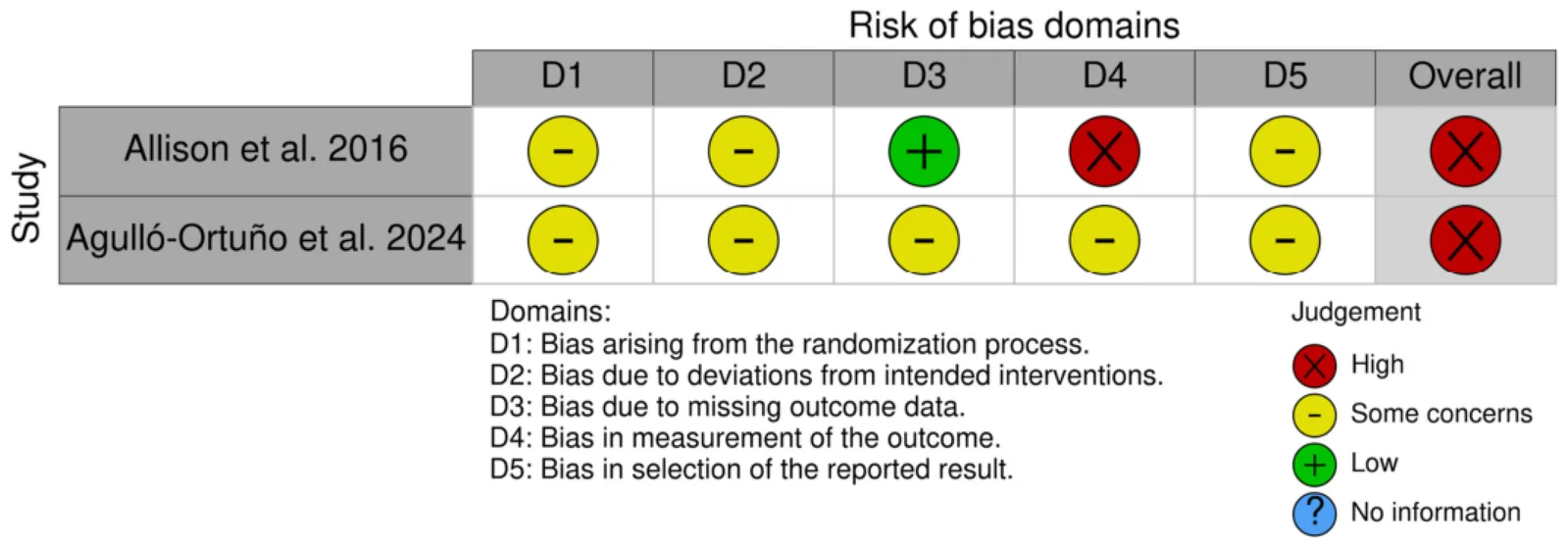
Result-specific risk of bias for the randomized treatment-assignment contrasts. Each report appears once. Allison et al. (2016) compare diet and supplements with usual diet for sensory neuropathic pain measured using the NPQ at 12 weeks [38]. Agulló-Ortuño et al. (2024) compare early with late aquatic-therapy sequences for 10MWT performance at weeks 6 and 12 [42]. Green “+” denotes low risk, yellow “−” some concerns, and red “X” high risk. The blue “?” in the legend denotes unavailable information; it is not a fourth RoB 2 risk category and is not used in the matrix. Footnote: D1, bias arising from the randomization process; D2, bias due to deviations from intended interventions; D3, bias due to missing outcome data; D4, bias in measurement of the outcome; D5, bias in selection of the reported result. Overall includes the additional period/carryover domain for the crossover comparison; therefore, Agulló-Ortuño’s overall high-risk judgment can coexist with some concerns in D1–D5. Judgments concern randomized treatment-assignment contrasts. Observational biomarker–outcome associations require separate appraisal. Abbreviations: 10MWT, 10-metre walk test; NPQ, Neuropathic Pain Questionnaire; RoB 2, revised Cochrane risk-of-bias tool for randomized trials.

The randomized evidence comprised Allison’s dietary intervention and Agulló-Ortuño’s early versus late aquatic-therapy sequence [38,42]. Four outcome-specific appraisals were completed across these two reports. Figure 2 displays one selected clinical result per report in chronological order: sensory neuropathic pain measured with the Neuropathic Pain Questionnaire (NPQ) at 12 weeks, and 10-metre walk test (10MWT) performance at weeks 6 and 12. Each overall judgment applies to that selected result; it is not a global score for the article or its biomarker findings.

For Allison’s sensory pain result, D1–D5 were judged as some concerns, some concerns, low risk, high risk, and some concerns, respectively, giving an overall high risk of bias. The subjective, unblinded pain assessment was the principal concern. The corresponding IL-6 and CRP result had low risk for outcome measurement and an overall judgment of some concerns. All 20 randomized participants completed follow-up. Agulló-Ortuño’s clinical result had some concerns in all five standard domains; the overall high-risk judgment arose from the crossover-specific period/carryover domain. Training-related functional gains could persist into the subsequent usual-therapy period, complicating attribution to the assigned sequence. The cytokine result had an overall judgment of some concerns. Forty participants supplied complete biomarker data from 50 randomized participants; this biomarker denominator cannot be transferred to functional endpoints with fewer observations. The domain judgments and outcome-specific reasons are presented in Tables S10b and S10b2.

#### 3.2.2. Nonrandomized Associations and Prediction Models

**Figure 3:**
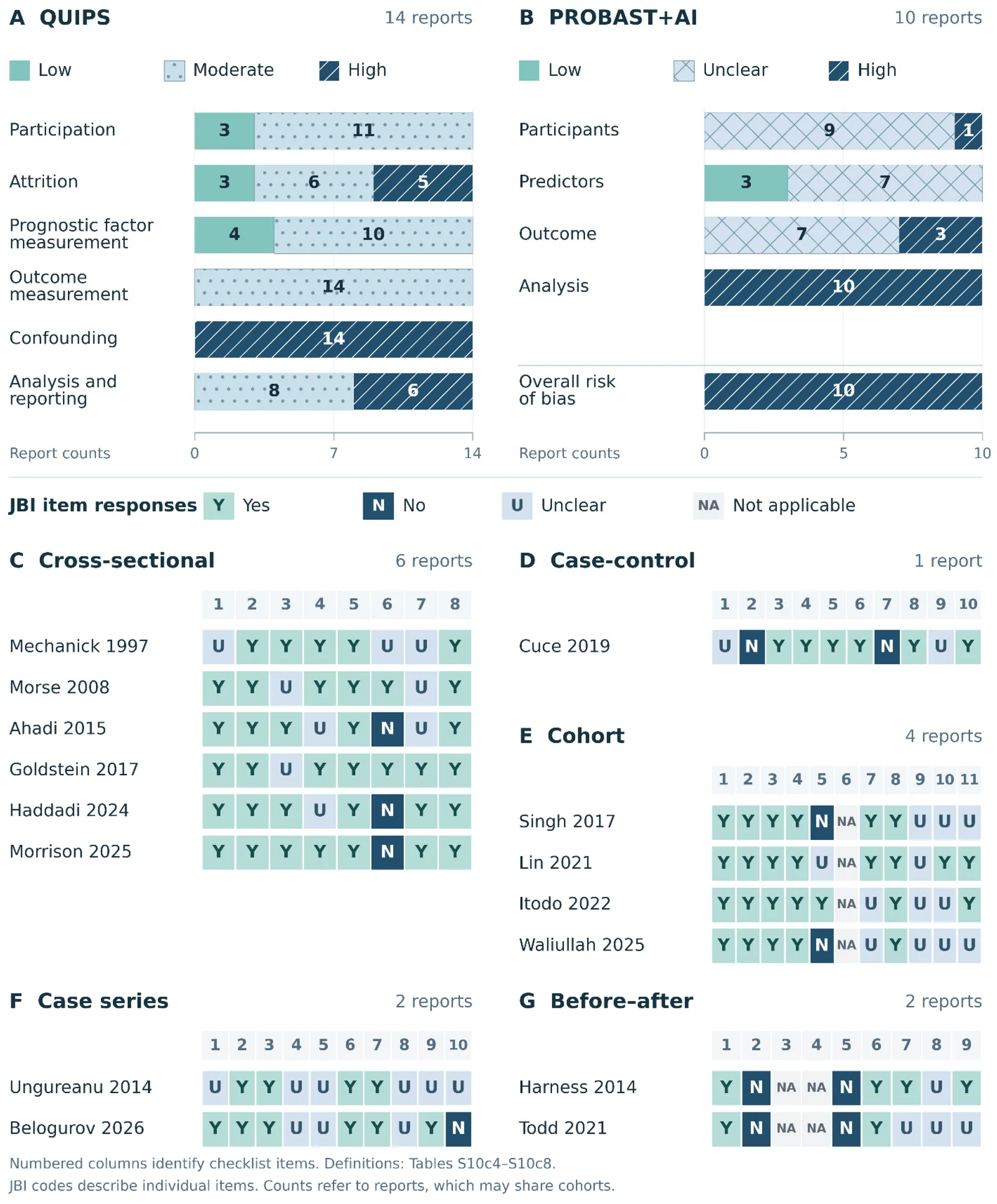
**Critical appraisal of nonrandomized evidence. The figure summarizes the primary reviewed analytical unit from each of 39 reports.** (**A**) QUIPS domain-level risk-of-bias judgments for 14 prognostic-factor reports, covering study participation, study attrition, prognostic factor measurement, outcome measurement, study confounding, and statistical analysis and reporting. (**B**) PROBAST + AI evaluation risk-of-bias judgments for 10 prediction-model reports, covering participants, predictors, outcome, and analysis, with Overall presented separately. Development-quality and applicability assessments are not plotted. Bar labels are absolute report counts, summing to 14 in each QUIPS domain and 10 in each PROBAST + AI domain or overall assessment. (**C**–**G**) JBI item responses for analytical cross-sectional reports ((**C**), *n* = 6) [17,35,36,39,53,66], a case-control report ((**D**), *n* = 1) [40], cohort reports ((**E**), *n* = 4) [18,46,57,63], case series ((**F**), *n* = 2) [45,68], and uncontrolled before–after reports assessed with the quasi-experimental checklist ((**G**), *n* = 2) [37,41]. Reports are ordered chronologically within each matrix. Judgments correspond to Tables S10c2–S10c8, with appraisal units, supporting reasons, and instrument versions detailed in Tables S10d–S10l. Appraisal categories retain their instrument-specific meanings and should not be interpreted as a common quality scale. Counts refer to reports and do not necessarily represent independent cohorts. Abbreviations and response codes: QUIPS, Quality in Prognosis Studies. PROBAST + AI, the updated Prediction model Risk Of Bias ASsessment Tool for prediction models using regression or artificial intelligence. JBI, formerly the Joanna Briggs Institute. Q, checklist question. Y, Yes. N, No. U, Unclear. NA, Not applicable. JBI responses indicate whether individual criteria were met and are not overall risk-of-bias grades. Numbered columns correspond to checklist-specific questions, with detailed definitions and applicability notes provided in Tables S10c4–S10c8 for Panels C–G, respectively.

Among the 39 nonrandomized reports, the principal analytical units were assessed using QUIPS in 14, PROBAST + AI in 10, and JBI instruments in 15. Figure 3 summarizes these domain-level judgments and checklist-specific item responses. The JBI allocation comprised six analytical cross-sectional reports, one case-control report, four descriptive cohorts, two case series, and two uncontrolled before–after experiments. Each report contributes once to this allocation according to the relationship emphasized in Table S9; these counts are neither a count of all assessed results nor a replacement for the recruitment-design descriptions in Table 1, Table 2 and Table 3. Material secondary biomarker–pain and biomarker–function associations received a separate structured examination (Table S10m). Their interpretation does not inherit the tool judgment for the primary randomized contrast or prognostic model.

All 14 QUIPS appraisals identified high risk in the confounding domain. Associations between an early marker and a later outcome were frequently insufficiently separated from baseline neurological severity, injury phenotype, treatment, or clinical complications. All 10 prediction-model reports were judged at high risk of bias overall, driven by analytical concerns. These included model development relative to the available outcome events, incomplete handling of missingness, predictor selection, and inadequate correction or evaluation of optimism. High risk was not assigned solely because a study was small or lacked external validation. Development quality and clinical applicability were assessed separately. JBI item responses identified design-specific weaknesses without a summed score or an imposed overall low/high category.

This appraisal pattern limits the interpretation of both positive and null findings. A significant marker–outcome relationship may reflect injury severity or selected survivors, while a null estimate from a small sample may remain compatible with clinically relevant effects. Precision should be assessed through the analytical denominator and uncertainty interval, rather than inferred from the *p* value [69]. Similarly, biomarker qualification, prognostic-factor association, multivariable prediction, and prediction of treatment benefit are distinct stages of evidence development [70,71,72,73,74]. The following thematic synthesis preserves these distinctions.

### 3.3. Neural Injury Biomarkers and Neurological Outcomes

#### 3.3.1. Concurrent Injury Severity and Sampling Dependence

Acute studies most consistently examined whether neural injury proteins distinguished neurological severity at or near sampling. Serum, plasma and CSF assessments of glial fibrillary acidic protein (GFAP), neurofilament measures, tau, S100 calcium-binding protein B (S100B), and neuron-specific enolase (NSE) showed severity-dependent signals, but the observed differences depended on specimen timing and the clinical grouping [17,33,43,44,45,46]. Such contrasts support investigation of injury burden; they do not establish prediction of recovery beyond the baseline examination. In Kwon’s initial CSF study, the classification analysis concerned initial severity, whereas the motor-outcome analysis used a smaller cervical subgroup [33]. Pouw’s 16-person analysis and Ahadi’s series, whose SCI-specific age range was not reported, likewise require interpretation in light of small severity strata, follow-up availability, and competing mortality [17,44].

Later studies reinforced the importance of keeping diagnostic, severity, and prognostic comparisons separate. In Haddadi’s 31-person severity comparison (nine complete and 22 incomplete injuries), serum and CSF S100B yielded areas under the receiver operating characteristic curve (AUCs) of 0.586 and 0.823, respectively. Numerical AUC uncertainty was not reported, and the serum–CSF correlation was weak and nonsignificant [53]. In Azad’s mixed clinical cohort, circulating cell-free DNA distinguished SCI cases from healthy controls, whereas the conversion model involved only 34 endpoint-assessed participants and a combined predictor panel [54]. Diagnostic discrimination against healthy controls cannot substitute for prognosis within a clinically heterogeneous SCI population. Concurrent inflammatory transcript and protein measurements in polytrauma also require separation from effects of associated injuries [66].

Serial marker concentrations are potentially informative, but a changing biochemical trajectory is not itself evidence of neurological restitution. Assay sensitivity and dynamic range affect whether a target is quantifiable; ultrasensitive single-molecule methods can extend detection without establishing clinical validity [75]. Findings obtained using different matrices, platforms, or below-detection conventions therefore cannot be treated as directly interchangeable. Blood neurofilament light chain (NfL) also has determinants beyond neural injury; work outside SCI demonstrates effects of blood volume and body mass index, which motivates explicit examination of these influences in SCI validation cohorts [76].

#### 3.3.2. Subsequent Neurological Status and Prediction of Change

Longitudinal neural injury findings provided stronger temporal ordering but heterogeneous endpoints. In Kwon’s larger CSF study, 48 participants contributed six-month AIS-conversion outcomes, including 22 converters and 26 non-converters. The inflammatory-index AUC was 0.883 (95% confidence interval [CI], 0.781–0.985), and the structural-index AUC was 0.857 (95% CI, 0.749–0.964). The reported standard errors were 0.052 and 0.055, respectively [34]. These AUCs were estimated in the development sample. Leave-one-out classification evaluated related discriminant models and did not provide independent external validation. Targeted proteomics identified numerous baseline severity associations, but no AIS-conversion association survived false-discovery-rate correction. Factor XII was associated with motor change after the reported adjustment [47]. This divergence illustrates why severity discrimination and recovery prediction cannot be combined under a single prognostic label. Multiplicity-adjusted analyses should retain their distinction from exploratory nominal associations [77].

The largest paired-fluid investigation included 118 participants, with 113 completing follow-up, and evaluated NfL and GFAP against defined neurological outcomes [51]. Among 74 initially AIS A participants, a 72-h serum NfL threshold of 264 pg/mL identified nonconversion with sensitivity 76% (95% CI, 61–87%) and specificity 77% (95% CI, 55–92%). In a cervical motor-outcome analysis involving 65 participants, a 170 pg/mL threshold identified limited motor gain of no more than eight points with sensitivity 88% (95% CI, 69–97%) and specificity 91% (95% CI, 75–98%). These endpoint-specific estimates are promising research signals, but the thresholds were internally derived and have not been established as routine treatment-selection cut-offs. Repeated CSF and serum samples were observations, not additional independent participants.

Smaller studies showed less consistent or less transportable signals. NSE and S100B discriminated later improvement groups in one cohort, but substantial baseline group differences complicated attribution [48]. Rieder found no significant association between the measured serum targets and six-month AIS or FIM outcomes [49]. Two Wichmann reports drew on the same 15-person cohort; only 11 had follow-up, so they do not represent independent replications [64,65]. Yang’s analyses were predominantly null apart from a time-specific serum tau finding, while Waliullah’s serial S100B report contained internally inconsistent concentration summaries [52,57]. Raheja’s neurological follow-up involved nine of 26 enrolled participants after deaths and losses, making apparently strong subgroup discrimination especially sensitive to selection [56].

Recent multivariable models extend this field but remain developmental. Fraussen’s baseline NfL–GFAP–contactin-1 panel yielded an AUC of 0.863 (95% CI, 0.731–0.994) for AIS conversion and 0.857 (95% CI, 0.707–1.000) for a motor-score gain of at least five points. The reported outcome groups totalled 33 and 29 participants, respectively, but complete-case ROC denominators were not separately stated, and reported motor-score availability was inconsistent. Outcomes were taken from the latest available clinical assessment, and cross-validation was not performed [55]. In Hei’s 71-person cohort (38 converters and 33 non-converters), baseline AIS, MRI lesion length, and tau produced an apparent AUC of 0.936 (95% CI, 0.871–1.000). The specimen was charted as serum because the collection and assay Methods explicitly describe serum. Conflicting plasma terminology in the title and Results and inconsistent follow-up wording remain reporting limitations [58]. Sheinberg’s CSF-exosome protein model yielded an improvement AUC of 0.813 in a 25-participant cohort, without numerical AUC uncertainty or a clearly specified fitted-model denominator. A separate miRNA confirmation cohort did not externally validate that protein model [59]. Pu’s combined SCI–traumatic brain injury cohort used the Glasgow Outcome Scale, which cannot isolate spinal neurological recovery [20]. These distinctions prevent recent publication date or high apparent discrimination from being mistaken for established clinical utility.

Prediction performance must ultimately be evaluated across complementary dimensions. Internal resampling can estimate optimism, provided that predictor selection and other model-building steps are repeated within the resampling procedure [78]. Sample-size planning should address the number of candidate parameters, outcome frequency, and anticipated model performance [79,80]. Discrimination measures ranking, calibration assesses agreement between predicted and observed risks, and decision-curve analysis can examine the consequences of using a model at clinically relevant thresholds [81,82]. External evaluation in an appropriate independent population is required before a model can inform practice [83]. None of these requirements can be replaced by statistical significance alone.

### 3.4. Inflammatory Biomarkers, Mobility and Neuropathic Pain

#### 3.4.1. Acute Inflammation and Chronic Clinical Phenotypes

Inflammatory targets linked acute injury biology with systemic clinical states, but their interpretive meaning varied by phase. Acute CSF cytokines contributed to severity and outcome panels [33,34,47]. In contrast, chronic CRP studies primarily examined concurrent mobility and adiposity. Morse reported adjusted geometric mean CRP concentrations of 5.11 mg/L among motorized-wheelchair users, 2.19 mg/L among manual-wheelchair users, 1.41 mg/L among assisted walkers, and 1.63 mg/L among independent walkers [36]. Goldstein’s larger analysis found mobility-related trends in most adiposity-adjusted models, while injury level and completeness did not independently explain CRP [39]. Twenty-six participants from the earlier report were reassessed in the later cohort, limiting independence of the evidence.

These findings are compatible with an inflammatory phenotype related to mobility, body composition, and systemic health; their cross-sectional design cannot establish whether inflammation restricts mobility or mobility-related exposures alter inflammation. CRP is consequently insufficient as a stand-alone measure of spinal repair. Clinical evaluation should retain infection, concomitant trauma, adiposity, and medication as alternative explanations. Ding’s acute cervical model combined peripheral inflammation and MRI, but its one-year AIS A–C versus D–E outcome represented subsequent neurological status rather than conversion from baseline [21]. Among 374 participants (147 with one-year AIS A–C and 227 with AIS D–E), the CRP AUC was 0.833 (95% CI, 0.792–0.874), compared with 0.958 (95% CI, 0.939–0.976) for the BASIC MRI score. The combined model had an apparent AUC of 0.973 (95% CI, 0.959–0.987). Unresolved serum/plasma labelling further restricts transfer of its concentrations to a matrix-specific clinical threshold.

#### 3.4.2. Pain Associations and Intervention-Related Findings

Randomized dietary treatment produced a group-by-time effect for sensory neuropathic pain (*p* < 0.01; reported Cohen’s d = 1.29), whereas between-group changes in IL-6 and CRP were nonsignificant (*p* = 0.13 and *p* = 0.10) [38]. An exploratory inflammatory-change model explained part of the variation in pain change, but its predictors and outcome were changes over the same interval. Backward selection among multiple inflammatory targets in 20 participants, incomplete adjustment for treatment assignment and clinical factors, and unblinded pain reporting limit this observational association independently of the trial’s randomized contrasts. Adjustment for depressive-symptom change does not establish causal mediation. The clinical and laboratory results therefore require separate interpretation (Table S10m).

The aquatic-therapy trial found no between-sequence differences in cytokine concentrations at individual assessment times, although IL-8 decreased within both sequences [42]. Comparisons of changes across corresponding treatment periods yielded differences for IL-1β, IL-4 and IL-12p70 during combined aquatic and usual therapy, and for TNF-α, IL-4, IL-12p70, IL-1β and MCP-1 during usual therapy alone. These period-specific findings should not be interpreted as a globally null inflammatory response or as an isolated effect of aquatic therapy, given the evolving injury phase and possible carryover. Within the late sequence, baseline-to-week-12 IL-8 change correlated with 10MWT change (Pearson’s r = −0.776, *p* = 0.003), but the pairwise analytical denominator was not reported, and functional measurements involved fewer participants than the 40 complete biomarker cases. This association remains observational despite its trial setting. The correlation was unadjusted and arose within an extensive set of cytokine–function comparisons with selective display of significant findings, further limiting a mechanistic or treatment–response interpretation (Table S10m). Lin’s genotype-stratified analysis also illustrates heterogeneity: IL-8 varied with time and injury severity, while the reported pain-related signal concerned IL-13 rather than establishing IL-6 or IL-8 as pain predictors [63]. Extensive cytokine missingness further qualifies those comparisons.

A single exercise challenge linked baseline IL-1 receptor antagonist to immediate pain change in eight participants (r = 0.833, *p* = 0.01), but the association did not persist at 90 min [41]. The baseline-factor association was unadjusted for lesion phenotype, cannabis use or achieved exercise intensity and was examined among multiple cytokine–response comparisons (Table S10m). It does not establish differential treatment benefit or durable training benefit. Clinical pain classification and established rehabilitation guidance remain necessary because neuropathic pain has distinct sensory, affective, interference, and temporal components [84,85]. A useful biomarker would need to add actionable information beyond these assessments. Differences between statistically significant and nonsignificant subgroup results are insufficient evidence of an interaction; treatment-effect modification requires a direct interaction analysis [86]. No included report established a baseline biomarker as a selector of randomized rehabilitation benefit.

### 3.5. Neuroendocrine Physiology, Stress and the HPA Axis

#### 3.5.1. Distinct Endocrine Constructs After SCI

The five neuroendocrine reports examined basal endocrine status, stimulated adrenal responses, acute exercise physiology, and androgen–function relationships rather than a common HPA-axis endpoint [18,19,35,37,40]. Mechanick’s retrospective findings did not establish cortisol as a predictor of subsequent neurological recovery. In chronic SCI with neuropathic pain, Cuce distinguished responses to 1 µg and 250 µg ACTH challenges. Within the 15 participants with neuropathic pain, peak cortisol correlated with pain intensity after both challenges (Spearman’s r = 0.52, *p* = 0.047, and r = 0.58, *p* = 0.025, respectively) [40]. These unadjusted within-group associations remain distinct from the case–control comparison and were examined separately for confounding and multiplicity (Table S10m). They describe concurrent pain–adrenal-response relationships and do not establish that psychological stress causes pain or that cortisol mediates recovery.

During inpatient rehabilitation, men in Itodo’s cohort showed an adjusted DHEAS increase of 1.77 µmol/L (95% CI, 0.73–2.81) [18]. Baseline injury completeness was also associated with androgen status, but the report did not test whether endocrine recovery explained neurological improvement. Recruitment above age 16, missing baseline neurological data, sex-specific estimates, and blood sampling between 07:00 and 14:00 constrain applicability. Harness’s single exercise session showed no significant overall cortisol response and included a smaller cortisol-specific sample [37]. Basal cortisol, stimulated secretion, and adrenal androgen change therefore cannot be combined as equivalent measures of stress regulation.

Seijas linked serum androgens to Rasch-transformed SCIM-III functioning in 80 SwiSCI participants [19]. Baseline blood sampling occurred at a median of 42 days after SCI (IQR 30–54), placing the typical sample in the subacute phase under this review’s definitions despite the article’s acute-phase label. In the fully adjusted discharge analysis, the reported percentage differences per one-standard-deviation increase were 0.2% for DHEAS (95% CI −24 to 20, *p* = 0.99) and 2.5% for DHEA (−17 to 19, *p* = 0.79). The corresponding free-testosterone estimate was 26.9% (9 to 42, *p* = 0.01), but testosterone does not independently satisfy this review’s biomarker criteria. These percentages derive from the study’s transformed functioning model and are not absolute SCIM points or effects of hormone treatment. Subgroup-dependent adrenal androgen findings, incomplete control for neurological severity, variable blood-sampling times and possible overlap with Itodo limit causal and independent-replication interpretations.

#### 3.5.2. The Unresolved Link Between Stress Biology and Neuroplasticity

The exploratory review question was not resolved by an eligible human analysis connecting stress-related HPA measurements with an independently assessed neuroplasticity outcome. Cortisol concentrations and ACTH responses describe endocrine physiology; they do not independently quantify perceived stress, stress exposure, or neural reorganization. General HPA physiology supports mechanistic plausibility [8], and experimental glucocorticoid effects on learning-associated synapses provide a rationale for future SCI research [9]. However, those experimental findings are not clinical evidence of an HPA-mediated rehabilitation effect after SCI.

Work outside SCI has linked psychosocial stress to reversible changes in prefrontal processing and attentional control [87]. It does not establish the same pathway in injured spinal systems. Future studies could combine a validated perceived-stress instrument with appropriately timed endocrine sampling; the Perceived Stress Scale is one established measure of the psychological construct [88]. A standardized psychosocial challenge such as the Trier Social Stress Test addresses a different question from an ACTH adrenal challenge [89]. Neither measure was treated as having been administered in the included SCI reports. A credible stress–plasticity analysis would additionally require independent physiological or structural measures, temporal separation of exposure and outcome, and control of pain, sleep, medication, autonomic phenotype, and concurrent rehabilitation.

### 3.6. Neurotrophic Factors and the Limits of a Plasticity Interpretation

The neurotrophic evidence was heterogeneous and did not support a circulating factor as a direct measure of neural reorganization. Early Kwon studies assayed growth-factor targets without reliable quantitative results [33,34]. Harness found no significant circulating BDNF response across a two-hour activity-based bout (*p* = 0.97) [37]. Rieder’s serum NGF and GDNF findings were not significantly associated with six-month AIS or FIM outcomes [49]. These null results apply to the targets, assays, timing, and endpoints tested; they do not exclude activity-dependent plasticity in other compartments or over longer training periods.

Positive findings were exploratory and clinically distinct. Capirossi linked early CSF BDNF with discharge motor status and motor change, but the nine-person analytical sample, multiple unadjusted comparisons, and discharge windows ranging from 47 to 320 days limited prognostic inference [50]. Wichmann reported a plasma GDNF association with conversion after false-discovery-rate correction, whereas CSF GDNF was mostly below detection [64]. In degenerative cervical myelopathy, BDNF increased postoperatively, but the reported association with pinch-strength change concerned NfL rather than validating BDNF-mediated plasticity [67].

Biological source and preanalytical conditions are particularly relevant for BDNF. Human platelets store and release BDNF, and commercial assays can produce differing serum measurements [90,91]. Evidence of cerebral BDNF release during exercise outside SCI provides mechanistic context, but it does not make a peripheral venous concentration a specific readout of spinal synaptic adaptation [92]. The clinical next step is to test whether a defined, reproducibly measured neurotrophic signal is associated with an independent plasticity measure and an outcome meaningful to patients. An increase in concentration alone cannot establish that sequence.

### 3.7. Imaging, Residual Neurological Function and Distinct Cord Phenotypes

#### 3.7.1. Partial Integration with Imaging and Physiological Measures

The included reports provide evidence of partial integration, particularly biomarker–MRI models and models incorporating the sensory zone of partial preservation [21,51,58]. This is substantive progress beyond isolated biochemical measurement. Nevertheless, simultaneous acquisition does not establish a molecular–circuit–functional pathway unless the relationships between its components are explicitly tested. Supplementary Figure S1 maps biomarker-domain measurement coverage across the 41 reports, distinguishing quantitative measurements from assays without reliable quantitative findings. Table S8b distinguishes examined clinical associations from co-measurement and specifies the imaging or residual-function relationships actually tested.

MRI describes structural injury, compression, and preserved substrate. Established imaging studies relate acute lesion characteristics, axial BASIC scores, and postoperative lesion length to neurological outcomes [93,94,95]. Motor-evoked potentials provide a complementary assessment of descending transmission [96]. A sensory zone of partial preservation, an anatomical lesion score, and an electrophysiological response therefore contribute different information. Adding a fluid marker to one of these measures should be evaluated for incremental predictive performance, not presumed to demonstrate a circuit mechanism.

Longitudinal imaging studies outside the eligible biomarker corpus show that remote atrophy, degeneration, and cortical reorganization can be assessed independently after SCI [97,98,99]. These reports contextualize possible plasticity endpoints without being added to the 41-report evidence map. Structural loss and cortical recruitment are also non-equivalent phenomena. A future study should specify whether its independent endpoint represents degeneration, altered connectivity, physiological transmission, or adaptive task recruitment before testing a fluid biomarker relationship.

#### 3.7.2. Myelopathy, Tethering and Perioperative Cord Injury

Non-traumatic and perioperative phenotypes require separate interpretation because their timing, baseline reserve, and treatment differ from acute traumatic SCI. In compressive myelopathy, pNF-H was higher in patients with acute neurological worsening, but its association with six-month JOA recovery was nonsignificant [60]. Kato’s pNF-H-positive participants had worse baseline myelopathy scores without a significant difference in postoperative recovery rate [61]. Thus, an injury-related signal in these populations may characterize baseline disease burden more clearly than recovery potential. Vedantam’s later work identified exploratory postoperative functional associations, but a small cohort cannot establish a transferable clinical algorithm [67].

Acquired tethering was associated with CSF neural injury elevations, while relationships with clinical scales were not significant [62]. In perioperative tumour-associated injury, inflammatory measurements and intraoperative monitoring were obtained in a small human cohort, but surgery and corticosteroid exposure complicated attribution [68]. The accompanying rodent pharmacological findings were excluded from the human clinical synthesis. Similarly, cell-culture findings accompanying CSF-exosome analyses do not demonstrate human neuroplasticity [59]. Keeping these clinical contexts distinct broadens the map while preventing a common SCI label from implying shared prognosis or mechanism.

### 3.8. Rehabilitation Exposure and Clinically Meaningful Outcomes

#### 3.8.1. Delivered Exposure and the Interpretation of Biological Change

Rehabilitation exposure was incompletely quantified. Agulló-Ortuño prescribed 18 aquatic sessions of 40 min over six weeks; Harness studied one two-hour activity-based bout; Todd studied one 30-min arm-crank bout [37,41,42]. These schedules do not establish participant-specific completed active minutes, achieved repetitions, or a common training dose. Rieder reported rehabilitation frequency without sufficient session-duration detail, and Allison’s mean self-reported dietary adherence of 89% addressed a different exposure [38,49]. Table S12 retains prescribed exposure, reported adherence, assistance, progression, interruptions, and concurrent care separately.

SCI exercise guidelines define clinical exercise prescriptions, but guideline-based scheduling does not establish the delivered exposure in an individual biomarker study [100]. Intensity, active practice, assistance, and rest influence both physiological responses and task performance. A baseline marker may be associated with the capacity to complete treatment, rather than with its causal effect. Prospective rehabilitation analyses should therefore distinguish prognostic association with outcomes under usual care from an interaction indicating differential benefit of randomized treatment [74,86]. The included reports did not establish a validated biomarker-guided dosing strategy or a formally tested molecular mediation pathway.

#### 3.8.2. Neurological Change, Task Performance and Independence

Endpoints occupied different clinical levels. Initial AIS grade describes impairment; AIS conversion is a categorical neurological change; motor-score gain quantifies another aspect of neurological recovery. Walking speed, pinch strength, and other task measures concern performance, while SCIM and total FIM concern independence. Established neurological and functional instruments should retain these distinctions [3,4,5]. Natural recovery varies across injury phenotypes, and the clinical meaning of a numerical score change depends on its distribution and functional consequences [101,102,103]. Accordingly, a model for later AIS status cannot be assumed to predict independent walking or daily independence.

Participation, quality of life, cognition, and learning were not established as biomarker-linked recovery outcomes across this corpus. Acute affect/arousal measures, an affective pain subscale, and total FIM do not substitute for dedicated assessments of these constructs. Patient-reported outcome systems such as SCI-QOL offer relevant domains for future studies, but their inclusion should follow the clinical question rather than extend an unsupported biomarker claim [104]. A clinically useful validation programme would prespecify the endpoint and a meaningful horizon, document its completeness, and retain the biomarker and clinical analytical denominators separately.

Neuromodulation research provides context for distinguishing assisted performance from persistent recovery. Brain–spine interfaces and non-invasive stimulation combined with structured rehabilitation have demonstrated clinically relevant functional gains in separate investigations [105,106]. These papers are contextual references, not additional eligible fluid-biomarker reports. Future integration should distinguish performance while assistance or stimulation is active, immediate assessment after cessation, and sustained benefit across repeated assessments without the device. No universal washout duration is assumed. These distinctions preserve patient benefit while identifying the specific evidence needed to attribute durable neural adaptation.

### 3.9. Clinical Relevance and Priorities for Validation

Recent reviews have synthesized acute SCI serum or biofluid biomarkers, emphasizing tissue-injury signals, sampling heterogeneity, and prognostic opportunities [107,108]. The present review contributes a complementary relationship-based map across neuroendocrine, inflammatory, neurotrophic, and neural injury domains, with rehabilitation relevance defined by the actual analytical question. Its added value is the explicit separation of initial severity, future status, change from baseline, independence, stress-related endocrine physiology, and randomized treatment effects. It also identifies existing partial multimodal integration rather than requiring every biological domain to be measured in a single cohort.

In current practice, these findings support careful interpretation of a biological measurement alongside the neurological examination, injury phenotype, sampling window, complications, and treatment exposure. They do not justify using an internally derived concentration threshold to select rehabilitation intensity, deny treatment, or infer neuroplasticity. The most credible translational role at present is research stratification and prospective evaluation of incremental information beyond established clinical and imaging predictors. A biomarker’s clinical role should be specified using the distinction between diagnostic, prognostic, monitoring, response, and predictive functions [70].

Future studies should define the intended decision and target population before selecting markers. Models need reproducible assays, prespecified predictor availability, suitable sample sizes, clinically relevant outcomes, and assessment of discrimination, calibration, and net benefit. TRIPOD and its updated TRIPOD + AI guidance support transparent reporting of model development and evaluation [109,110]. Model-focused evidence syntheses can additionally use TRIPOD-SRMA, while recognizing that the present work is a broader scoping review rather than a meta-analysis of model performance [111]. Independent validation should precede clinical implementation, and randomized interaction analyses are needed before a marker can direct treatment choice.

### 3.10. Strengths and Limitations

The review retains null findings, report-specific populations, biological matrices, exact temporal relationships, and outcome-specific analytical denominators. It makes cohort dependence explicit and applies critical-appraisal instruments appropriate to each analytical question. These features reduce interpretive overreach but do not remove the limitations of the original evidence. Most prognostic-factor analyses were vulnerable to confounding, and all included prediction-model reports had high overall risk of bias. Several studies combined high-dimensional panels with small outcome samples, incomplete follow-up, or inconsistently reported endpoint definitions. Apparent agreement between overlapping reports cannot establish independent replication.

Coverage was constrained by selected biomarkers and mandatory matrix and clinical-outcome search concepts. Reports may be missed when a named target, specimen or eligible clinical relationship is described only in the full text. The October reformulation altered both terminology and field scope and does not establish retrieval sensitivity. All-fields searching can retrieve terms from more metadata fields, including Scopus reference fields, while also reducing precision. The proportion of eligible literature missed remains unquantified. The scope was amended after protocol registration and after an earlier evidence map, and eight reports could not be retrieved. The distribution of these eight reports across biomarker domains and clinical populations could not be established, preventing assessment of whether retrieval failures were concentrated in a particular domain or population. Future updates could strengthen retrieval through formal review of the search strategy using PRESS [112]. English-language eligibility and explicitly retained age-related, combined-neurotrauma and unresolved-specimen exceptions also restrict generalizability. The SwiSCI reports address complementary questions but may reuse participants, so their sample sizes and findings cannot be treated as independent replication. These retrieval restrictions and limitations of the primary reports constrain the completeness and interpretation of the evidence map. The sensitivity comparison identifies which interpretations depend on the retained exceptions; it does not remove the possibility of discretionary selection introduced by case-by-case retention.

Missing observations were consequential, especially when survival, complications, or capacity to complete functional testing affected follow-up. Multiple imputation is potentially useful when supported by defensible assumptions, but it does not by itself correct informative missingness or unreported selection [113,114]. No participant-level reanalysis, pooled treatment effect, or formal certainty grading was undertaken. The descriptive synthesis follows relevant SWiM reporting principles while retaining the broader evidence-mapping purpose [32]. Absence statements therefore concern the included reports and the defined scope. The proposed molecular–circuit–functional framework specifies testable links; it is not a validated causal pathway or a clinical treatment algorithm.

## 4. Conclusions

Selected fluid biomarkers after spinal cord injury provide relevant signals of tissue injury, inflammation, endocrine physiology, and neurotrophic activity. Their interpretation depends on the clinical phenotype, biological matrix, sampling window, assay, and outcome examined. The identified reports primarily examined concurrent neurological severity and exploratory prediction of subsequent neurological status. These relationships should not be equated with recovery of independent walking, useful hand function, or everyday independence.

Existing biomarker combinations with imaging, preserved sensation, and clinical variables demonstrate partial integration. Nevertheless, high risk of bias in prediction models, incomplete confounding control, overlapping cohorts, and inconsistent follow-up limit transportability. Internally derived thresholds and apparent discrimination do not establish calibrated prediction, clinical utility, or a basis for selecting rehabilitation treatment.

Neuroendocrine findings address basal cortisol, stimulated adrenal responses and, in retained reports with age-eligibility uncertainty, androgen trajectories and functioning. They do not establish a causal link between psychological stress, hypothalamic–pituitary–adrenal activity, and neuroplasticity after injury. Similarly, circulating neurotrophic changes require independent structural or physiological evidence before being interpreted as neural reorganization.

For rehabilitation, the priority is to connect biological measurements with clearly defined clinical decisions and patient outcomes. Prospective studies should record achieved treatment exposure, distinguish assisted performance from persistent benefit, and evaluate whether biomarkers add information beyond neurological examination and imaging. Treatment selection requires direct evidence of differential benefit, while mechanistic claims require temporal and analytical designs.

Progress depends on reproducible assays, transparent reporting, independent validation, and handling of missing data and cohort dependence. The proposed framework identifies relationships that can be tested without assuming that all domains must be measured simultaneously. It supports focused translational research while preserving the distinction between a promising biological signal and a tool ready for clinical care.

## Figures and Tables

**Table 1:** Endocrine measures, chronic clinical associations and rehabilitation exposures organized by PICOS(T).

Report	P: population	I: Biomarker/Exposure	C: Comparison	O: Clinical Endpoint	S: Analysis Design	T: Assessment Time
Mechanick et al. (1997) [35]	49 SCI; 29 complete/20 incomplete	Cortisol, thyroid measures including free thyroxine index; PTH, vitamin D, prolactin, metabolic measures; Serum plus urine measures charted separately	Complete/incomplete SCI; 33 separate TBI comparators	Concurrent ASIA/endocrine measures	Retrospective	11–371 days; one assessment
Morse et al. (2008) [36]	63 chronic SCI men	High-sensitivity CRP; plasma	Motorized/manual wheelchair; assisted/independent walking	Concurrent mobility mode	Cross-sectional	21 ± 13 years; cross-sectional
Harness et al. (2014) [37]	15 chronic SCI; AIS A–C	BDNF, cortisol, prolactin; serum	Before/after one bout; motor-score and injury-duration associations	Acute biomarker response; motor-score association	Single-bout experiment	Chronic; one 2 h bout
Allison et al. (2016) [38]	20 chronic SCI; AIS A–D	CRP, IL-6; cytokine panel and eicosanoids; serum	Diet + supplements 12 vs. usual diet 8	Sensory neuropathic pain; inflammatory change	Randomized trial	Chronic; baseline/1/3 months
Goldstein et al. (2017) [39]	338 chronic SCI	High-sensitivity CRP; plasma	Motorized/manual wheelchair; assisted/independent walking	Concurrent mobility/adiposity	Cross-sectional	17.3 ± 13.3 years; cross-sectional
Cuce et al. (2019) [40]	22 SCI; 15 NP/7 without NP	Cortisol, ACTH, DHEAS, thyroid hormones; additional sex hormones and prolactin; serum	NP/non-NP; 10 healthy controls	Neuropathic pain and stimulated cortisol	Case-control physiology	Chronic; two ACTH challenges
Todd et al. (2021) [41]	8 analysed chronic SCI with NP	IL-6; IL-10, IL-1ra and TNF-α; plasma	Before/after one exercise bout; marker–pain-change correlation	Immediate/90 min pain and affect	Single-bout experiment	Chronic; immediate/90 min
Itodo et al. (2022) [18]	86 SCI; 70 men/16 women	DHEA, DHEAS; testosterone and SHBG; serum	Severity/level groups; endocrine trajectory during rehabilitation	Severity association; endocrine trajectory	Prospective cohort/case series	Entry median 15.5 days; discharge
Agulló-Ortuño et al. (2024) [42]	50 randomized; 40 biomarker-complete; subacute SCI; AIS C/D	IL-6, IL-8 and inflammatory panel; EDTA plasma	Early vs. late aquatic sequence; biomarker completers 21 vs. 19; usual therapy throughout	Cytokines; 10MWT and other function	Randomized sequence trial	Subacute; weeks 0/6/12
Seijas et al. (2025) [19]	80 traumatic/non-traumatic SCI; 65 men, 15 women; recruitment ≥ 16 years	Serum DHEA/DHEAS; free and total testosterone and SHBG	Baseline androgen concentrations and repeated functioning; sex-specific analyses	Rasch-transformed SCIM-III during rehabilitation and at discharge	Prospective cohort; prognostic-factor associations	Baseline blood median 42 days (IQR 30–54); median follow-up 167 days (128–224)

Abbreviations: SCI, spinal cord injury; TBI, traumatic brain injury; AIS, American Spinal Injury Association Impairment Scale; ASIA, American Spinal Injury Association; CRP, C-reactive protein; IL, interleukin; IL-1ra, interleukin-1 receptor antagonist; IL-6, interleukin-6; IL-8, interleukin-8; IL-10, interleukin-10; TNF-α, tumour necrosis factor alpha; PTH, parathyroid hormone; ACTH, adrenocorticotropic hormone; DHEA, dehydroepiandrosterone; DHEAS, dehydroepiandrosterone sulphate; IQR, interquartile range; SHBG, sex hormone-binding globulin; NP, neuropathic pain; 10MWT, 10-metre walk test; EDTA, ethylenediaminetetraacetic acid; PICOS(T), Population, Intervention or exposure, Comparator, Outcome, Study design and Timing; SCIM-III, Spinal Cord Independence Measure version III.

**Table 2:** Traumatic injury biomarkers and neurological outcomes organized by PICOS(T).

Report	P: population	I: Biomarker/Exposure	C: Comparison	O: Clinical Endpoint	S: Analysis Design	T: Assessment Time
Kwon et al. (2010) [33]	27 traumatic SCI; AIS A–C	IL-6, IL-8, GFAP, S100B, tau; BDNF/GDNF tested below detection; CSF and paired serum	AIS severity, motor recovery and pain; 12 non-SCI comparators	Initial AIS; 6-month motor gain/pain	Prospective cohort/case series	0–72 h; outcome 6 months
Hayakawa et al. (2012) [43]	14 traumatic cervical SCI	pNF-H; plasma	Serial AIS groups; complete versus incomplete injury	Serial/final AIS	Prospective cohort/case series	6 h–21 days; outcome 43–556 days
Pouw et al. (2014) [44]	16 traumatic SCI; AIS A–D	NfH, GFAP, S100B, NSE, tau; CSF	AIS severity and conversion subgroups	Initial AIS; later conversion	Prospective cohort/case series	≤24 h; outcome 6/12 months
Ungureanu et al. (2014) [45]	15 traumatic SCI; 8 complete/7 incomplete	pNF-H; CSF	Complete/incomplete SCI and serial AIS; 6 controls	Serial pNF-H and AIS trajectories	Prospective cohort/case series	6–12 h to ≤12 days; outcome 10–24 months
Ahadi et al. (2015) [17]	26 SCI among 35 trauma participants. Overall age range 16–64 years, without a separate SCI age range	GFAP, pNF-H, NSE; serum	Initial AIS severity and early survival; 9 fracture controls	Initial severity; early survival	Prospective cohort/case series	24/48/72 h; early survival
Kwon et al. (2017) [34]	50 traumatic SCI; AIS A–C	IL-6, IL-8, GFAP, S100B, tau; multiplex growth factors screened; CSF	AIS groups; converters/non-converters; motor-score change	Initial AIS; 6-month conversion/motor gain	Prospective cohort/case series	24–120 h; outcome 6 months
Singh et al. (2017) [46]	28 traumatic thoracolumbar SCI	pNF-H; plasma	Serial AIS severity groups; 12 fracture/AIS E controls	Concurrent/serial AIS	Prospective cohort/case series	Days 3/7/14/28; outcome day 90
Streijger et al. (2017) [47]	29 traumatic SCI; AIS A–C	165-target panel, including gamma-enolase/NSE and neural/immune proteins; CSF	AIS/outcome groups; pooled SCI positive control (6)	Severity; conversion; motor change	Prospective cohort/case series	24/48/72 h; outcome 6 months
Du et al. (2018) [48]	60 traumatic SCI; 21 improve/39 do not	NSE, S100B; serum	≥1-grade AIS improvers (21) vs. non-improvers (39)	6-month AIS improvement	Prospective cohort/case series	Admission–day 14; outcome 6 months
Rieder et al. (2019) [49]	52 isolated traumatic SCI	IL-6, GDNF, NGF, NSE; serum	Neurological/FIM associations; 36 healthy controls	6-month neurological/FIM outcomes	Prospective cohort/case series	48 h/day 7; outcome 6 months
Capirossi et al. (2020) [50]	9 analysed traumatic SCI	IL-6/IL-8, BDNF, NGF, NSE, total/phosphorylated tau and broad panel; CSF	Motor/sensory change and discharge independence correlations	Motor/sensory change; SCIM independence	Prospective cohort/case series	12–24 h; discharge 47–320 days
Stukas et al. (2023) [51]	118 traumatic SCI; AIS A–C	NfL, GFAP; serum and CSF	AIS conversion and motor gain; 19 non-SCI controls	Conversion; cervical motor gain	Prospective cohort/case series	24–96-h bins; outcome 6 months
Yang et al. (2023) [52]	15 traumatic SCI; AIS A/B	IL-6, GFAP, NfL, S100B, tau; UCH-L1/spectrin breakdown products; CSF and serum	AIS A/B and discharge improvement; 10 non-SCI controls	Initial AIS; discharge improvement	Prospective cohort/case series	Days 1–5; discharge < 3 months
Ding et al. (2024) [21]	374 traumatic cervical SCI	CRP plus cellular inflammatory measures; blood CRP (serum/plasma not specified)	One-year AIS A–C versus D–E; initial AIS/MRI prediction models	Initial severity; 1-year AIS	Retrospective	≤12 h blood/≤48 h MRI; 1 year
Haddadi et al. (2024) [53]	31 traumatic SCI; 9 complete/22 incomplete	S100B; serum and CSF	Complete (9) versus incomplete (22) SCI	Initial complete/incomplete injury	Prospective cohort/case series	24–48 h; no later endpoint
Azad et al. (2025) [54]	50 traumatic/compressive SCI	cfDNA plus IL-6, NfH, GFAP/NfL, FABP3 and REST; plasma	AIS severity and conversion; 20 non-SCI controls	Initial severity; 6-month AIS conversion	Prospective cohort/case series	≤7 days from deficit; outcome 6 months
Fraussen et al. (2025) [55]	37 traumatic SCI	NfL, GFAP, contactin-1; plasma (lithium heparin)	AIS conversion/≥5-point motor gain; 22 controls	Conversion; motor gain	Prospective cohort/case series	0–4 days to 18 weeks; variable endpoint
Raheja et al. (2025) [56]	26 traumatic SCI; 9 followed	GFAP, S100B, pNF-H, NSE; serum	Initial severity and 6-month AIS motor-status groups	Initial/later AIS status	Prospective cohort/case series	Admission/day 2/day 7; outcome 6 months
Waliullah et al. (2025) [57]	26 traumatic SCI	S100B; serum	Serial AIS severity groups; 26 controls	Concurrent/serial severity	Prospective cohort/case series	≤24 h/week 2/week 6
Hei et al. (2026) [58]	71 traumatic cervical SCI	GFAP, tau, contactin-1; serum, as reported in the collection and assay methods; conflicting plasma terminology in the title and results	AIS converters/non-converters; biomarker + MRI + AIS models	AIS conversion after discharge	Prospective cohort/case series	≤24 h admission; 6 months after discharge
Sheinberg et al. (2026) [59]	25 traumatic SCI; AIS A–C	Exosomal GFAP, NfH, tau, S100B, IL-6/IL-8, BDNF/NGF/GDNF, broader proteins and miRNAs; CSF exosome isolates; serum exosomal RNA comparator	Initial complete/incomplete SCI and later AIS improvement	Initial AIS; later AIS improvement	Prospective cohort/case series	Days 1/3/5; 3-month follow-up

Abbreviations: SCI, spinal cord injury; AIS, American Spinal Injury Association Impairment Scale; CSF, cerebrospinal fluid; CRP, C-reactive protein; IL, interleukin; IL-6, interleukin-6; IL-8, interleukin-8; BDNF, brain-derived neurotrophic factor; NGF, nerve growth factor; GDNF, glial cell line-derived neurotrophic factor; GFAP, glial fibrillary acidic protein; NSE, neuron-specific enolase; NfL, neurofilament light chain; NfH, neurofilament heavy chain; pNF-H, phosphorylated neurofilament heavy chain; S100B, S100 calcium-binding protein B; MRI, magnetic resonance imaging; SCIM, Spinal Cord Independence Measure; FIM, Functional Independence Measure; RNA, ribonucleic acid; PICOS(T), Population, Intervention or exposure, Comparator, Outcome, Study design and Timing; UCH-L1, ubiquitin C-terminal hydrolase L1; FABP3, fatty acid-binding protein 3; REST, RE1-silencing transcription factor.

**Table 3:** Myelopathy, exploratory inflammatory findings and distinct clinical contexts organized by PICOS(T).

Report	P: Population	I: Biomarker/Exposure	C: Comparison	O: Clinical Endpoint	S: Analysis Design	T: Assessment Time
Takahashi et al. (2014) [60]	14 analysed compressive myelopathy	pNF-H; CSF	Acute worsening versus chronic stable myelopathy; 37 controls	Myelopathy worsening; JOA recovery	Prospective cohort/case series	Preoperative; outcome 6 months
Kato et al. (2015) [61]	26 cervical compressive myelopathy	pNF-H; serum (methods also use the word plasma)	Myelopathy severity/recovery and recent worsening; 8 controls	mJOA severity; postoperative recovery	Prospective cohort/case series	Pre-/1-week postoperative; outcome 3 months
Holmström et al. (2020) [62]	12 acquired cord-tethering patients	GFAP, pNF-H; UCH-L1; CSF and plasma	Within-patient perioperative change and clinical correlations; 14 controls	AIS, EQ-5D, JOACMEQ; postoperative status	Prospective cohort/case series	Chronic deterioration; outcome 3 months
Lin et al. (2021) [63]	45 traumatic SCI; AIS A–D	IL-6, IL-8 plus cytokine/chemokine panel; CHRFAM7A genotype; EDTA plasma	CHRFAM7A genotype, AIS severity and pain groups over time	Inflammatory trajectory; neuropathic pain	Prospective cohort/case series	≤6 weeks; pain/concurrent measures
Wichmann et al. (2022) [64]	15 traumatic SCI; AIS A–D	IL-6, IL-8, GDNF and 92-protein inflammatory panel; CSF and EDTA plasma	AIS converters/non-converters; 15 controls	AIS conversion; motor/SCIM follow-up	Prospective cohort/case series	13 h/day 9/day 148; follow-up
Wichmann et al. (2023) [65]	Same 15 traumatic SCI	GFAP, NfL, NSE, IL-6/IL-8 plus cytokines; CSF, plasma; serum NSE excluded from analysis	Initial AIS/conversion and SCIM; 15 controls	Initial AIS; conversion; SCIM-III	Prospective cohort/case series	13 h/day 9/day 148; follow-up
Pu et al. (2024) [20]	60 combined SCI–TBI	NSE, S100B; neuritin (not neurofilament); serum-labelled specimens; methods also refer generically to serum/plasma	Favourable/poor/deceased GOS groups	Global GOS; combined SCI–TBI	Prospective cohort/case series	Sampling/outcome interval NR
Morrison et al. (2025) [66]	36 traumatic SCI; AIS A–D	CRP, IL-6, IL-8 and immune-mediator panel; plasma; whole-blood cellular/transcript assays separate	Motor-complete AIS A/B vs. C/D; 36 enrolled controls	Initial motor completeness	Cross-sectional	0–4 days; no later recovery
Vedantam et al. (2025) [67]	20 degenerative cervical myelopathy	NfL, GFAP, BDNF, tau, IL-6/IL-8 plus cytokines/amyloid; serum per processing methods	DCM versus 10 controls; presurgical marker–functional change	DCM diagnosis; pinch/functional change	Prospective cohort/case series	Chronic symptoms; pre-/3-month postoperative
Belogurov et al. (2026) [68]	8 analysed intramedullary-tumour patients	IL-6/IL-8 and cytokine/chemokine panel; Li-heparin plasma and CSF	Perioperative within-patient change and discharge-status correlations	Postoperative Karnofsky status	Prospective cohort/case series	0–168-h surgical series; discharge

Abbreviations: SCI, spinal cord injury; AIS, American Spinal Injury Association Impairment Scale; CSF, cerebrospinal fluid; IL, interleukin; IL-6, interleukin-6; IL-8, interleukin-8; BDNF, brain-derived neurotrophic factor; GDNF, glial cell line-derived neurotrophic factor; GFAP, glial fibrillary acidic protein; NSE, neuron-specific enolase; NfL, neurofilament light chain; pNF-H, phosphorylated neurofilament heavy chain; S100B, S100 calcium-binding protein B; SCIM, Spinal Cord Independence Measure; JOA, Japanese Orthopaedic Association score; JOACMEQ, Japanese Orthopaedic Association Cervical Myelopathy Evaluation Questionnaire; EQ-5D, EuroQol five-dimension instrument; GOS, Glasgow Outcome Scale; EDTA, ethylenediaminetetraacetic acid; PICOS(T), Population, Intervention or exposure, Comparator, Outcome, Study design and Timing; CHRFAM7A, CHRNA7–FAM7A fusion gene; UCH-L1, ubiquitin C-terminal hydrolase L1; SCIM-III, Spinal Cord Independence Measure version III.

## Data Availability

The registered protocol and its amendments are available under DOI 10.17605/OSF.IO/M3JDT. Database-specific search strategies, selection counts, study characteristics, extracted findings, and critical appraisals are provided in the Supplementary Materials. Additional review records are available from the corresponding author on reasonable request.

## Supplementary Materials

The following supporting information can be downloaded at: https://www.mdpi.com/article/10.3390/ijms27219610/s1.
