# Supplementary material for: From Molecular State to Clinical Outcomes After Spinal Cord Injury: A Scoping Review of Selected Fluid Biomarkers and Rehabilitation Relevance
Source: Int J Mol Sci. 2026 Oct 27;27(21):9610. doi: 10.3390/ijms27219610 (PMC13762652; doi:10.3390/ijms27219610)
Supplement: Supplementary file 1 [file ijms-27-09610-s001.zip › ijms-4580160-supplementary.pdf]

Table S1. Review design, protocol registration and amendments		
**Item**	**Specification**	**Interpretive boundary**
Design	Scoping review of selected human fluid biomarkers and clinical relationships after SCI.	Descriptive mapping; no common effect estimate or clinical treatment algorithm.
Original registration	OSF DOI 10.17605/OSF.IO/M3JDT; registration 4 July 2026 and initial search 6 July 2026.	OSF registration is separate from PRISMA-ScR reporting.
Amendment registration	Protocol amendments decided and registered on OSF on 7 October 2026 under DOI 10.17605/OSF.IO/M3JDT.	Amendments preceded the updated screening but followed an earlier evidence map and examination of its literature.
Focused search	Updated database searches conducted on 8 October 2026. Screening of the updated records began on the same day.	The search scope was amended after the original registration and before the updated screening.
Population and clinical phenotypes	Traumatic SCI, non-traumatic cord injury, compressive/degenerative myelopathy, acquired tethering and perioperative cord injury described separately.	Acute surgical sampling does not make the entire preceding chronic disease course acute.
Age-related eligibility exceptions	Ahadi: overall trauma-cohort age range 16–64 years, without a separate SCI range. Itodo: recruitment permitted >16 years. Seijas: recruitment permitted ≥16 years. Adult-only estimates are not separately reported.	These reports are retained as explicit age-related exceptions. Their adult-only applicability cannot be established from the reported eligibility or age information.
Combined neurotrauma	Pu: all 60 participants have SCI plus TBI.	Global GOS cannot isolate spinal recovery.
Unresolved specimen label	Ding: blood CRP; serum/plasma and platform not specified.	The unspecified matrix limits comparison with matrix-specific estimates.
Panel-level relationship rule	A target biomarker assessed in a qualifying panel plus a tested/described eligible clinical relationship; null tests retained.	Panel inclusion does not establish a relationship for every constituent; target analytes and model drivers are distinguished.
Selection counts	17,317 identified records; 640 duplicates; 16,677 screened; 183 reports sought; 8 not retrieved; 175 assessed; 134 excluded; 41 included.	Reports are distinguished from records; unavailable reports and full-text exclusions are recorded separately. Reports do not establish independent cohorts.
Human/preclinical boundary	Belogurov and Sheinberg: separately identifiable human measurements only.	Rodent drug effects and in vitro experiments do not establish human treatment benefit or plasticity.
Reporting	PRISMA-ScR, with PRISMA 2020 flow and PRISMA-S search principles.	Protocol registration and reporting guidelines serve distinct purposes.

Table S1. Review design, protocol registration and amendments

Item	Specification	Interpretive boundary
Post-registration critical appraisal	Tool-specific appraisal was introduced after protocol registration. The included reports comprise two randomized intervention reports and 39 nonrandomized reports: 14 QUIPS, 10 PROBAST+AI and 15 JBI. Tables S10c–S10k provide domain/item assessments.	Result-specific judgments, supporting reasons and instrument versions are reported in Tables S10b–S10l.
Scope and eligibility changes	Broad stress, autonomic, imaging, neurophysiological and intervention scope refocused on selected serum, plasma or CSF targets linked to defined clinical relationships. Adult-focused and English-language eligibility specified.	The exact current target list and mandatory fluid-matrix requirement were not fully specified in the original protocol.
Search reformulation	Earlier human-participant block removed. Selected molecular targets and an explicit matrix block added, with revised clinical-concept vocabulary and interface-specific all-fields scope.	The original protocol and earlier supplementary strings differed. The updated search combines extensions and restrictions rather than demonstrating greater sensitivity.
Charting and synthesis changes	Timing, relationship type, analytical denominators, cohort dependence and rehabilitation exposure explicitly charted. Structured tool-specific appraisal supplemented descriptive methodological charting.	Clinical questions and outcomes were synthesized separately. No pooled effect or cross-design certainty grade was calculated.

Abbreviations: SCI, spinal cord injury; TBI, traumatic brain injury; CRP, C-reactive protein; GOS, Glasgow Outcome Scale; OSF, Open Science Framework; PRISMA-ScR, Preferred Reporting Items for Systematic Reviews and Meta-Analyses extension for Scoping Reviews; PRISMA-S, Preferred Reporting Items for Systematic Reviews and Meta-Analyses extension for Reporting Literature Searches; PRISMA, Preferred Reporting Items for Systematic Reviews and Meta-Analyses; JBI, JBI critical-appraisal instruments; QUIPS, Quality in Prognosis Studies; DOI, digital object identifier; PRESS, Peer Review of Electronic Search Strategies; PROBAST+AI, Prediction model Risk Of Bias ASsessment Tool with updated provisions for regression and artificial intelligence methods.

Table S2. PICOS(T) framework

Component	Operational specification	Boundary
Population	Adult-focused traumatic or non-traumatic cord injury; any level, severity or phase. Myelopathy, tethering and perioperative injury are separately described.	Age-related exceptions in Ahadi/Itodo/Seijas and combined SCI–TBI Pu are explicit retained exceptions (S1).
Intervention or exposure	Target biomarker concentration, trajectory or stimulated response in serum, plasma or CSF; intervention exposure in otherwise eligible reports.	Imaging, rehabilitation or stimulation without an eligible fluid relationship is insufficient.
Comparator	Severity/pain/outcome groups; healthy/disease comparators; within-person comparison; continuous association.	A separate control arm is not required; SCI–control diagnosis alone is insufficient without an eligible within-SCI relationship.
Outcome	Initial neurological severity, neuropathic pain, neurological change, task performance or everyday independence; independently assessed neuroplasticity.	Baseline severity, future status, conversion, motor change and independence are non-equivalent outcomes.
Study design	Original trials, cohorts, case-control/cross-sectional analyses, physiological experiments and case series.	Registry-only, review, protocol, single-case, preclinical-only and tissue-only reports excluded.
Timing	All injury phases; exact recruitment, sampling, intervention and outcome intervals charted separately.	Acute ≤14 days; subacute >14 days–6 months; chronic >6 months; mixed/unclassified when needed.

Abbreviations: SCI, spinal cord injury; TBI, traumatic brain injury; CSF, cerebrospinal fluid; PICOS(T), Population, Intervention or exposure, Comparator, Outcome, Study design and Timing.

Table S3. Eligibility criteria and documented exceptions

Criterion	Included	Excluded or qualified
Publication	Peer-reviewed original English full text.	Abstract-only, review, protocol, registry-only and unreviewed preprint.
Population	Adult-focused SCI and specified non-traumatic cord phenotypes.	Age <18 or combined diagnoses require separation; Ahadi/Itodo/Seijas/Pu retained as explicit exceptions.
Matrix	Serum, plasma or CSF; isolated CSF exosomes identified separately.	Tissue, saliva, urine or cellular whole-blood assays alone. Ding blood CRP matrix unresolved.
Neuroendocrine targets	Cortisol, ACTH, DHEA/DHEAS, thyroid hormones.	TSH, testosterone/PTH alone do not independently qualify; other measures charted within eligible reports.
Inflammatory targets	CRP, IL-6, IL-8.	Other cytokines may be charted in qualifying panels; membership does not establish target-specific association.
Neurotrophic targets	BDNF, NGF, GDNF.	Assayed but below-detection factors do not provide quantitative clinical evidence.
Neural injury targets	GFAP, NfL/NfH/pNF-H, S100B, NSE, tau and contactin-1.	Neuritin is not a neurofilament; Pu qualifies through NSE.
Relationship	Concurrent severity/pain comparison, correlation/regression, prognosis, serial clinical relationship or intervention analysis.	Mere biomarker/outcome co-presence without an examined clinical relationship.
Neuroplasticity	Independent assessment of neural reorganization or adaptation.	Biomarker increase or task improvement alone.
Significance	Positive, discordant and null tests retained.	No exclusion for a nonsignificant result.
HPA requirement	At least one eligible biomarker domain.	Absence of HPA measurements is never an exclusion reason.
Preclinical components	Separable human data in mixed translational reports.	Drug-related locomotor benefit in rodents is not human treatment efficacy.

Abbreviations: SCI, spinal cord injury; CSF, cerebrospinal fluid; CRP, C-reactive protein; IL, interleukin; IL-6, interleukin-6; IL-8, interleukin-8; BDNF, brain-derived neurotrophic factor; NGF, nerve growth factor; GDNF, glial cell line-derived neurotrophic factor; GFAP, glial fibrillary acidic protein; NSE, neuron-specific enolase; NfL, neurofilament light chain; NfH, neurofilament heavy chain; pNF-H, phosphorylated neurofilament heavy chain; S100B, S100 calcium-binding protein B; PTH, parathyroid hormone; ACTH, adrenocorticotropic hormone; HPA, hypothalamic–pituitary–adrenal; DHEA, dehydroepiandrosterone; DHEAS, dehydroepiandrosterone sulfate; TSH, thyroid-stimulating hormone.

Table S4a. Database interfaces, fields, and retrieval settings		
**Source**	**Search date and interface scope**	**Retrieval filters and coverage**
PubMed	8 October 2026; untagged expression. Unquoted terms subject to PubMed automatic term mapping; quoted phrases handled through its phrase index.	No additional date, language, age, human-only or study-design filters. All-fields scope was adapted to the interface. English-language eligibility was applied during screening.
Web of Science	8 October 2026; search; ALL= field expressions.	No additional date, language, age, human-only or study-design filters. All-fields scope was adapted to the interface. English-language eligibility was applied during screening.
Embase	8 October 2026; search; all-fields interface with quoted terms.	No additional date, language, age, human-only or study-design filters. All-fields scope was adapted to the interface. English-language eligibility was applied during screening.
Cochrane Library	8 October 2026; search; interface all-text scope (the corresponding all-fields search), including CENTRAL, review and protocol outputs.	No additional date, language, age, human-only or study-design filters. All-fields scope was adapted to the interface. English-language eligibility was applied during screening.
Scopus	8 October 2026; all-fields query builder, including titles, abstracts, keywords and reference metadata; no TITLE-ABS-KEY restriction.	No additional date, language, age, human-only or study-design filters. All-fields scope was adapted to the interface. English-language eligibility was applied during screening.
Coverage constraint	SCI AND named biomarkers AND matrix AND clinical outcome/plasticity terms. Relevant information absent from searchable metadata can prevent retrieval. All-fields scope does not establish complete coverage.	Mandatory blocks and database-specific indexing or term mapping can reduce recall. The October reformulation does not establish retrieval sensitivity.

Abbreviations: SCI, spinal cord injury; CENTRAL, Cochrane Central Register of Controlled Trials; AND, Boolean AND operator; ALL, all-fields search scope.

Table S4b. Database retrieval counts and metadata completeness

Source	Records	Titles	Abstracts or summaries	DOI	PMID
PubMed	96	96	NR	92	96
Web of Science	270	270	269	261	212
Embase literature	495	495	489	470	309
CENTRAL	15	15	15	9	7
Cochrane review	1	1	1	1	NA
Cochrane protocol	1	1	1	1	NA
Scopus	16418	16417	16140	15976	11187
Embase registrations	21	21	21	NA	NA
Total records	17317	NA	NA	NA	NA

Note: Embase total 516 = 495 literature + 21 registration records. Cochrane total 17 = 15 CENTRAL + 1 review + 1 protocol. Six CENTRAL records are registration records already counted within 15. The reported companion PubMed text output adds no records. Counts describe retrieval, not eligible human studies; record types do not determine full-text eligibility. Unequal database yields cannot be interpreted as differences in eligible evidence. Scopus all-fields coverage and PubMed term processing differ; the numerical contribution of these differences was not measured.

Abbreviations: NR, not reported; NA, not applicable; DOI, digital object identifier; PMID, PubMed identifier; CENTRAL, Cochrane Central Register of Controlled Trials.

Table S5. Database-specific search strategies	
**Database and field scope**	**Expression**
PubMed; untagged all fields	("spinal cord injury" OR "spinal cord injuries") AND (cortisol OR ACTH OR DHEA OR DHEAS OR "HPA axis" OR "thyroid hormone" OR "thyroid hormones" OR "C reactive protein" OR "interleukin 6" OR "interleukin 8" OR BDNF OR NGF OR GDNF OR GFAP OR "neurofilament light" OR "neurofilament heavy" OR S100B OR "neuron specific enolase" OR "tau protein" OR "contactin 1") AND (serum OR plasma OR "cerebrospinal fluid" OR CSF) AND ("injury severity" OR "neurological severity" OR "neurological recovery" OR "motor recovery" OR "functional recovery" OR "neurological outcome" OR "neurological outcomes" OR "neuropathic pain" OR neuroplasticity OR "neural plasticity")
Web of Science; ALL=	(((ALL=(("spinal cord injury" OR "spinal cord injuries"))) AND ALL=((cortisol OR ACTH OR DHEA OR DHEAS OR "HPA axis" OR "thyroid hormone" OR "thyroid hormones" OR "thyroid function" OR "C reactive protein" OR "interleukin 6" OR "interleukin 8" OR BDNF OR NGF OR GDNF OR GFAP OR neurofilament OR neurofilaments OR S100B OR "neuron specific enolase" OR "tau protein" OR "contactin 1"))) AND ALL=((blood OR serum OR plasma OR "cerebrospinal fluid" OR CSF))) AND ALL=(("injury severity" OR "neurological severity" OR "neurological recovery" OR "motor recovery" OR "functional recovery" OR "neurological outcome" OR "neurological outcomes" OR "neuropathic pain" OR neuroplasticity OR "neural plasticity"))
Embase; all-fields interface	('spinal cord injury' OR 'spinal cord injuries') AND ('cortisol' OR 'acth' OR 'dhea' OR 'dheas' OR 'hpa axis' OR 'thyroid hormone' OR 'thyroid hormones' OR 'thyroid function' OR 'c reactive protein' OR 'interleukin 6' OR 'interleukin 8' OR 'bdnf' OR 'ngf' OR 'gdnf' OR 'gfap' OR 'neurofilament' OR 'neurofilaments' OR 's100b' OR 'neuron specific enolase' OR 'tau protein' OR 'contactin 1') AND ('blood' OR 'serum' OR 'plasma' OR 'cerebrospinal fluid' OR 'csf') AND ('injury severity' OR 'neurological severity' OR 'neurological recovery' OR 'motor recovery' OR 'functional recovery' OR 'neurological outcome' OR 'neurological outcomes' OR 'neuropathic pain' OR 'neuroplasticity' OR 'neural plasticity')
Cochrane Library; all text	("spinal cord injury" OR "spinal cord injuries") AND (cortisol OR ACTH OR DHEA OR DHEAS OR "HPA axis" OR "thyroid hormone" OR "thyroid hormones" OR "C reactive protein" OR "interleukin 6" OR "interleukin 8" OR BDNF OR NGF OR GDNF OR GFAP OR "neurofilament light" OR "neurofilament heavy" OR S100B OR "neuron specific enolase" OR "tau protein" OR "contactin 1") AND (serum OR plasma OR "cerebrospinal fluid" OR CSF) AND ("injury severity" OR "neurological severity" OR "neurological recovery" OR "motor recovery" OR "functional recovery" OR "neurological outcome" OR "neurological outcomes" OR "neuropathic pain" OR neuroplasticity OR "neural plasticity")
Scopus; all-fields query builder	("spinal cord injury" OR "spinal cord injuries") AND (cortisol OR ACTH OR DHEA OR DHEAS OR "HPA axis" OR "thyroid hormone" OR "thyroid hormones" OR "C reactive protein" OR "interleukin 6" OR "interleukin 8" OR BDNF OR NGF OR GDNF OR GFAP OR "neurofilament light" OR "neurofilament heavy" OR S100B OR "neuron specific enolase" OR "tau protein" OR "contactin 1") AND (serum OR plasma OR "cerebrospinal fluid" OR CSF) AND ("injury severity" OR "neurological severity" OR "neurological recovery" OR "motor recovery" OR "functional recovery" OR "neurological outcome" OR "neurological outcomes" OR "neuropathic pain" OR neuroplasticity OR "neural plasticity")

Note: Searches were conducted on 8 October 2026 without date, language, age, human-only, or study-design filters. The English-language restriction applied at eligibility assessment. Database expressions retain the executed field syntax and platform-specific vocabulary. Mandatory biomarker, matrix, and clinical-concept blocks delimit retrieval; record counts are reported in Table S4b. The Scopus expression contains four parenthesized OR blocks connected by AND, with 2 SCI terms, 20 biomarker terms, 4 matrix terms and 10 clinical-concept terms. Its structure is retained exactly as supplied. The updated search combined changes in scope, vocabulary and fields and is not reported as a validated sensitivity comparison.

Abbreviations: CSF, cerebrospinal fluid; BDNF, brain-derived neurotrophic factor; NGF, nerve growth factor; GDNF, glial cell line-derived neurotrophic factor; GFAP, glial fibrillary acidic protein; S100B, S100 calcium-binding protein B; OR, Boolean OR operator; ACTH, adrenocorticotropic hormone; HPA, hypothalamic–pituitary–adrenal; DHEA, dehydroepiandrosterone; DHEAS, dehydroepiandrosterone sulfate; AND, Boolean AND operator; ALL, all-fields search scope.

Table S6a. Clinical profiles and analytical designs of the 41 reports

Report and setting	Population and analysed SCI sample	Comparator and design
Mechanick et al. (1997) [35]; USA; Mount Sinai	SCI, 29 complete and 20 incomplete; separate TBI comparator group; metabolic-bone evaluation selected sample; SCI sample: 49	Complete/incomplete SCI; 33 separate TBI comparators; Retrospective cross-sectional medical-record analysis
Morse et al. (2008) [36]; USA; VA Boston	82 men provided blood samples; 64 selected by mobility mode and one CRP outlier excluded; 63 analysed; chronic SCI and no statin use; All men; mean age 55.5±14.3 years. SCI sample: 63	Motorized/manual wheelchair; assisted/independent walking; Cross-sectional cohort analysis
Kwon et al. (2010) [33]; Canada; Vancouver	AIS A 14, B 7, C 6; 21 cervical and 6 thoracic; selected reliable neurological examination; SCI sample: 27	AIS severity, motor recovery and pain; 12 non-SCI comparators; Prospective biomarker cohort nested in CSF-drainage trial
Hayakawa et al. (2012) [43]; Japan; Tokyo	Traumatic cervical SCI; age 30–88; concurrent intracranial haemorrhage excluded; SCI sample: 14	Serial AIS groups; complete versus incomplete injury; Prospective serial pilot cohort (author-labelled cross-sectional)
Harness et al. (2014) [37]; USA; California	12 men, 3 women with chronic SCI, AIS A–C; age 31.8±10.9 years; SCI sample: 15	Before/after one bout; motor-score and injury-duration associations; Uncontrolled acute exercise physiology experiment
Pouw et al. (2014) [44]; Netherlands/Canada; Nijmegen/Vancouver	23 screened, 16 included; AIS A 7, B 2, C 4, D 3; traumatic C2–T12; age 18–84; SCI sample: 16	AIS severity and conversion subgroups; Prospective two-centre cohort
Takahashi et al. (2014) [60]; Japan; Sakura	8 acutely worsening and 6 chronic myelopathy, plus 37 lumbar-stenosis controls; abstract/methods initially refer to 15 myelopathy patients; SCI sample: 14	Acute worsening versus chronic stable myelopathy; 37 controls; Prospective compressive-myelopathy cohort
Ungureanu et al. (2014) [45]; Romania; Iaşi	8 complete and 7 incomplete cervical/thoracic SCI; ages 21–59; 14 operated and one early death; SCI sample: 15	Complete/incomplete SCI and serial AIS; 6 controls; Prospective serial-sampling case series
Ahadi et al. (2015) [17]; Iran	26 SCI among 35 trauma patients. AIS A 10, B 7, C/D 9. The overall 16–64-year range includes the nine trauma controls, without a separate SCI age range or adult-only estimates. SCI sample: 26	Initial AIS severity and early survival; 9 fracture controls; Prospective acute cohort with fracture controls
Kato et al. (2015) [61]; Japan; Tokyo	Cervical compressive myelopathy; adults 38–86; idiopathic-scoliosis controls age 11–27; SCI sample: 26	Myelopathy severity/recovery and recent worsening; 8 controls; Prospective perioperative cohort
Allison et al. (2016) [38]; Canada; Brock University	Chronic SCI, AIS A–D, C2–L4, 10 women and 10 men; 12 intervention and 8 control; SCI sample: 20	Diet+supplements 12 vs usual diet 8; Randomized parallel-group trial
Goldstein et al. (2017) [39]; USA; VA Boston	360 recruited, 338 analysed; chronic SCI of any aetiology; age 54.1±14.4 years; non-ventilator-dependent; 278 men and 60 women. SCI sample: 338	Motorized/manual wheelchair; assisted/independent walking; Cross-sectional cohort analysis
Kwon et al. (2017) [34]; Canada; Vancouver	32 cervical and 18 thoracic; AIS A–C; 48/50 six-month follow-up; SCI sample: 50	AIS groups; converters/non-converters; motor-score change; Prospective biomarker cohort
Singh et al. (2017) [46]; India; Lucknow	Conservatively managed/refused surgery T4–L2 traumatic SCI; AIS A 9, B 12, C 7; fracture AIS E controls; 18 men and 10 women. SCI sample: 28	Serial AIS severity groups; 12 fracture/AIS E controls; Prospective serial pilot cohort
Streijger et al. (2017) [47]; Canada; multicentre SCI programme	AIS A 10, B 12, C 7; 27 clinical follow-up; separate pooled six-patient SCI positive control; SCI sample: 29	AIS/outcome groups; pooled SCI positive control (6); Prospective targeted-proteomics cohort

Table S6a. Clinical profiles and analytical designs of the 41 reports

Report and setting	Population and analysed SCI sample	Comparator and design
Du et al. (2018) [48]; China; Tianjin	21 ≥1-grade AIS improvers and 39 with <1-grade change at 6 months; adults 18–65; traumatic SCI; Improvers: 17 men/4 women, age 40.6±11.2 years; non-improvers: 29 men/10 women, age 42.6±13.4 years. SCI sample: 60	≥1-grade AIS improvers (21) vs non-improvers (39); Prospective serial acute cohort
Cuce et al. (2019) [40]; Türkiye; Erciyes University	Chronic traumatic SCI, 15 with neuropathic pain and 7 without pain; SCI sample: 22	NP/non-NP; 10 healthy controls; Case-control physiological study
Rieder et al. (2019) [49]; Brazil	Isolated traumatic SCI in ICU; 90% men; mean age 35.08±14.54; no head/multiple trauma; SCI sample: 52	Neurological/FIM associations; 36 healthy controls; Prospective acute cohort
Capirossi et al. (2020) [50]; Italy; Montecatone	11 recruited; one blood-contaminated CSF exclusion and one death; 9 analysed; cervical/thoracic traumatic SCI, ages 35–74; SCI sample: 9	Motor/sensory change and discharge independence correlations; Prospective rehabilitation pilot cohort
Holmström et al. (2020) [62]; Sweden; Uppsala	Chronic acquired cord tethering; six traumatic SCI and six other aetiologies; seven tumour and seven radiculopathy controls; SCI sample: 12	Within-patient perioperative change and clinical correlations; 14 controls; Prospective symptomatic-tethering surgical cohort
Lin et al. (2021) [63]; USA; University of Pittsburgh	25 AIS A/B and 20 AIS C/D traumatic SCI; 27 CHRFAM7A Δ2bp carriers; age 18–67; AIS A/B group: 21 men/4 women, age 35.74±15.78 years; AIS C/D group: 17 men/3 women, age 47.62±13.95 years. SCI sample: 45	CHRFAM7A genotype, AIS severity and pain groups over time; Prospective serial inflammatory/genetic cohort
Todd et al. (2021) [41]; Canada	10 enrolled, 8 analysed (4 tetraplegia, 4 paraplegia), AIS A–C, already physically active; chronic neuropathic pain; SCI sample: 8	Before/after one exercise bout; marker–pain-change correlation; Uncontrolled single-bout physiological experiment
Itodo et al. (2022) [18]; Switzerland; SwiSCI	70 men and 16 women; 61 traumatic and 25 non-traumatic SCI; initial inpatient rehabilitation; SwiSCI enrolment permitted age >16 years, adult-only estimates not separated; Median age 51 years (IQR 36–64). SCI sample: 86	Severity/level groups; endocrine trajectory during rehabilitation; Prospective longitudinal cohort
Wichmann et al. (2022) [64]; Denmark; Aarhus	Traumatic SCI, AIS A–D; 11 complete follow-up, 4 conversions; age 21–75; SCI sample: 15	AIS converters/non-converters; 15 controls; Prospective serial proteomics pilot cohort
Stukas et al. (2023) [51]; Canada/USA; multicentre	AIS A 78, B 20, C 20; 68 cervical/50 thoracolumbar; 113/118 with 6-month examination; 94 men and 24 women; mean age 42.9±17.0 years. SCI sample: 118	AIS conversion and motor gain; 19 non-SCI controls; Secondary prospective biospecimen-cohort analysis
Wichmann et al. (2023) [65]; Denmark; Aarhus	Same 15 traumatic SCI; 11 follow-up; four AIS improvements; SCI sample: 15	Initial AIS/conversion and SCIM; 15 controls; Prospective serial structural/cytokine pilot cohort
Yang et al. (2023) [52]; USA	AIS A 9 and B 6; four AIS improvements at discharge; clinically indicated traumatic-durotomy drain; SCI sample: 15	AIS A/B and discharge improvement; 10 non-SCI controls; Prospective acute biospecimen cohort
Agulló-Ortuño et al. (2024) [42]; Spain; Toledo	50 enrolled/randomized, 40 complete biomarker data: 21 early and 19 late aquatic sequence; 20 traumatic/20 non-traumatic, AIS C/D; SCI sample: 40	Early aquatic 21 vs late aquatic 19; usual therapy throughout; Randomized two-sequence aquatic-therapy trial
Ding et al. (2024) [21]; China; Nanchang	Acute traumatic cervical SCI; analysed ages 23–84; AIS A 124, B 45, C 50, D 155; 313 men and 61 women; mean age 54.67±12.28 years. SCI sample: 374	One-year AIS A–C versus D–E; initial AIS/MRI prediction models; Retrospective two-centre prognostic cohort
Haddadi et al. (2024) [53]; Iran; Sari	31 traumatic SCI; 9 complete and 22 incomplete; adults; major co-injury excluded; SCI sample: 31	Complete (9) versus incomplete (22) SCI; Acute diagnostic/severity cohort

Table S6a. Clinical profiles and analytical designs of the 41 reports		
**Report and setting**	**Population and analysed SCI sample**	**Comparator and design**
Pu et al. (2024) [20]; China; Shanghai	All participants have SCI with concomitant TBI; 42 favourable, 11 poor and 7 deceased; age 20–69; SCI sample: 60	Favourable/poor/deceased GOS groups; Prospective combined-neurotrauma cohort
Azad et al. (2025) [54]; USA; two centres	14 traumatic SCI, 19 traumatic central cord syndrome, 17 acute epidural compression; proteomic/6-month model n=34; SCI sample: 50	AIS severity and conversion; 20 non-SCI controls; Prospective two-centre multi-analyte cohort
Fraussen et al. (2025) [55]; Belgium/Netherlands	Traumatic SCI; initial AIS available 37/37; outcome 34. Reported conversion groups total 33 and motor-change groups total 29. Model-specific complete-case ROC n not stated. SCI sample: 37	AIS conversion/≥5-point motor gain; 22 controls; Prospective longitudinal multicentre cohort
Morrison et al. (2025) [66]; USA/Canada; multicentre	Traumatic SCI; AIS A/B 18, C/D 18; ages 21–85; many severe accompanying injuries; 35 SCI and 16 control ELISA samples; 27 men and 9 women; USA/Canada multicentre recruitment in 2016–2020. SCI sample: 36	Motor-complete AIS A/B vs C/D; 36 enrolled controls; Acute cross-sectional immune-phenotyping cohort
Raheja et al. (2025) [56]; India; New Delhi	Traumatic SCI, age 18–65; 16 AIS A; excludes head injury/polytrauma; SCI sample: 26	Initial severity and 6-month AIS motor-status groups; Prospective acute cohort
Seijas et al. (2025) [19]; Switzerland; four SwiSCI rehabilitation centres	80 SCI; 65 men/15 women; 58 traumatic/22 non-traumatic; median age 51 (IQR 36–64); recruitment ≥16 years; adult-only estimates unavailable	Baseline androgen groups/continuous concentrations; repeated Rasch-transformed SCIM-III and discharge functioning; prospective cohort
Vedantam et al. (2025) [67]; USA; Wisconsin	Adults with degenerative cervical myelopathy scheduled for decompression; median age 70, controls 65; 10 men and 10 women; age range 37–78 years. SCI sample: 20	DCM versus 10 controls; presurgical marker–functional change; Prospective perioperative degenerative-myelopathy cohort
Waliullah et al. (2025) [57]; India; Lucknow	Acute traumatic SCI, 15 complete paraplegia and 11 incomplete injury/paraparesis; age 18–60; no steroids; SCI sample: 26	Serial AIS severity groups; 26 controls; Prospective case-control serial cohort
Belogurov et al. (2026) [68]; Russia; Moscow	10 intramedullary-tumour patients enrolled, 8 ependymoma patients analysed; age 28–60; animal treatment results charted separately; SCI sample: 8	Perioperative within-patient change and discharge-status correlations; Prospective perioperative human cohort within a translational study
Hei et al. (2026) [58]; China; Nanchang	Acute cervical traumatic SCI; age 26–88; surgical patients; 54 men and 17 women; mean age 58.66±12.44 years. SCI sample: 71	AIS converters/non-converters; biomarker+MRI+AIS models; Prospective single-centre prognostic cohort
Sheinberg et al. (2026) [59]; USA	Acute traumatic SCI AIS A 11, B 7, C 7; 21 cervical; 38 quality-controlled CSF RNA specimens, not 38 patients; SCI sample: 25	Initial complete/incomplete SCI and later AIS improvement; Prospective acute exosome-profiling cohort
Note: Numbers refer to the report’s principal human sample; smaller endpoint/model denominators are specified in Table S9. External comparators are not added to the SCI sample. Recruitment dates are specified in Table S6c when relevant to cohort dependence; no independent total number of participants is calculated.		

Abbreviations: SCI, spinal cord injury; TBI, traumatic brain injury; AIS, American Spinal Injury Association Impairment Scale; CSF, cerebrospinal fluid; CRP, C-reactive protein; MRI, magnetic resonance imaging; SCIM, Spinal Cord Independence Measure; FIM, Functional Independence Measure; GOS, Glasgow Outcome Scale; NP, neuropathic pain; IQR, interquartile range; ELISA, enzyme-linked immunosorbent assay; CHRFAM7A, CHRNA7–FAM7A fusion gene; DCM, degenerative cervical myelopathy; ICU, intensive care unit; VA, Veterans Affairs; USA, United States of America.

Table S6b. Biological matrices, assays and specimen handling		
**Report**	**Target and additional biomarkers; matrix**	**Assay and handling**
Mechanick et al. (1997) [35]	Cortisol, thyroid measures including free thyroxine index; PTH, vitamin D, prolactin, metabolic measures; Serum plus urine measures charted separately	Clinical laboratory; PTH chemiluminescent immunometric assay; Single same-morning serum collection; 24 h urine separate
Morse et al. (2008) [36]	High-sensitivity CRP; Plasma	Immunoturbidimetry; EDTA blood shipped cold overnight, plasma −80 °C; hsCRP detection limit 0.03 mg/L
Kwon et al. (2010) [33]	IL-6, IL-8, GFAP, S100B, tau; BDNF/GDNF tested below detection; CSF and paired serum	Bio-Plex multiplex; BioVendor/Invitrogen ELISAs; CSF 1000rcf/10 min; serum clot 15 min; −80 °C
Hayakawa et al. (2012) [43]	pNF-H; Plasma	BioVendor ELISA; effective detection limit 70 pg/mL; Duplicate assays; centrifuged, frozen; storage temperature not specified
Harness et al. (2014) [37]	BDNF, cortisol, prolactin; Serum	Multiplex bead ELISA, Milliplex/Luminex; 12:00–16:00, 2-hour fast, no exercise for24 h; baseline and after each activity
Pouw et al. (2014) [44]	NfH, GFAP, S100B, NSE, tau; CSF	Sandwich ELISA; Liaison NSE/S100B, homemade GFAP, Innotest tau; 3000 rpm/5 min, −80 °C; one CSF insufficient for NfH
Takahashi et al. (2014) [60]	pNF-H; CSF	BioVendor ELISA; See reported injury-to-sampling window
Ungureanu et al. (2014) [45]	pNF-H; CSF	Millipore sandwich ELISA; Serial lumbar puncture; frozen until assay, temperature not specified
Ahadi et al. (2015) [17]	GFAP, pNF-H, NSE; Serum	Abnova/Millipore/Eagle ELISAs; Centrifugation 2000 rpm, 5–8 min; −20 then −80 °C
Kato et al. (2015) [61]	pNF-H; Serum (methods also use the word plasma)	BioVendor ELISA; cut-off 70 pg/mL; See reported injury-to-sampling window
Allison et al. (2016) [38]	CRP, IL-6; cytokine panel and eicosanoids; Serum	MAGPIX/Luminex; CRP/PGE2/LTB4 ELISA; 13:00 at baseline, 1 and 3 months; clot 30 min, 1000g/15 min, −80 °C
Goldstein et al. (2017) [39]	High-sensitivity CRP; Plasma	Immunoturbidimetry; EDTA plasma, centrifuged and stored −80 °C; hsCRP detection limit 0.03 mg/L
Kwon et al. (2017) [34]	IL-6, IL-8, GFAP, S100B, tau; multiplex growth factors screened; CSF	Bio-Plex custom 5-plex plus ELISAs; Duplicate ELISAs, −80 °C; growth factors not reliably detected
Singh et al. (2017) [46]	pNF-H; Plasma	BioVendor ELISA; 2000g/10 min, −20 °C, duplicate assays
Streijger et al. (2017) [47]	165-target panel, including gamma-enolase/NSE and neural/immune proteins; CSF	LC-MRM targeted mass spectrometry; −80 °C; batch randomization balanced AIS/time; below-LLOQ imputation

Table S6b. Biological matrices, assays and specimen handling		
**Report**	**Target and additional biomarkers; matrix**	**Assay and handling**
Du et al. (2018) [48]	NSE, S100B; Serum	ELISA; Xiamen Huijia/Shanghai Xinran; Clot 10 min at 25 °C, 3000 rpm/20 min, −80 °C; duplicate same-batch assays
Cuce et al. (2019) [40]	Cortisol, ACTH, DHEAS, thyroid hormones; additional sex hormones and prolactin; Serum	Roche Cobas cortisol electrochemiluminescence; 08:00–09:00 fasting; 1 and 250 µg ACTH tests on different days; cortisol at 0, 30, 60, 90, 120 min
Rieder et al. (2019) [49]	IL-6, GDNF, NGF, NSE; Serum	GE/R&D ELISAs; Elecsys-2010 NSE; −70 °C; duplicate assays, reported variation ≤5%
Capirossi et al. (2020) [50]	IL-6/IL-8, BDNF, NGF, NSE, total/phosphorylated tau and broad panel; CSF	MAGPIX/Luminex xMAP, 38 analytes retained; 2000g/10 min at 4 °C, −80 °C; duplicates
Holmström et al. (2020) [62]	GFAP, pNF-H; UCH-L1; CSF and plasma	Modified MSD electrochemiluminescence assays; Intraoperative CSF for tethering/tumour groups versus preoperative puncture in radiculopathy; −70 °C
Lin et al. (2021) [63]	IL-6, IL-8 plus cytokine/chemokine panel; CHRFAM7A genotype; EDTA plasma	MILLIPLEX/Luminex; Repeated central-catheter blood; multiple imputation 20 datasets; no uncritical inference that nonsignificant MCAR proves random missingness
Todd et al. (2021) [41]	IL-6; IL-10, IL-1ra and TNF-α; Plasma	MSD U-PLEX/QuickPlex; 12-h fast; EDTA plasma, two centrifugation steps, −80 °C; pre, immediate post and 90 min
Itodo et al. (2022) [18]	DHEA, DHEAS; testosterone and SHBG; Serum	ELISA (Abcam/Abnova); Entry and discharge, 07:00–14:00; centrifuged, stored −80 °C
Wichmann et al. (2022) [64]	IL-6, IL-8, GDNF and 92-protein inflammatory panel; CSF and EDTA plasma	Olink Target 96 proximity-extension assay, normalized log2 units; Serial lumbar puncture; normalized protein expression rather than pg/mL
Stukas et al. (2023) [51]	NfL, GFAP; Serum and CSF	Quanterix Simoa NF-Light/GFAP assays; 390 CSF and 421 serum specimens; 349 paired specimens from 113 patients; outcome/threshold-specific n differs
Wichmann et al. (2023) [65]	GFAP, NfL, NSE, IL-6/IL-8 plus cytokines; CSF, plasma; serum NSE excluded from analysis	Simoa HD-1; Roche Cobas NSE; MSD V-PLEX; Values exceeding assay range capped; below-detection cytokines set to half LLOD
Yang et al. (2023) [52]	IL-6, GFAP, NfL, S100B, tau; UCH-L1/spectrin breakdown products; CSF and serum	Immunoblot, ELISA and Simoa N4PB; Clinically necessary lumbar drain; −80 °C; outcome time varies
Agulló-Ortuño et al. (2024) [42]	IL-6, IL-8 and inflammatory panel; EDTA plasma	ProcartaPlex/Luminex; 2000 rpm/5 min, −80 °C; duplicates; 1% FDR for cytokine comparisons, correlation correction not established
Ding et al. (2024) [21]	CRP plus cellular inflammatory measures; Blood CRP (serum/plasma not specified)	Routine clinical testing; analytical platform not reported; See reported injury-to-sampling window

Table S6b. Biological matrices, assays and specimen handling		
**Report**	**Target and additional biomarkers; matrix**	**Assay and handling**
Haddadi et al. (2024) [53]	S100B; Serum and CSF	Serum electrochemiluminescence; CSF assay described incompletely; −80 °C; lumbar puncture or surgical CSF sampling
Pu et al. (2024) [20]	NSE, S100B; neuritin (not neurofilament); Serum-labelled specimens; methods also refer generically to serum/plasma	ELISA, Shanghai Senxiong; See reported injury-to-sampling window
Azad et al. (2025) [54]	cfDNA plus IL-6, NfH, GFAP/NfL, FABP3 and REST; Plasma	Bisulfite ddPCR plus 119-target NULISA immunoassay; Streck tubes; 814g/10 min then 5662g/30 min, −80 °C
Fraussen et al. (2025) [55]	NfL, GFAP, contactin-1; Plasma (lithium heparin)	Simoa HD-X; Luminex contactin-1; 400g/10 min then 1500g/10 min, −80 °C; blinded duplicates and batch-bridging samples
Morrison et al. (2025) [66]	CRP, IL-6, IL-8 and immune-mediator panel; Plasma; whole-blood cellular/transcript assays separate	ELISA plus Bio-Plex 48-plex; RNA sequencing and flow cytometry; Mean panel CV 5.4%; below-LLOQ values set to half LLOQ
Raheja et al. (2025) [56]	GFAP, S100B, pNF-H, NSE; Serum	BioVendor/DRG ELISAs; 2500 rpm/10 min, −80 °C
Seijas et al. (2025) [19]	DHEA/DHEAS; total/free testosterone and SHBG; serum	ELISA; fasting blood 07:00–14:00; 1800g, −80 °C; admission/discharge pairs on the same plate; biomarker technical completeness distinguished from clinical missingness
Vedantam et al. (2025) [67]	NfL, GFAP, BDNF, tau, IL-6/IL-8 plus cytokines/amyloid; Serum per processing methods	Simoa neuronal panel and CorPlex cytokines; Clot 30–60 min, 1300–1500g/10 min, −80 °C; blindedness not stated
Waliullah et al. (2025) [57]	S100B; Serum	ELISA E-EL-H1297; Immediately processed and stored −80 °C
Belogurov et al. (2026) [68]	IL-6/IL-8 and cytokine/chemokine panel; Li-heparin plasma and CSF	Multiplex cytokine profiling; Plasma 1500g/10 min; −70 °C; repeated intradural catheter sampling
Hei et al. (2026) [58]	GFAP, tau, contactin-1; Serum, as reported in the collection and assay Methods; conflicting plasma terminology in the title and Results	Quanzhou Ruixin ELISAs; See reported injury-to-sampling window

Table S6b. Biological matrices, assays and specimen handling

Report	Target and additional biomarkers; matrix	Assay and handling
Sheinberg et al. (2026) [59]	Exosomal GFAP, NfH, tau, S100B, IL-6/IL-8, BDNF/NGF/GDNF, broader proteins and miRNAs; CSF exosome isolates; serum exosomal RNA comparator	Bio-Plex multiplex; RNA sequencing; independent qPCR confirmation of one miRNA; Exosome precipitation kit; CSF processed within 1 h, 2000g/10 min, −80 °C; multiplex assays blinded and duplicate
Note: Serum, plasma, CSF and exosome-enriched fractions are not treated as interchangeable. Normalized proteomic expression is not an absolute concentration. Limits of detection/quantification, imputation and haemolysis exclusions qualify individual results. NR handling details remain unresolved rather than inferred from a different report.		

Abbreviations: AIS, American Spinal Injury Association Impairment Scale; CSF, cerebrospinal fluid; CRP, C-reactive protein; hsCRP, high-sensitivity C-reactive protein; IL, interleukin; IL-1ra, interleukin-1 receptor antagonist; IL-6, interleukin-6; IL-8, interleukin-8; IL-10, interleukin-10; TNF-α, tumour necrosis factor alpha; BDNF, brain-derived neurotrophic factor; NGF, nerve growth factor; GDNF, glial cell line-derived neurotrophic factor; GFAP, glial fibrillary acidic protein; NSE, neuron-specific enolase; NfL, neurofilament light chain; NfH, neurofilament heavy chain; pNF-H, phosphorylated neurofilament heavy chain; S100B, S100 calcium-binding protein B; PTH, parathyroid hormone; ACTH, adrenocorticotropic hormone; DHEA, dehydroepiandrosterone; DHEAS, dehydroepiandrosterone sulfate; SHBG, sex hormone-binding globulin; PGE2, prostaglandin E2; LTB4, leukotriene B4; EDTA, ethylenediaminetetraacetic acid; ELISA, enzyme-linked immunosorbent assay; LC-MRM, liquid chromatography multiple-reaction monitoring; MSD, Meso Scale Discovery; Simoa, single molecule array; LLOD, lower limit of detection; LLOQ, lower limit of quantification; FDR, false discovery rate; CV, coefficient of variation; RNA, ribonucleic acid; miRNA, microRNA; qPCR, quantitative polymerase chain reaction; ddPCR, droplet digital polymerase chain reaction; cfDNA, cell-free deoxyribonucleic acid; NR, not reported; CHRFAM7A, CHRNA7–FAM7A fusion gene; MCAR, missing completely at random; UCH-L1, ubiquitin C-terminal hydrolase L1; FABP3, fatty acid-binding protein 3; NULISA, nucleic acid-linked immunosandwich assay; REST, RE1-silencing transcription factor.

**Table S6c. Established or possible cohort dependence**		
**Reports**	**Evidence of dependence**	**Interpretive consequence**
Morse et al. (2008) [36]; Goldstein et al. (2017) [39]	26 participants retested in the later chronic CRP cohort.	Partial overlap; not fully independent replication.
Kwon et al. (2010) [33]; Kwon et al. (2017) [34]	Kwon 2010 recruited March 2006–March 2008; Kwon 2017 reports the first 50 patients recruited March 2006–August 2011 and explicitly extends the 2010 series.	Overlapping source cohort; exact common analytical sample can differ by marker/outcome.
Pouw et al. (2014) [44]; Kwon et al. (2010) [33]; Kwon et al. (2017) [34]	Pouw Vancouver arm shares registration and recruitment period.	Possible overlap; exact shared n unresolved.
Streijger et al. (2017) [47]; Stukas et al. (2023) [51]	NCT01279811/H10-01091 programme shared.	Exact shared n unresolved.
Itodo et al. (2022) [18]; Seijas et al. (2025) [19]	Both reports use SwiSCI Biobank admission/discharge specimens from June 2016–January 2021; analysis samples 86 and 80. Exact participant overlap is not reported.	Possible substantial overlap; complementary hormone-trajectory and hormone–function analyses, not established independent replication.
Wichmann et al. (2022) [64]; Wichmann et al. (2023) [65]	Same 15 Aarhus participants, NCT03505463; 11 completed follow-up.	Two complementary reports from one cohort.
Stukas et al. (2023) [51]; Kwon et al. (2010) [33]; Kwon et al. (2017) [34]	Stukas recruits across 2006–2019 from NCT00135278/NCT01279811.	Shared source programme; repeated participants possible. Do not sum enrolled samples.
Ding et al. (2024) [21]; Hei et al. (2026) [58]	Shared Nanchang institution; different periods (2015–2022 versus February–April 2025).	Institutional similarity alone does not establish participant overlap.
Morrison et al. (2025) [66]	12 participants previously reported outside this included set. Multicentre recruitment in 2016–2020 includes Vancouver; shared institutions alone do not establish overlap with the earlier biomarker cohorts.	Not counted as an additional included report or independent validation.
Note: For the other included reports, overlap was not established from the available full texts; this does not prove independence. The exact number of independent participant cohorts across the 41 reports could not be established because cohort reuse, partial overlap and pooling of recruitment series were not fully resolvable across publications. Publication counts are report counts.		

Abbreviations: CRP, C-reactive protein.

Table S7. Target biomarker domains and interpretation

Domain	Targets	What the measurement can address	What it does not establish
E: neuroendocrine	Cortisol, ACTH, DHEA/DHEAS, thyroid hormones	Basal/stimulated endocrine physiology; concurrent severity/pain or temporal endocrine change.	A common HPA recovery mechanism or endocrine mediation of neurological change.
I: inflammation	CRP, IL-6, IL-8	Systemic/CSF inflammatory state and tested clinical relationships.	Cord-specific inflammation from peripheral concentration alone.
T: neurotrophic	BDNF, NGF, GDNF	Quantifiable fluid-factor response and tested clinical association.	Independent neural reorganization, regeneration or plasticity.
N: neural injury	GFAP, NfL/NfH/pNF-H, S100B, NSE, tau, contactin-1	Injury burden, severity discrimination and exploratory prognosis.	Direct descending transmission, tissue repair or treatment responsiveness.
Additional measures	PTH, testosterone, other cytokines, cfDNA, neuritin and exosomal RNA within qualifying reports	Context or multi-analyte model contributions.	Independent target eligibility or equivalent evidence for every assayed constituent.
Domain counts	E=5; I=19; N=26; T assayed=8, quantitatively usable=6	Non-exclusive report counts; two Kwon reports tested growth factors without reliable detection.	Independent replication counts, positive-effect counts or certainty grades.

Abbreviations: CSF, cerebrospinal fluid; CRP, C-reactive protein; IL, interleukin; IL-6, interleukin-6; IL-8, interleukin-8; BDNF, brain-derived neurotrophic factor; NGF, nerve growth factor; GDNF, glial cell line-derived neurotrophic factor; GFAP, glial fibrillary acidic protein; NSE, neuron-specific enolase; NfL, neurofilament light chain; NfH, neurofilament heavy chain; pNF-H, phosphorylated neurofilament heavy chain; S100B, S100 calcium-binding protein B; PTH, parathyroid hormone; ACTH, adrenocorticotropic hormone; HPA, hypothalamic–pituitary–adrenal; DHEA, dehydroepiandrosterone; DHEAS, dehydroepiandrosterone sulfate; RNA, ribonucleic acid; cfDNA, cell-free deoxyribonucleic acid; E, neuroendocrine biomarker domain; I, inflammatory biomarker domain; T, neurotrophic biomarker domain; N, neural injury biomarker domain.

Table S8a. Phase-by-phenotype synthesis		
**Report and phase**	**Clinical phenotype**	**Biological sampling, intervention and outcome timing**
Mechanick et al. (1997) [35]; Mixed acute/subacute/chronic	49 SCI; 29 complete/20 incomplete	Mean 76.5 days, range 11–371 days (overall sample); Exposure: Inpatient care, rehabilitation achieved dose not quantified
Morse et al. (2008) [36]; Chronic	63 chronic SCI men	Mean 21±13 years after injury; Exposure: Not quantified
Kwon et al. (2010) [33]; Acute	27 traumatic SCI; AIS A–C	Every 6–8 h through 72 h; primary 24-h biomarkers; motor outcome 6 months; Exposure: Not quantified
Hayakawa et al. (2012) [43]; Acute→subacute	14 traumatic cervical SCI	Plasma 6 h–21 days, variable missing draws; final AIS 43–556 days; Exposure: Not quantified
Harness et al. (2014) [37]; Chronic	15 chronic SCI; AIS A–C	Mean 63.9±54.4 months; Exposure: Single 2 h bout: four approximately 30 min modalities; load-bearing 30 min; BWSTT 10×2 min/1 min rest, 60% body-weight support; WBV 15×1 min/1 min rest; FES 30 min
Pouw et al. (2014) [44]; Acute	16 traumatic SCI; AIS A–D	Single CSF within 24 h (mean 14, range 3–24 h); 6- or 12-month AIS follow-up; Exposure: Not quantified
Takahashi et al. (2014) [60]; Chronic disease; acute worsening subgroup	14 analysed compressive myelopathy	Preoperative CSF; worsening defined as ≥2 JOA points within preceding month; 6-month follow-up; Exposure: Not quantified
Ungureanu et al. (2014) [45]; Acute	15 traumatic SCI; 8 complete/7 incomplete	CSF 6–12 h then daily to discharge/death, up to 11–12 days; follow-up 10–24 months; Exposure: Not quantified
Ahadi et al. (2015) [17]. Acute. SCI age composition unresolved	26 SCI among 35 trauma participants. Overall age range 16–64 years, without a separate SCI range	Serum 24, 48 and 72 h; early postoperative survival; Exposure: Not quantified
Kato et al. (2015) [61]; Mixed symptom duration; perioperative	26 cervical compressive myelopathy	Symptoms median 10 months, range 1 month–13 years; serum preoperatively and 1 week; clinical follow-up 3 months; Exposure: Not quantified
Allison et al. (2016) [38]; Chronic	20 chronic SCI; AIS A–D	At least 2 years post injury; 12-week dietary intervention; Exposure: 12 weeks diet plus supplements; mean reported dietary adherence 89% (70–100%); one participant missing later diet records
Goldstein et al. (2017) [39]; Chronic	338 chronic SCI	Mean 17.3±13.3 years after injury; Exposure: Not quantified
Kwon et al. (2017) [34]; Acute	50 traumatic SCI; AIS A–C	Primary CSF 24 h (mean 27.4±8.6 h); serial 72–120 h; outcome 6 months; Exposure: Not quantified
Singh et al. (2017) [46]; Acute→subacute	28 traumatic thoracolumbar SCI	Plasma day 3/7/14/28; AIS to day 90; Exposure: Not quantified

Table S8a. Phase-by-phenotype synthesis		
**Report and phase**	**Clinical phenotype**	**Biological sampling, intervention and outcome timing**
Streijger et al. (2017) [47]; Acute	29 traumatic SCI; AIS A–C	CSF 24/48/72 h; principal analysis 24 h; 6-month motor outcome; Exposure: Not quantified
Du et al. (2018) [48]; Acute	60 traumatic SCI; 21 improve/39 do not	Admission mean 3.4–3.6 h; days 2, 4, 6, 8, 10, 12, 14; outcome 6 months; Exposure: Not quantified
Cuce et al. (2019) [40]; Chronic	22 SCI; 15 NP/7 without NP	Mean 6.7 years with pain and 3.4 years without pain; Exposure: Two pharmacological challenges, not a rehabilitation course
Rieder et al. (2019) [49]; Acute	52 isolated traumatic SCI	48 h and day 7; neurological/functional endpoint 6 months; Exposure: Prescribed: inpatient physiotherapy twice/day, then outpatient ≥6 months 3×/week; delivered dose/adherence not reported
Capirossi et al. (2020) [50]; Acute sampling; mixed discharge phase	9 analysed traumatic SCI	CSF 12–24 h; admission median 3 days; discharge median 132 days, range 47–320; Exposure: Inpatient rehabilitation; actual sessions/minutes not quantified
Holmström et al. (2020) [62]; Chronic disease; new deterioration	12 acquired cord-tethering patients	Years after primary injury; new worsening 1–>24 months; 3-month postoperative outcome; 15 surgical occasions in 12 patients; Exposure: Not quantified
Lin et al. (2021) [63]; Acute→subacute	45 traumatic SCI; AIS A–D	First draw mean 4.66±3.77 days; 1–3 draws/week, severe through 6 weeks and mild through 3 weeks; Exposure: Not quantified
Todd et al. (2021) [41]; Chronic	8 analysed chronic SCI with NP	Mean 17.8±8.9 years after SCI, range 3–36 years; immediate and 90-minute outcomes; Exposure: 30 min arm cranking at 60% peak power; RPE-triggered reduction to 50%; actual RPE differed by lesion group
Itodo et al. (2022) [18]; Acute/subacute entry; rehabilitation follow-up	86 SCI; 70 men/16 women	Entry median 15.5 days (IQR 10–27); rehabilitation duration median 5.6 months (IQR 4.2–7.5); Exposure: Not quantified
Wichmann et al. (2022) [64]; Acute→subacute	15 traumatic SCI; AIS A–D	Median 13 h/day 0, day 9 and day 148; latter is <6 months despite author label chronic; Exposure: Not quantified
Stukas et al. (2023) [51]; Acute	118 traumatic SCI; AIS A–C	Samples 4.3–107 h grouped at 24/48/72/96 h; outcome 6 months; Exposure: Not quantified
Wichmann et al. (2023) [65]; Acute→subacute	Same 15 traumatic SCI	Median 13 h, 9 days and 148 days; Exposure: Not quantified
Yang et al. (2023) [52]; Acute	15 traumatic SCI; AIS A/B	Daily days 1–5; discharge <3 months; Exposure: Not quantified
Agulló-Ortuño et al. (2024) [42]; Subacute entry	40 analysed subacute SCI; AIS C/D	Mean 3.6±1.3 and 3.1±0.8 months after injury; weeks 0/6/12; Exposure: 18 aquatic sessions, 3/week, 40 min over 6 weeks plus usual treatment; RPE monitoring; achieved minutes/adherence not reported
Ding et al. (2024) [21]; Acute	374 traumatic cervical SCI	Blood within 12 h; MRI within 48 h; outcome at 1 year; Exposure: Not quantified

Table S8a. Phase-by-phenotype synthesis

Report and phase	Clinical phenotype	Biological sampling, intervention and outcome timing
Haddadi et al. (2024) [53]; Acute	31 traumatic SCI; 9 complete/22 incomplete	24–48 h post injury; no later recovery endpoint analysed; Exposure: Not quantified
Pu et al. (2024) [20]; Unclassified; combined neurotrauma	60 combined SCI–TBI	Admission biological sampling time and GOS assessment horizon insufficiently specified; Exposure: Not quantified
Azad et al. (2025) [54]; Acute deficit; mixed aetiology	50 traumatic/compressive SCI	Presurgical ≤7 days from deficit, median 1 day (IQR 0–2); 6-month AIS; Exposure: Not quantified
Fraussen et al. (2025) [55]; Acute→subacute	37 traumatic SCI	Baseline 0–4 days, 3/6/12/18 weeks; latest clinical endpoint, not a uniform follow-up date; Exposure: Not quantified
Morrison et al. (2025) [66]; Acute	36 traumatic SCI; AIS A–D	Research draws 0–4 days; no longitudinal recovery analysis in this report; Exposure: Not quantified
Raheja et al. (2025) [56]; Acute	26 traumatic SCI; 9 followed	Admission (<24 h), days 2 and 7; 6-month endpoint available in 9/26; Exposure: Not quantified
Seijas et al. (2025) [19]; Acute/subacute entry; typical baseline subacute	80 traumatic/non-traumatic SCI during first inpatient rehabilitation	Baseline blood median 42 days after SCI (IQR 30–54), eligibility ≤120 days; median follow-up 167 days (128–224); serial SCIM-III and discharge assessment; achieved active rehabilitation dose not quantified
Vedantam et al. (2025) [67]; Mixed symptom duration; perioperative	20 degenerative cervical myelopathy	Symptoms median 12 months (range 1–72); preoperative draw median 3.4 weeks before surgery; 3 months postoperative; Exposure: Not quantified
Waliullah et al. (2025) [57]; Acute→subacute	26 traumatic SCI	Within 24 h, 2 weeks and 6 weeks; Exposure: Not quantified
Belogurov et al. (2026) [68]; Chronic tumour; acute surgical injury	8 analysed intramedullary-tumour patients	Preoperative, then 8/12/24/48/72/120/168 h; discharge mean 8.6 days; Exposure: Rehabilitation from day 2 to discharge; actual dose not quantified
Hei et al. (2026) [58]; Acute	71 traumatic cervical SCI	Within 24 h of admission (admission ≤48 h injury); MRI within 48 h; 6 months after discharge per methods; Exposure: Not quantified
Sheinberg et al. (2026) [59]; Acute	25 traumatic SCI; AIS A–C	Days 1/3/5; neurological follow-up 3 months in Methods; ≥3 months in model description; Exposure: Not quantified

Note: Acute ≤14 days, subacute >14 days–6 months, chronic >6 months from the initiating injury. Exact intervals take precedence. Day 148 is subacute. Surgery/worsening dates are kept distinct from duration of preceding chronic disease. Time after admission is not automatically time after injury.

Abbreviations: SCI, spinal cord injury; TBI, traumatic brain injury; AIS, American Spinal Injury Association Impairment Scale; CSF, cerebrospinal fluid; MRI, magnetic resonance imaging; JOA, Japanese Orthopaedic Association score; GOS, Glasgow Outcome Scale; NP, neuropathic pain; IQR, interquartile range; BWSTT, body-weight-supported treadmill training; FES, functional electrical stimulation; WBV, whole-body vibration; RPE, rating of perceived exertion.

Table S8b. Study-level biomarker and relationship map						
**Report**	**E**	**I**	**T**	**N**	**Clinical relationship actually examined**	**Residual-function/imaging integration**
Mechanick et al. (1997) [35]	M	—	—	—	Concurrent severity/endocrine tests (null target associations)	No tested fluid-biomarker–circuit relationship
Morse et al. (2008) [36]	—	M	—	—	Concurrent CRP–mobility association	No tested fluid-biomarker–circuit relationship
Kwon et al. (2010) [33]	—	M	A	M	Severity discrimination; motor prognosis; null pain	No tested fluid-biomarker–circuit relationship
Hayakawa et al. (2012) [43]	—	—	—	M	Serial marker–AIS comparisons; final-status groups	No tested fluid-biomarker–circuit relationship
Harness et al. (2014) [37]	M	—	M	—	Single-bout change; null BDNF–motor-score test	No tested fluid-biomarker–circuit relationship
Pouw et al. (2014) [44]	—	—	—	M	Severity and small-subgroup conversion comparisons	No tested fluid-biomarker–circuit relationship
Takahashi et al. (2014) [60]	—	—	—	M	Severity/worsening; null JOA recovery association	No tested fluid-biomarker–circuit relationship
Ungureanu et al. (2014) [45]	—	—	—	M	Described serial marker/AIS relationships; no adjusted prognosis	No tested fluid-biomarker–circuit relationship
Ahadi et al. (2015) [17]	—	—	—	M	Initial severity/early survival; no longitudinal function	No tested fluid-biomarker–circuit relationship
Kato et al. (2015) [61]	—	—	—	M	Severity association; null recovery contrast	Perioperative monitoring context; no marker–transmission recovery pathway
Allison et al. (2016) [38]	—	M	—	—	Randomized pain/cytokine contrasts; exploratory change correlations	No tested fluid-biomarker–circuit relationship
Goldstein et al. (2017) [39]	—	M	—	—	Concurrent adjusted CRP–mobility association	No tested fluid-biomarker–circuit relationship
Kwon et al. (2017) [34]	—	M	A	M	Severity discrimination; prospective conversion/motor prognosis	No tested fluid-biomarker–circuit relationship
Singh et al. (2017) [46]	—	—	—	M	Concurrent/serial biomarker–AIS relationships	No tested fluid-biomarker–circuit relationship
Streijger et al. (2017) [47]	—	—	—	M	Severity/prognosis testing; adjusted/FDR distinctions	No tested fluid-biomarker–circuit relationship
Du et al. (2018) [48]	—	—	—	M	Prospective AIS improvement discrimination	No tested fluid-biomarker–circuit relationship
Cuce et al. (2019) [40]	M	—	—	—	Concurrent pain–stimulated cortisol association	No tested fluid-biomarker–circuit relationship

**Table S8b. Study-level biomarker and relationship map**						
**Report**	**E**	**I**	**T**	**N**	**Clinical relationship actually examined**	**Residual-function/imaging integration**
Rieder et al. (2019) [49]	—	M	M	M	Null prospective neurological/FIM associations	No tested fluid-biomarker–circuit relationship
Capirossi et al. (2020) [50]	—	M	M	M	Prospective motor/independence correlations	No tested fluid-biomarker–circuit relationship
Holmström et al. (2020) [62]	—	—	—	M	Null clinical-score associations; perioperative series	Surgical/MRI context; no tested biomarker–transmission pathway
Lin et al. (2021) [63]	—	M	—	—	Genotype/time/pain–cytokine interactions; not treatment interaction	No tested fluid-biomarker–circuit relationship
Todd et al. (2021) [41]	—	M	—	—	Single-bout change; immediate pain-change correlation	No tested fluid-biomarker–circuit relationship
Itodo et al. (2022) [18]	M	—	—	—	Concurrent severity association; endocrine trajectory only	No tested fluid-biomarker–circuit relationship
Wichmann et al. (2022) [64]	—	M	M	—	Prospective plasma GDNF–conversion association	No tested fluid-biomarker–circuit relationship
Stukas et al. (2023) [51]	—	—	—	M	Prospective conversion/motor models plus sensory ZPP	Clinical sensory ZPP
Wichmann et al. (2023) [65]	—	M	—	M	Severity association; null conversion/SCIM tests	No tested fluid-biomarker–circuit relationship
Yang et al. (2023) [52]	—	M	—	M	Severity; mostly null discharge improvement tests	No tested fluid-biomarker–circuit relationship
Agulló-Ortuño et al. (2024) [42]	—	M	—	—	Randomized sequence contrast; exploratory change–10MWT association	No tested fluid-biomarker–circuit relationship
Ding et al. (2024) [21]	—	M	—	—	Biomarker+MRI future-status prognosis	MRI BASIC/lesion morphology
Haddadi et al. (2024) [53]	—	—	—	M	Initial completeness discrimination only	No tested fluid-biomarker–circuit relationship
Pu et al. (2024) [20]	—	—	—	M	Combined-neurotrauma prognosis; timing unclear	No tested fluid-biomarker–circuit relationship
Azad et al. (2025) [54]	—	M	—	M	Multi-analyte prospective conversion model	No tested fluid-biomarker–circuit relationship
Fraussen et al. (2025) [55]	—	—	—	M	Prospective conversion/motor models	No tested fluid-biomarker–circuit relationship
Morrison et al. (2025) [66]	—	M	—	—	Concurrent completeness/inflammatory phenotypes	No tested fluid-biomarker–circuit relationship
Raheja et al. (2025) [56]	—	—	—	M	Severity and later AIS-status discrimination; severe attrition	No tested fluid-biomarker–circuit relationship

Table S8b. Study-level biomarker and relationship map						
**Report**	**E**	**I**	**T**	**N**	**Clinical relationship actually examined**	**Residual-function/imaging integration**
Seijas et al. (2025) [19]	M	—	—	—	Baseline DHEA/DHEAS and subsequent functioning; longitudinal group×time associations and null discharge estimates	No tested fluid-biomarker–circuit relationship
Vedantam et al. (2025) [67]	—	M	M	M	Diagnosis; presurgical marker–postoperative pinch change	No tested fluid-biomarker–circuit relationship
Waliullah et al. (2025) [57]	—	—	—	M	Concurrent/serial severity comparisons	No tested fluid-biomarker–circuit relationship
Belogurov et al. (2026) [68]	—	M	—	—	Perioperative cytokine–discharge status correlations	MEP/D-wave co-measured; no connecting biomarker analysis
Hei et al. (2026) [58]	—	—	—	M	Biomarker+MRI+AIS prospective conversion model	MRI IMLL/compression
Sheinberg et al. (2026) [59]	—	M	M	M	Exosomal protein severity/improvement models	No tested fluid-biomarker–circuit relationship

Note: M = target biomarker with a quantitative human measurement; A = target assayed without reliable quantitative detection; — = target domain not assessed. These symbols represent measurement, not a positive clinical association. Null results remain tested relationships. No report establishes formal causal mediation by achieved rehabilitation dose or a treatment-by-biomarker interaction. Randomized contrasts in Allison/Agulló-Ortuño do not make their observational marker correlations randomized.

Abbreviations: AIS, American Spinal Injury Association Impairment Scale; CRP, C-reactive protein; BDNF, brain-derived neurotrophic factor; GDNF, glial cell line-derived neurotrophic factor; MRI, magnetic resonance imaging; BASIC, Brain and Spinal Injury Center; IMLL, intramedullary lesion length; SCIM, Spinal Cord Independence Measure; FIM, Functional Independence Measure; JOA, Japanese Orthopaedic Association score; 10MWT, 10-metre walk test; FDR, false discovery rate; MEP, motor evoked potential; ZPP, zone of partial preservation; E, neuroendocrine; I, inflammatory; T, neurotrophic; N, neural and glial injury; M, quantitative measurement reported; A, assayed without reliable quantitative findings.

Table S9. Quantitative and null findings with analysis-specific interpretation			
**Report**	**Clinical endpoint and analytical sample**	**Reported finding and uncertainty**	**Interpretation or reporting limitation**
Mechanick et al. (1997) [35]	ASIA severity, lesion level, endocrine/metabolic group comparisons; Principal SCI n=49	Serum cortisol not significantly different between groups and ASIA motor score not correlated with measured parameters. Contextual PTH: SCI 13.4±1.1 versus TBI 27.3±3.9 pg/mL, mean±SEM, p<.000009. Complete SCI 11.1±1.0 versus incomplete 13.9±1.5 pg/mL, p<.03. Severity-group effects on PTH/1, 25D abolished with ASIA motor score as covariate.	n=49 SCI/33 TBI; SEM, not SD; Retrospective clinically selected sample, age differences, subgroups as small as5, no longitudinal prognosis
Morse et al. (2008) [36]	Motorized/manual wheelchair, assisted or independent walking; Principal SCI n=63	Adjusted geometric mean CRP: motorized wheelchair 5.11, manual wheelchair 2.19 (p=0.085), assisted walking 1.41 (p=0.005), independent walking 1.63 mg/L (p=0.027), compared with motorized mobility.	Confidence interval not reported; Small selected sample; adiposity and illness confounding; mobility groups in abstract sum to pre-exclusion n=64
Kwon et al. (2010) [33]	Baseline AIS, six-month upper-extremity motor recovery, neuropathic pain; Principal SCI n=27	GFAP/S100B/IL-8 severity model apparent accuracy 24/27=88.9%; temporal 18/9 internal split 7/9=78%. Cervical motor prediction n=20 accuracy 70% versus baseline AIS 65%. No late pain association.	Bootstrap internal severity accuracy 77% (95% CI 44–100%); small n; Small model-development sample, variable detectable analytes; cohort trial does not randomize biomarker exposure
Hayakawa et al. (2012) [43]	AIS at admission, day 7/30 and final follow-up; Principal SCI n=14	pNF-H detected in 11/14 by 96 h. Final AIS A n=4 versus C n=5: mean 96-h values 5260 versus 449 pg/mL. Illustrative initial AIS A→C case had lower levels.	Confidence interval not reported; Small groups; grouping by final AIS conflates severity and prognosis; censored values set to zero; telephone outcome in some patients
Harness et al. (2014) [37]	Acute biomarker change; AIS lower-extremity motor-score and injury-duration correlations; Principal SCI n=15	BDNF exercise effect $F(4, 52)=.14$, $p=.97$; cortisol $p=.07$. BDNF change unrelated to lower-extremity motor score or injury duration. Prolactin decreased, mean change −26.2±40.1%.	BDNF n=15; complete cortisol sequence n=12; Fixed modality sequence, no concurrent control, small selected sample, cortisol missingness, immediate sampling only
Pouw et al. (2014) [44]	AIS severity and conversion; Principal SCI n=16	NSE motor-complete versus incomplete 83.7±75 versus 25.3±17 µg/L; n=16. Non-converting baseline AIS A patients had 9.6-fold higher GFAP and 2.5-fold higher tau than converters. Descriptive small-subgroup comparisons.	Confidence interval not reported; 7/23 exclusions including missing follow-up and blood-contaminated CSF; NfH n=15; heterogeneous follow-up; no adjusted model
Takahashi et al. (2014) [60]	JOA severity and postoperative recovery rate; Principal SCI n=14	CSF pNF-H 2127.1±556.8 pg/mL in worsening versus 175.8±27.5 in chronic myelopathy. No significant correlation with 6-month JOA recovery rate (p=0.128).	Confidence interval not reported; Small groups, unclear 15→14 discrepancy, no adjusted analysis; acute deterioration is not acute traumatic SCI
Ungureanu et al. (2014) [45]	AIS trajectory and survival; Principal SCI n=15	CSF pNF-H was higher in complete injuries; individual kinetic curves described alongside subsequent AIS grades. No adjusted prognostic model or inferential recovery effect size.	Individual ng/mL values reported; no correlation coefficient/CI establishing recovery prediction; Variable lumbar-puncture schedules, refusal and death; small series; unadjusted trajectories
Ahadi et al. (2015) [17]	Initial AIS grade, spinal canal narrowing, early mortality; Principal SCI n=26	Markers were generally elevated after SCI; severity-stratified and survivor/non-survivor comparisons reported. Eight postoperative deaths. No longitudinal functional-recovery prediction demonstrated.	26 SCI overall; marker-specific analysis denominators incompletely reported; SCI age composition unresolved; inconsistent text/figure significance and sample labels (NSE figure n=23); outcome-dependent attrition; group CIs not reported

Table S9. Quantitative and null findings with analysis-specific interpretation

Report	Clinical endpoint and analytical sample	Reported finding and uncertainty	Interpretation or reporting limitation
Kato et al. (2015) [61]	mJOA severity/recovery rate; recent symptom aggravation and MRI; Principal SCI n=26	Preoperative detectable pNF-H in 6/26: mJOA median 10.5 versus 13.0 (p=0.005); postoperative recovery rates 28% versus 20% (p=0.84). Postoperative serum available in 18/26.	Confidence interval not reported; Very young non-SCI comparator group; serum/plasma wording inconsistent; postoperative attrition; no adjusted prognostic model
Allison et al. (2016) [38]	Neuropathic Pain Questionnaire sensory, affective and sensitivity subscales; CES-D adjustment; Principal SCI n=20	Sensory pain group×time p<0.01, reported Cohen d=1.29; mean 32.8→19.8 intervention versus 18.1→21.3 control. IL-6 between-group change p=0.13; CRP p=0.10. Exploratory change model with IL-2, IFN-γ and PGE2: R^2=0.474, n=20.	All 20 completed; biomarker and model CIs not reported; Unblinded self-reported pain, unclear allocation concealment, multiple markers/stepwise models; randomization does not establish mediation
Goldstein et al. (2017) [39]	Mobility mode; adiposity, catheter use and recent illnesses; Principal SCI n=338	Mobility trend remained associated with CRP in 4/5 adjusted adiposity models (p=0.001–0.018), not one regional-fat model (p=0.069). Neurological level/completeness not associated after adjustment.	Confidence interval not reported; Selection, reverse causality, self-reported mobility and recent illness; no longitudinal outcome
Kwon et al. (2017) [34]	AIS classification/conversion and motor-score recovery; Principal SCI n=50	AIS classification accuracy 84%, leave-one-out 76%. Six-month conversion apparent accuracy 83.3% (n=48; 22 converters/26 non-converters). Inflammatory-index AUC 0.883 (SE 0.052; 95% CI 0.781–0.985); structural-index AUC 0.857 (SE 0.055; 95% CI 0.749–0.964).	Published AUCs have both SEs and 95% CIs; ROC estimates derive from the development sample. Small selected subgroups; data-driven indices; baseline severity and level confounding; leave-one-out validation of LDA classification, without external validation.
Singh et al. (2017) [46]	Serial AIS severity and course; Principal SCI n=28	Day-3 pNF-H 6.40±2.49 versus 0.54±0.27 ng/mL in controls; AIS numeric code A=5 to E=1; concurrent correlation described as mild. Individual changes accompany clinical trajectories.	Confidence interval not reported; Selected non-surgical population; ordinal AIS analysed numerically; no robust adjusted prognosis; reports of late peak days internally inconsistent
Streijger et al. (2017) [47]	Baseline AIS, six-month conversion and total motor-score change; Principal SCI n=29	27 proteins associated with baseline AIS after FDR correction. No conversion associations survived multiple-testing adjustment. After baseline AIS, level and motor adjustment, only coagulation factor XII survived FDR (adjusted p=0.0079). Cross-validated motor model R^2=0.52.	Confidence interval not reported; High-dimensional small-n selection; below-quantification imputation; internal validation; AIS-conversion null after correction
Du et al. (2018) [48]	AIS improvement ≥1 grade at 6 months; Principal SCI n=60	NSE AUC 0.78 (95% CI 0.66–0.89), cut-off 29.07 µg/L; S100B AUC 0.76 (0.63–0.89), cut-off 1.67 µg/L. Baseline motor/sensory scores differed strongly between outcome groups.	n=60; 21 improved and 39 not improved; AUC CIs reported; baseline severity imbalance; Baseline severity confounding; reversed group labels in reported baseline biomarker sentence; derivation cut-offs; no external validation
Cuce et al. (2019) [40]	NRS, LANSS; basal and stimulated endocrine response; Principal SCI n=22	NP subgroup NRS correlated with peak and AUC cortisol responses: low-dose peak r=0.52, p=.047, AUC r=.58, p=.024; standard-dose peak/AUC r=.58, p=.025/.023. Peak/AUC NP versus healthy controls not significantly different.	Spearman correlations, n=15; confidence intervals not reported; Small selected sample, excludes HPA-active medication and other pain, no temporal recovery analysis, multiple correlations
Rieder et al. (2019) [49]	ASIA motor/sensory scores; FIM total; Principal SCI n=52	No significant biomarker association with 6-month neurological or FIM outcomes at either sampling time. Only 16/52 had paired biomarkers; analysis n varied: NSE 52, NGF/GDNF 50, IL-6 36.	No effect-estimate CIs reported; FIM total does not provide an isolated cognitive-domain result; 19% mortality, only 16 paired samples, unclear correlation-specific follow-up n; no adjustment

Table S9. Quantitative and null findings with analysis-specific interpretation

Report	Clinical endpoint and analytical sample	Reported finding and uncertainty	Interpretation or reporting limitation
Capirossi et al. (2020) [50]	ISNCSCI motor/sensory, SCIM independence, Modified Ashworth spasticity; Principal SCI n=9	BDNF–discharge motor r=0.678 and motor change r=0.557; IL-8–motor change r=−0.505; p-tau–SCIM discharge r=0.700. Coefficients reflect exploratory n=9 correlations without confidence intervals, not validated prognosis.	No correlation CIs or multiplicity-controlled confirmatory analysis; n=9; Death excluded from final cohort; high-dimensional unadjusted correlations, co-injury including brain trauma, no dose analysis or validation
Holmström et al. (2020) [62]	AIS, JOACMEQ and EQ-5D; postoperative status; Principal SCI n=12	CSF GFAP/pNF-H higher than radiculopathy controls; plasma groups not different. Biomarkers did not correlate with preoperative AIS, EQ-5D or JOACMEQ; descriptive repeated surgical cases.	Confidence interval not reported; Selected heterogeneous tethering; tumour controls may express markers; 15 occasions are not 15 independent patients; no adjusted recovery relation
Lin et al. (2021) [63]	Numerical pain scale and Braden pressure-ulcer risk; initial AIS severity; Principal SCI n=45	Severe-SCI genotype×time interaction for IL-8 p=0.005. IL-13 genotype×pain interaction p=0.013; IL-6/IL-8 genotype×pain interactions not significant. No neurological recovery model.	Confidence interval not reported; Missing cytokines up to 77%; >20%-missing variables excluded, others multiply imputed; unequal observation windows; repeated measures and confounding
Todd et al. (2021) [41]	Neuropathic Pain Scale; Feeling Scale and Felt Arousal Scale; Principal SCI n=8	Baseline IL-1ra correlated with immediate pain change, r=0.833, p=0.01 (n=8), not sustained at 90 minutes. Tetraplegia pain Hedges g_av=−0.47; reported CI −0.32 to 2.02 is retained as reported, not treated as a validated effect-size CI.	Correlation n=8; lesion subgroups n=4 each; correlation CI not reported; Two exclusions (pressure sore and failed phlebotomy); n=4 subgroup correlations; post-hoc analyses; no control; paraplegia exceeded intended intensity
Itodo et al. (2022) [18]	Baseline injury completeness and level; longitudinal endocrine change, sexual-function questionnaire; Principal SCI n=86	Men: adjusted DHEAS increase 1.77 μmol/L (95% CI 0.73–2.81); total testosterone increase 3.96 nmol/L (1.37–6.56). Baseline incomplete versus complete SCI DHEAS β=−1.78 (−3.43 to −0.12). No analysed endocrine-change–neurological-recovery relationship.	Adjusted mixed models: age, BMI, level, completeness, injury duration, opioids and corticosteroids; Sex-specific small groups, baseline neurological data missing in 14; 07:00–14:00 sampling; opioid effects; no recovery attribution
Wichmann et al. (2022) [64]	Initial AIS and later AIS conversion; Principal SCI n=15	After multiple-testing correction, plasma GDNF associated with conversion (p=0.017); no interchangeable absolute concentration threshold. CSF GDNF omitted owing to >90% below detection.	Confidence interval not reported; Four withdrawals; n=11 outcome; low-detection exclusions and multiple tests; few events
Stukas et al. (2023) [51]	AIS conversion, motor-complete status, >8-point total motor gain; sensory/motor ZPP; Principal SCI n=118	For initially AIS A n=74, 72-h serum NfL ≥264 pg/mL non-conversion sensitivity 76% (95% CI 61–87), specificity 77% (55–92). For motor recovery in cervical SCI n=65, NfL ≥170 pg/mL ≤8-point gain sensitivity 88% (69–97), specificity 91% (75–98). Sensory ZPP improved model accuracy.	Thresholds internally derived; not clinical treatment-selection rules; Assay saturation (GFAP above upper range); selected examinable non-polytrauma patients; internal cross-validation, no external validation
Wichmann et al. (2023) [65]	Baseline AIS, later AIS conversion and SCIM-III; Principal SCI n=15	GFAP associated with initial AIS (CSF p=0.024; peripheral blood p=0.030). No marker associated significantly with later AIS conversion or SCIM-III. Serum NSE excluded due to haemolysis.	Confidence interval not reported; 11 complete follow-up, multiple comparisons, ceiling-censored GFAP/NfL values; no adjusted prognostic model
Yang et al. (2023) [52]	Initial AIS and discharge AIS improvement; Principal SCI n=15	No CSF biomarker differentiated AIS improvement; serum day-2 tau was the exception among mainly null recovery comparisons. SCI-versus-control diagnostic AUCs do not establish recovery prediction.	Confidence interval not reported; 15 selected durotomy patients, four recovery events; numerous tests; figure AIS A n=11 contradicts 9 in patient table

Table S9. Quantitative and null findings with analysis-specific interpretation			
**Report**	**Clinical endpoint and analytical sample**	**Reported finding and uncertainty**	**Interpretation or reporting limitation**
Agulló-Ortuño et al. (2024) [42]	6MWT, 10MWT, WISCI-II, BBS and TUG; Principal SCI n=40	No between-sequence concentration differences at individual assessment times. Period-change comparisons differed for IL-1β/IL-4/IL-12p70 during combined therapy and TNF-α/IL-4/IL-12p70/IL-1β/MCP-1 during usual therapy. IL-8 fell in both groups; late-sequence baseline-to-week-12 ΔIL-8 versus Δ10MWT Pearson r=−0.776, p=0.003; pairwise n not reported.	40 analysed biomarkers, functional tests have smaller denominators; 20% excluded from complete biomarker analysis; no washout amid evolving injury; differing functional denominators; correlation selection and multiplicity
Ding et al. (2024) [21]	One-year AIS A–C versus D–E; initial AIS; BASIC/IMLL/MCC/MSCC; Principal SCI n=374	Among n=374 (147 AIS A–C/227 AIS D–E at one year), CRP AUC 0.833 (95% CI 0.792–0.874); BASIC AUC 0.958 (95% CI 0.939–0.976). The combined BASIC–CRP model had an apparent AUC of 0.973 (95% CI 0.959–0.987).	n=374; univariate CRP–one-year AIS r=−0.472, p<0.001; adjusted OR coding not interpreted owing to reporting ambiguity; Matrix/platform unresolved; complete-case selection; table OR/CI inconsistencies; no external calibration/validation
Haddadi et al. (2024) [53]	Complete versus incomplete AIS status; Principal SCI n=31	In n=31 (nine complete/22 incomplete injuries), serum ROC AUC=0.586 (cut-off 14.70 ng/dL, sensitivity 66.7%, specificity 55%); CSF AUC=0.823 (342.18 ng/dL, sensitivity 100%, specificity 64%). Numerical AUC uncertainty not reported. Serum–CSF correlation r=0.070, p=0.709.	ROC confidence intervals not reported; No recovery endpoint; small n, incomplete CSF assay description, discordant group-test/correlation reporting; data-derived cut-offs
Pu et al. (2024) [20]	GCS severity and GOS global outcome/survival; Principal SCI n=60	Combined NSE/neuritin sensitivity 100%, specificity 97.62% reported, without CIs or independent validation. GOS is a global neurotrauma outcome, not SCI-specific motor recovery.	Do not interpret neuritin as NfL/NfH or transport diagnostic performance to isolated SCI; Major brain-injury confounding; neuritin/neurofilament terminology conflict; OR/beta inconsistencies; units and assessment horizon uncertain
Azad et al. (2025) [54]	AIS severity and 6-month conversion; Principal SCI n=50	cfDNA detected 39/50 SCI and 0/20 controls. In n=34, four proteins plus cfDNA injury index conversion AUC 0.77, p=0.006 (18 converters/16 non-converters).	Model AUC CI not reported; 50 enrolled is not model denominator; Subset selected by available plasma; predictor selection and performance in same sample; mixed aetiology; no external validation
Fraussen et al. (2025) [55]	AIS conversion and ≥5-point motor-score improvement; Principal SCI n=37	Baseline GFAP non-conversion OR per 10,000 pg/mL 3.22 (95% CI 1.32–17.52); NfL per 100 pg/mL 1.47 (1.02–2.96). Three-marker conversion AUC 0.863 (SE 0.067; 95% CI 0.731–0.994; reported groups 13 converters/20 non-converters). ≥5-point motor-gain AUC 0.857 (SE 0.076; 95% CI 0.707–1.000; reported groups 15 gain/14 below threshold). Separate complete-case ROC n not stated; no cross-validation.	Reported motor-score availability is internally inconsistent (27 at baseline versus 29 in the motor-change groups); separate complete-case ROC denominators are not stated. Motor-gain prediction at later samples differs from baseline severity classification; varying outcome time, few events, exploratory pairwise tests without global correction, and no cross-validation.
Morrison et al. (2025) [66]	Initial motor completeness and immune phenotype; Principal SCI n=36	CRP and selected inflammatory mediators associated with initial motor completeness. No prospective recovery effect reported here. Assay-specific n: ELISA 35, RNA 36, flow 14.	Confidence interval not reported; Sample availability, polytrauma and timing confounding; modality-specific n; no recovery inference

Table S9. Quantitative and null findings with analysis-specific interpretation			
**Report**	**Clinical endpoint and analytical sample**	**Reported finding and uncertainty**	**Interpretation or reporting limitation**
Raheja et al. (2025) [56]	Baseline AIS and 6-month AIS-defined motor status; Principal SCI n=26	Day-2 GFAP severity AUC 0.77 (95% CI 0.56–0.98). Day-7 S100B six-month AIS A–C status AUC 0.86 (0.58–1.00); complete-injury AUC 1.00 in 9 survivors followed.	Initial n=26, follow-up n=9; not all biomarker assays necessarily complete; 10 deaths and 7 lost; recovery analyses only 9; perfect discrimination severely unstable; many exploratory time/marker contrasts
Seijas et al. (2025) [19]	Baseline serum androgens; Rasch-transformed SCIM-III trajectory/discharge; principal SCI n=80 (65 men/15 women)	Fully adjusted discharge association per 1 SD: DHEAS 0.2% (95% CI −24 to 20), p=0.99; DHEA 2.5% (−17 to 19), p=0.79. Free testosterone 26.9% (9 to 42), p=0.01, is contextual. Adrenal androgen findings varied by subgroup.	Percentages use a reflected/log-transformed functioning model, not absolute SCIM points. Clinical missingness imputed under MAR; early discharge changes visit availability. Neurological severity not included in the displayed fully adjusted discharge model; wide female uncertainty; ≥16 allowed; possible SwiSCI overlap
Vedantam et al. (2025) [67]	mJOA, NDI, QuickDASH, BBS, SF36-PCS, grip/pinch strength and MRI; Principal SCI n=20	NfL+IL-6+BDNF diagnostic AUC 0.83 (95% CI 0.68–0.98). Presurgical NfL associated with pinch improvement p=0.03; BDNF rose after surgery p=0.001. Increase alone is not independently measured plasticity.	20 clinical follow-up; two missing baseline strength, one missing NfL, some cytokines excluded below quantification; n=20, stepwise many-predictor analyses; no full comorbidity capture; assay missingness/censoring; no external validation
Waliullah et al. (2025) [57]	AIS severity groups over 6 weeks; Principal SCI n=26	The primary report's baseline table gives 0.166±0.016 versus 0.028±0.02 μg/L, p=0.025; groups no longer differ at 6 weeks (p=0.637). Narrative baseline 0.95±0.16 conflicts with table values.	Table values used with explicit discrepancy; CIs not reported; Numerical inconsistencies; no recovery-change regression, unclear multiplicity and missingness; no validated prognostic threshold
Belogurov et al. (2026) [68]	Karnofsky status at discharge, tumour length and surgical injury; MEP/D-wave used intraoperatively; Principal SCI n=8	Human cytokines tracked surgical injury; CSF TNF-α, IL-4 and CXCL10 inversely associated with discharge Karnofsky score. Pharmacological locomotor benefits were demonstrated in rodents only.	Human correlations exploratory; coefficients/CIs not fully supplied in text; Eight selected tumour patients, high-dose perioperative corticosteroids, uncertain association n/CI; no human drug trial or biomarker–MEP recovery analysis
Hei et al. (2026) [58]	AIS ≥1-grade conversion, MRI lesion length/cord compression; Principal SCI n=71	38 converters and 33 non-converters (n=71). Adjusted tau OR 0.996 (95% CI 0.993–1.000), p=0.028; GFAP/CNTN1 not independently significant. Apparent admission AIS+IMLL+tau AUC 0.936 (95% CI 0.871–1.000).	n=71; charted as serum according to the collection and assay Methods; conflicting plasma terminology in the title and Results limits specimen-specific interpretation; 6-month/1-year wording inconsistent; GFAP significance contradictory; stepwise fit and no external validation

Table S9. Quantitative and null findings with analysis-specific interpretation

Report	Clinical endpoint and analytical sample	Reported finding and uncertainty	Interpretation or reporting limitation
Sheinberg et al. (2026) [59]	AIS conversion, motor scores and BASIC MRI; in-vitro neural-cell effects separate; Principal SCI n=25	Five-protein initial-severity AUC 0.854; four-protein improvement panel (MIF/HGF/IL-1RA/IL-10) AUC 0.813. Numerical AUC uncertainty not reported; fitted-model n not clearly specified. Figure 2B source data include 62 specimens from 25 patients (16 improvement/9 non-improvement), despite the severe-group caption. No internal/external protein-model validation. One miRNA qPCR finding confirmed in an independent 10-patient sample set.	25 patients; 38 quality-controlled RNA specimens; separate qPCR 17 specimens from 10 patients; Few subjects and high-dimensional selection; repeated specimens; inconsistent ≥1 versus >1 AIS improvement definition; cell-culture effects are not human plasticity

Note: CIs, SEs and SEMs retain their reported meaning. Confidence intervals are reported only when available in the original publications. Model- or endpoint-specific sample sizes may be smaller than the enrolled sample. Kwon 2017 and Fraussen 2025 provide both SEs and numerical 95% CIs for the highlighted panel AUCs. Waliullah reports conflicting baseline values in the narrative and table. The table values are presented with this discrepancy noted. Todd's printed CI column cannot be interpreted as an interval for the reported standardized effect. Hei is classified as serum according to its collection and assay Methods; conflicting plasma terminology in the title and Results and inconsistent outcome wording remain reporting limitations. Prognostic performance of a biomarker panel does not establish validation of every constituent analyte.

Abbreviations: SCI, spinal cord injury; TBI, traumatic brain injury; AIS, American Spinal Injury Association Impairment Scale; ASIA, American Spinal Injury Association; ISNCSCI, International Standards for Neurological Classification of Spinal Cord Injury; CSF, cerebrospinal fluid; CRP, C-reactive protein; IL, interleukin; IL-1ra, interleukin-1 receptor antagonist; IL-1RA, interleukin-1 receptor antagonist; IL-6, interleukin-6; IL-8, interleukin-8; IL-10, interleukin-10; IL-13, interleukin-13; TNF-α, tumour necrosis factor alpha; BDNF, brain-derived neurotrophic factor; NGF, nerve growth factor; GDNF, glial cell line-derived neurotrophic factor; GFAP, glial fibrillary acidic protein; NSE, neuron-specific enolase; NfL, neurofilament light chain; NfH, neurofilament heavy chain; pNF-H, phosphorylated neurofilament heavy chain; S100B, S100 calcium-binding protein B; CNTN1, contactin-1; PTH, parathyroid hormone; SEM, standard error of the mean; SE, standard error; SD, standard deviation; CI, confidence interval; AUC, area under the curve; ROC, receiver operating characteristic; OR, odds ratio; BMI, body mass index; HPA, hypothalamic–pituitary–adrenal; DHEAS, dehydroepiandrosterone sulfate; MRI, magnetic resonance imaging; BASIC, Brain and Spinal Injury Center; IMLL, intramedullary lesion length; SCIM, Spinal Cord Independence Measure; SCIM-III, Spinal Cord Independence Measure version III; FIM, Functional Independence Measure; JOA, Japanese Orthopaedic Association score; mJOA, modified Japanese Orthopaedic Association score; JOACMEQ, Japanese Orthopaedic Association Cervical Myelopathy Evaluation Questionnaire; EQ-5D, EuroQol five-dimension instrument; GOS, Glasgow Outcome Scale; NP, neuropathic pain; NRS, numerical rating scale; 10MWT, 10-metre walk test; IFN-γ, interferon gamma; PGE2, prostaglandin E2; ELISA, enzyme-linked immunosorbent assay; FDR, false discovery rate; RNA, ribonucleic acid; miRNA, microRNA; qPCR, quantitative polymerase chain reaction; cfDNA, cell-free deoxyribonucleic acid; MEP, motor evoked potential; ZPP, zone of partial preservation; MIF, macrophage migration inhibitory factor; HGF, hepatocyte growth factor; CXCL10, C-X-C motif chemokine ligand 10; GCS, Glasgow Coma Scale; CES-D, Center for Epidemiologic Studies Depression Scale; LANSS, Leeds Assessment of Neuropathic Symptoms and Signs; MCC, maximum canal compromise; MSCC, maximum spinal cord compression; NDI, Neck Disability Index; SF36-PCS, 36-Item Short Form Health Survey physical component summary; QuickDASH, shortened Disabilities of the Arm, Shoulder and Hand questionnaire; WISCI-II, Walking Index for Spinal Cord Injury version II; BBS, Berg Balance Scale; TUG, Timed Up and Go; 6MWT, 6-minute walk test; IL-2, interleukin-2; IL-4, interleukin-4.

Table S10a. Result-specific critical-appraisal criteria

Criterion	Information examined	Interpretive consequence
Participation	Recruitment, clinical selection, eligibility, aetiology, level/AIS, controls and residual-function requirements.	Selected phenotype limits applicability; same-cohort reports do not constitute replication.
Attrition	Enrolled, assayed, paired and endpoint-specific numbers; deaths, withdrawals and losses.	Complete-case or survivor analyses may distort prognosis; unequal biomarker/function denominators restrict comparison.
Biomarker measurement	Matrix, platform, handling, units, timing, blinding, detection limits, haemolysis and imputation.	Concentration/threshold transport requires matrix/assay comparability; measurement inconsistency can alter estimated associations.
Clinical endpoint	Instrument, baseline versus future status versus change, follow-up timing and assessor blinding.	Initial severity is not recovery; total scores and affective pain components cannot establish separate cognition/mood outcomes.
Confounding	Baseline AIS/motor score, age, aetiology, co-injury, infection, medication, surgery and actual treatment exposure.	Unadjusted associations do not establish incremental prognosis or biological attribution.
Analysis/reporting	Outcome-specific n, model complexity, multiplicity, resampling, calibration, external validation and result-selection safeguards.	Development AUC may be optimistic; exploratory correlations do not establish causal mediation or differential treatment benefit.
Randomized contrast	RoB 2 randomization, deviations, missing data, endpoint measurement and reported-result selection for a defined result/time.	Appraisal applies to the assigned contrast; biomarkers themselves remain observational exposures.

Note: These extraction criteria provide context. Formal assessments use the result-specific tools in S10b–S10k, with their own domains and decision rules. No common numerical quality score or cross-design certainty grade is assigned. Small samples primarily limit precision; selection, unhandled missingness, confounding and data-driven model evaluation may additionally introduce bias.

Abbreviations: AIS, American Spinal Injury Association Impairment Scale; AUC, area under the curve; RoB 2, revised Cochrane risk-of-bias tool for randomized trials.

Table S10b. RoB 2 judgments for specified randomized results							
**Report**	**Assigned contrast, outcome and time**	**Randomization**	**Deviations**	**Missing outcome data**	**Outcome measurement**	**Reported-result selection**	**Overall**
Allison et al. (2016) [38]	Diet/supplements vs usual diet; sensory NPQ pain, 12 weeks	SC: random allocation stated; concealment insufficiently described	SC: unblinded participants; stable pain drugs; adherence measured	LR: 20/20 completed endpoint	HR: subjective pain reported by unblinded participants	SC: analysis-plan/result-selection detail insufficient	HR
Allison et al. (2016) [38]	Diet/supplements vs usual diet; IL-6/CRP change, 12 weeks	SC: same allocation limitation	SC: diet/co-interventions and adherence	LR: all 20 completed; biomarker-specific missingness not highlighted	LR: laboratory outcomes; examiner blinded	SC: multiple outcomes; insufficient prespecified analysis detail	SC
Agulló-Ortuño et al. (2024) [42]	Early vs late aquatic sequence; cytokines, weeks 6/12	SC: software-generated random sequence; concealment unclear	SC: open sequence; usual therapy continues; assignment analysis incompletely documented	SC: 40/50 complete biomarker datasets; arm-specific missingness and sensitivity analysis insufficient	LR: laboratory multiplex outcomes	SC: multiplicity correction for some cytokine tests; prospective result selection unclear	SC*
Agulló-Ortuño et al. (2024) [42]	Early vs late aquatic sequence; 10MWT, weeks 6/12	SC: same sequence limitation	SC: usual therapy continues; delivered dose and assignment analysis incompletely documented	SC: functional denominators vary by test/time; influence of missingness unresolved	SC: performance test; assessment blinding insufficiently described	SC: multiple functional/time-point tests	HR*

Note: result-specific judgments for the effect of treatment assignment. Allison uses the parallel-trial RoB 2 version (22 August 2019); the repeated-period Agulló-Ortuño results also require crossover Domain S (18 March 2021), reported in S10b2. *Overall includes Domain S. Missingness is not automatically high risk because a percentage threshold is exceeded. Overall HR follows a high-risk domain; otherwise SC reflects unresolved concerns. The cytokine panel groups endpoints with common assessment conditions; the judgment concerns the reported panel comparison and does not imply that every secondary analyte effect was separately appraised. Observational biomarker correlations in these trials are not randomized effects. Disagreements concerning appraisal judgments were resolved through discussion or adjudication.

Abbreviations: CRP, C-reactive protein; IL, interleukin; IL-6, interleukin-6; 10MWT, 10-metre walk test; NPQ, Neuropathic Pain Questionnaire; RoB 2, revised Cochrane risk-of-bias tool for randomized trials; SC, some concerns; HR, high risk; LR, low risk.

Table S10b2. RoB 2 crossover Domain S for the repeated-period results			
**Report and assessed result**	**S.1 / S.2 / S.3**	**Domain S**	**Reason**
Agulló-Ortuño et al. (2024) [42] Cytokine comparisons across the six- and twelve-week periods	NI / NI / NI	SC	Initial allocated numbers per sequence and period-adjusted analysis are insufficiently reported; 21/19 are completer counts. Six-week periods follow immediately. It is unclear whether cytokine effects have disappeared during the later UT-only period. No washout alone does not establish high risk.
Agulló-Ortuño et al. (2024) [42]; 10MWT current-treatment interpretation across both periods	NI / NI / PN	HR	Training-related functional gains are plausibly sustained into the UT-only period, rather than dissipating before week 12; the published repeated-period comparison cannot securely isolate a current-treatment effect. S.3 PN is a content-based judgment, with direction unpredictable.
Note: S.1 concerns sequence balance; S.2 concerns adjustment for period effects when required; S.3 concerns dissipation of carryover. NI, no information; PN, probably no. These codes follow the crossover tool algorithm: NI for S.3 yields SC; N/PN yields HR. The judgment concerns both-period current-treatment inference. A separately defined first-period randomized contrast would require a separate assessment and is not interchangeable with these entries.			

Abbreviations: 10MWT, 10-metre walk test; RoB 2, revised Cochrane risk-of-bias tool for randomized trials; SC, some concerns; HR, high risk; NI, no information; PN, probably no.

**Table S10c. Primary appraisal allocation across the 41 reports**			
**Primary question/design**	**Tool and version**	**Reports**	**Location**
Randomized intervention assignment	RoB 2 parallel (2019); crossover (2021)	2	S10b and S10b2: Allison; Agulló-Ortuño
Baseline prognostic factor and later outcome	QUIPS (Hayden et al., 2013)	14	S10d
Developed multivariable prediction and its performance	PROBAST+AI (2025), development and evaluation sections	10	S10e–S10f
Concurrent analytical phenotype association	JBI analytical cross-sectional (2026)	6	S10g
Case-control physiological comparison	JBI case-control (official checklist)	1	S10h
Descriptive longitudinal trajectory	JBI cohort (official checklist)	4	S10i
Descriptive case series	JBI case series (official checklist)	2	S10j
Uncontrolled before–after intervention	Revised JBI quasi-experimental checklist (2023; methods publication 2024)	2	S10k
Controlled intervention comparison without randomization	ROBINS-I; no eligible primary contrast identified	0	Not applied to these reports
Total primary report allocations	2 randomized + 39 nonrandomized	41	Each nonrandomized report appears once in S10d/e/g/h/i/j/k
Tool selection. QUIPS is used for a baseline biomarker–future outcome association, including individual-factor ROC exploration. PROBAST+AI is used for developed combinations intended to predict an individual outcome, even when the algorithm is ordinary regression. JBI design-specific tools address concurrent phenotype associations, descriptive trajectories, case-control comparisons, case series and uncontrolled before–after experiments. No report in the primary nonrandomized appraisal set estimates a controlled nonrandomized intervention effect requiring ROBINS-I. Concurrent diagnostic/severity ROC results are not treated as evidence of future recovery; a synthesis of diagnostic test accuracy would require a separately defined accuracy appraisal.			
Appraisal units. The corpus comprises 41 reports, with two randomized intervention reports and 39 nonrandomized reports. For allocation summaries, a developed multivariable model intended for individual-outcome prediction was selected when present, followed by an early biomarker–later outcome association; otherwise the principal concurrent, descriptive or intervention question in S9 determined the instrument. Selection was based on the relationship relevant to the review, rather than statistical significance or effect size. Grouped results share the stated analytical setting; not every secondary estimate was separately assessed with a formal instrument. A focused structured assessment of material secondary associations is provided in S10m and does not alter the once-per-report allocation. Kwon 2010 is an observational marker-model analysis within a randomized trial. Laboratory randomization of samples and random order of questionnaires do not make Fraussen, Streijger or Todd randomized intervention trials. Report counts do not count independent cohorts; dependencies remain in S6c.			
Interpretation. QUIPS uses low (L), moderate (M) or high (H) risk per domain; no invented aggregate rule is used. PROBAST+AI uses L/H/U (unclear) for development-quality concerns, evaluation risk of bias and applicability separately. An H domain produces an H overall concern/risk; when none is H, unresolved information produces U. JBI uses yes (Y), no (N), unclear (U) and not applicable (NA) per criterion, without numerical totals or arbitrary low/moderate/high cutoffs. Reporting-only JBI items are not automatically internal-bias domains. Small n is not a stand-alone high-risk criterion; candidate model complexity, selection and uncorrected apparent performance may create optimism in addition to imprecision.			
Tables S10c1–S10c8 display the assessments for every included report. The domain and criterion judgments reproduce the detailed assessments in S10b–S10k. Green denotes low risk or a criterion answered Yes; yellow denotes moderate risk, some concerns or unclear information; red denotes high risk or a criterion answered No; grey denotes not applicable. The written judgment governs interpretation.			

Abbreviations: ROC, receiver operating characteristic; JBI, JBI critical-appraisal instruments; RoB 2, revised Cochrane risk-of-bias tool for randomized trials; QUIPS, Quality in Prognosis Studies; NA, not applicable; PROBAST+AI, Prediction model Risk Of Bias ASsessment Tool with updated provisions for regression and artificial intelligence methods; L, low risk; M, moderate risk; H, high risk; Y, yes; U, unclear; ROBINS-I, Risk Of Bias In Non-randomized Studies of Interventions. Response codes retain their instrument-specific meanings.

Table S10c1. RoB 2 summary for two randomized reports and four specified results

Report	Assessed result	D1	D2	D3	D4	D5	Domain S	Overall
Allison et al. (2016) [38]	Diet/supplements vs usual diet; sensory NPQ pain, 12 weeks	Some concerns	Some concerns	Low risk	High risk	Some concerns	NA	High risk
Allison et al. (2016) [38]	Diet/supplements vs usual diet; IL-6/CRP change, 12 weeks	Some concerns	Some concerns	Low risk	Low risk	Some concerns	NA	Some concerns
Agulló-Ortuño et al. (2024) [42]	Early vs late aquatic sequence; cytokines, weeks 6/12	Some concerns	Some concerns	Some concerns	Low risk	Some concerns	Some concerns	Some concerns
Agulló-Ortuño et al. (2024) [42]	Early vs late aquatic sequence; 10MWT, weeks 6/12	Some concerns	Some concerns	Some concerns	Some concerns	Some concerns	High risk	High risk

D1, randomization; D2, deviations from intended interventions; D3, missing outcome data; D4, outcome measurement; D5, selection of the reported result. Domain S concerns period/carryover effects in crossover trials and is NA for Allison. Overall includes Domain S for Agulló-Ortuño. The four rows represent result-specific assessments from two reports. Reasons and crossover signalling answers are in S10b and S10b2.

Abbreviations: CRP, C-reactive protein; IL, interleukin; IL-6, interleukin-6; 10MWT, 10-metre walk test; NPQ, Neuropathic Pain Questionnaire; RoB 2, revised Cochrane risk-of-bias tool for randomized trials; NA, not applicable. LR, low risk; SC, some concerns; HR, high risk; NA, not applicable.

**Table S10c2. QUIPS risk of bias by domain for 14 prognostic-factor reports**						
**Report**	**Study participation**	**Attrition**	**Prognostic factor measurement**	**Outcome measurement**	**Confounding**	**Analysis / reporting**
Hayakawa et al. (2012) [43]	Moderate	Moderate	Moderate	Moderate	High	High
Pouw et al. (2014) [44]	Moderate	Low	Low	Moderate	High	Moderate
Takahashi et al. (2014) [60]	Moderate	Moderate	Low	Moderate	High	Moderate
Kato et al. (2015) [61]	Low	Moderate	Moderate	Moderate	High	Moderate
Du et al. (2018) [48]	Moderate	Low	Moderate	Moderate	High	High
Rieder et al. (2019) [49]	Moderate	High	Low	Moderate	High	Moderate
Capirossi et al. (2020) [50]	Low	High	Moderate	Moderate	High	High
Holmström et al. (2020) [62]	Low	Moderate	Moderate	Moderate	High	High
Wichmann et al. (2022) [64]	Moderate	High	Moderate	Moderate	High	Moderate
Wichmann et al. (2023) [65]	Moderate	High	Moderate	Moderate	High	Moderate
Yang et al. (2023) [52]	Moderate	Low	Moderate	Moderate	High	Moderate
Raheja et al. (2025) [56]	Moderate	High	Low	Moderate	High	High
Seijas et al. (2025) [19]	Moderate	Moderate	Moderate	Moderate	High	Moderate
Vedantam et al. (2025) [67]	Moderate	Moderate	Moderate	Moderate	High	High
The labels indicate risk of bias in each domain. No overall QUIPS score or cross-design quality grade is assigned. Factor–outcome pairs, analytical denominators, reasons are in S10d.						

Abbreviations: QUIPS, Quality in Prognosis Studies. Response codes retain their instrument-specific meanings. QUIPS: L, low; M, moderate; H, high risk.

Table S10c3. PROBAST+AI assessments for 10 prediction-model reports

Report	D1 Participants	D2 Predictors	D3 Outcome	D4 Analysis	Overall quality concern	Overall risk of bias	Applicability P / Pr / O
Kwon et al. (2010) [33]	Unclear	Unclear	Unclear	High	High	High	U / L / L
Kwon et al. (2017) [34]	Unclear	Unclear	Unclear	High	High	High	U / L / L
Streijger et al. (2017) [47]	Unclear	Low	Unclear	High	High	High	U / L / L
Stukas et al. (2023) [51]	Unclear	Unclear	Unclear	High	High	High	U / L / L
Ding et al. (2024) [21]	Unclear	Unclear	Unclear	High	High	High	U / U / L
Pu et al. (2024) [20]	High	Unclear	High	High	High	High	H / U / H
Azad et al. (2025) [54]	Unclear	Unclear	Unclear	High	High	High	U / L / L
Fraussen et al. (2025) [55]	Unclear	Low	High	High	High	High	U / L / U
Hei et al. (2026) [58]	Unclear	Unclear	Unclear	High	High	High	U / U / U
Sheinberg et al. (2026) [59]	Unclear	Low	High	High	High	High	U / L / U

The D1–D4 profiles are the documented domain judgments for both development quality and evaluation risk of bias. Their underlying analysis questions are assessed separately in S10f. High concern in D4 produces high overall development-quality concern and evaluation risk of bias; reasons are in S10e. Applicability is separate: P, participants; Pr, predictors; O, outcome; L, low; U, unclear; H, high. These entries assess the specified prognostic models, with analytical samples and outcomes defined in S10e.

Abbreviations: PROBAST+AI, Prediction model Risk Of Bias ASsessment Tool with updated provisions for regression and artificial intelligence methods; L, low risk; H, high risk; U, unclear. Response codes retain their instrument-specific meanings. PROBAST+AI: L, low; H, high; U, unclear.

Table S10c4. JBI analytical cross-sectional assessments of six reports									
**Report**	**Q1**	**Q2**	**Q3**	**Q4**	**Q5**	**Q6**	**Q7**	**Q8**	**Evidence table**
Mechanick et al. (1997) [35]	Unclear	Yes	Yes	Yes	Yes	Unclear	Unclear	Yes	S10g
Morse et al. (2008) [36]	Yes	Yes	Unclear	Yes	Yes	Yes	Unclear	Yes	S10g
Goldstein et al. (2017) [39]	Yes	Yes	Unclear	Yes	Yes	Yes	Yes	Yes	S10g
Ahadi et al. (2015) [17]	Yes	Yes	Yes	Unclear	Yes	No	Unclear	Yes	S10g
Haddadi et al. (2024) [53]	Yes	Yes	Yes	Unclear	Yes	No	Yes	Yes	S10g
Morrison et al. (2025) [66]	Yes	Yes	Yes	Yes	Yes	No	Yes	Yes	S10g

Figure 3, Panel C. Q1, eligibility; Q2, condition criteria; Q3, exposure measurement; Q4, outcome measurement; Q5, confounders identified; Q6, confounding strategy; Q7, analysis; Q8, population/setting description. 2026 JBI item order. Q8 is a reporting criterion.

Yes, criterion met; No, criterion not met; Unclear, insufficient information; NA, not applicable. These are item-level critical-appraisal responses. No percentage score or arbitrary overall low/moderate/high category is assigned. Appraisal units and supporting reasons are provided in S10g.

Abbreviations: JBI, JBI critical-appraisal instruments; NA, not applicable. Response codes retain their instrument-specific meanings. JBI: Y, yes; N, no; U, unclear; NA, not applicable.

**Table S10c5. JBI case-control assessment of one report**											
**Report**	**Q1**	**Q2**	**Q3**	**Q4**	**Q5**	**Q6**	**Q7**	**Q8**	**Q9**	**Q10**	**Evidence table**
Cuce et al. (2019) [40]	Unclear	No	Yes	Yes	Yes	Yes	No	Yes	Unclear	Yes	S10h
Figure 3, Panel D. Q1, group comparability; Q2, matching; Q3, common identification criteria; Q4, exposure validity; Q5, consistent exposure measurement; Q6, confounders identified; Q7, confounding strategy; Q8, outcome measurement; Q9, meaningful exposure interval; Q10, analysis.											
Yes, criterion met; No, criterion not met; Unclear, insufficient information; NA, not applicable. These are item-level critical-appraisal responses. No percentage score or arbitrary overall low/moderate/high category is assigned. Appraisal units and supporting reasons are provided in S10h.											

Abbreviations: JBI, JBI critical-appraisal instruments; NA, not applicable. Response codes retain their instrument-specific meanings. JBI: Y, yes; N, no; U, unclear; NA, not applicable.

Table S10c6. JBI cohort assessments of four longitudinal reports

**Report**	**Q1**	**Q2**	**Q3**	**Q4**	**Q5**	**Q6**	**Q7**	**Q8**	**Q9**	**Q10**	**Q11**	**Evidence table**
Singh et al. (2017) [46]	Yes	Yes	Yes	Yes	No	NA	Yes	Yes	Unclear	Unclear	Unclear	S10i
Lin et al. (2021) [63]	Yes	Yes	Yes	Yes	Unclear	NA	Yes	Yes	Unclear	Yes	Yes	S10i
Itodo et al. (2022) [18]	Yes	Yes	Yes	Yes	Yes	NA	Unclear	Yes	Unclear	Unclear	Yes	S10i
Waliullah et al. (2025) [57]	Yes	Yes	Yes	Yes	No	NA	Unclear	Yes	Unclear	Unclear	Unclear	S10i

Figure 3, Panel E. Q1, group/source comparability; Q2, consistent exposure classification; Q3, exposure validity; Q4, confounders identified; Q5, confounding strategy; Q6, outcome-free baseline; Q7, outcome measurement; Q8, follow-up horizon; Q9, completeness/reasons for loss; Q10, incomplete-follow-up strategy; Q11, analysis. Q6 is NA for these existing-SCI trajectory questions.

Yes, criterion met; No, criterion not met; Unclear, insufficient information; NA, not applicable. These are item-level critical-appraisal responses. No percentage score or arbitrary overall low/moderate/high category is assigned. Appraisal units and supporting reasons are provided in S10i.

Abbreviations: SCI, spinal cord injury; JBI, JBI critical-appraisal instruments; NA, not applicable. Response codes retain their instrument-specific meanings. JBI: Y, yes; N, no; U, unclear; NA, not applicable.

Table S10c7. JBI case-series assessments of two reports

Report	Q1	Q2	Q3	Q4	Q5	Q6	Q7	Q8	Q9	Q10	Evidence table
Ungureanu et al. (2014) [45]	Unclear	Yes	Yes	Unclear	Unclear	Yes	Yes	Unclear	Unclear	Unclear	S10j
Belogurov et al. (2026) [68]	Yes	Yes	Yes	Unclear	Unclear	Yes	Yes	Unclear	Yes	No	S10j

Figure 3, Panel F. Q1, eligibility; Q2, standardized condition measurement; Q3, valid case identification; Q4, consecutive inclusion; Q5, complete inclusion; Q6, demographics; Q7, clinical information; Q8, outcomes/follow-up reporting; Q9, site/clinic description; Q10, analysis.

Yes, criterion met; No, criterion not met; Unclear, insufficient information; NA, not applicable. These are item-level critical-appraisal responses. No percentage score or arbitrary overall low/moderate/high category is assigned. Appraisal units and supporting reasons are provided in S10j.

Abbreviations: JBI, JBI critical-appraisal instruments; NA, not applicable. Response codes retain their instrument-specific meanings. JBI: Y, yes; N, no; U, unclear; NA, not applicable.

Table S10c8. JBI quasi-experimental assessments of two before–after reports

Report	Q1	Q2	Q3	Q4	Q5	Q6	Q7	Q8	Q9	Evidence table
Harness et al. (2014) [37]	Yes	No	NA	NA	No	Yes	Yes	Unclear	Yes	S10k
Todd et al. (2021) [41]	Yes	No	NA	NA	No	Yes	Unclear	Unclear	Unclear	S10k

Figure 3, Panel G. Q1, temporal order; Q2, control group; Q3, participant comparability; Q4, comparable other care; Q5, multiple measurements before and after; Q6, consistent outcome assessment; Q7, reliable outcomes; Q8, follow-up completeness/handling; Q9, analysis. Q3–4 are NA for these single-cohort contrasts; Q5 requires multiple pre- and post-intervention measurements.

Yes, criterion met; No, criterion not met; Unclear, insufficient information; NA, not applicable. These are item-level critical-appraisal responses. No percentage score or arbitrary overall low/moderate/high category is assigned. Appraisal units and supporting reasons are provided in S10k.

Abbreviations: JBI, JBI critical-appraisal instruments; NA, not applicable. Response codes retain their instrument-specific meanings. JBI: Y, yes; N, no; U, unclear; NA, not applicable.

Table S10d. QUIPS domain judgments for the 14 prognostic-factor reports								
**Report**	**Factor–outcome appraisal unit**	**P**	**A**	**F**	**O**	**C**	**S**	**Domain rationale**
Hayakawa et al. (2012) [43]	Early plasma pNF-H and final AIS status; n=14; follow-up 43–556 days	M	M	M	M	H	H	Participation: recruitment flow insufficiently detailed. Attrition: serial draw availability varies. Factor: ELISA specified, but below-detection values set to zero. Outcome: standard AIS with markedly variable horizon. Confounding: initial severity and sampling time not adequately isolated. Analysis: unadjusted serial subgroup comparisons do not establish incremental prognosis.
Pouw et al. (2014) [44]	≤24-hour CSF proteins and later AIS/conversion; n=16, NfH n=15; 6 or 12 months	M	L	L	M	H	M	Participation: consenting, examineable early-surgery cohort; 16/23 screened included under reported criteria. Attrition: later clinical assessments reported for the included cohort; one insufficient NfH specimen. Factor: timed CSF and specified assays. Outcome: trained ISNCSCI assessment but two horizons. Confounding: severity-stratified descriptive comparisons without adequate adjustment. Analysis: appropriate simple comparisons, with very small strata and limited result-selection safeguards.
Takahashi et al. (2014) [60]	Preoperative CSF pNF-H and six-month JOA/recovery after decompression; analysed n=14	M	M	L	M	H	M	Participation/attrition: the text initially mentions 15 myelopathy patients but reports groups of 8 and 6; disposition is unclear. Factor: standard preoperative ELISA. Outcome: JOA assessed by two surgeons; blinding not reported. Confounding: disease course, baseline deficit and surgery not jointly adjusted. Analysis: rank correlations are appropriate; this is an exploratory null recovery association, without a prespecified prognostic analysis.
Kato et al. (2015) [61]	Preoperative detectable serum pNF-H and three-month mJOA recovery; n=26, detectable n=6	L	M	M	M	H	M	Participation: prospective consecutive surgical recruitment. Attrition: clinical recovery is reported, but its exact denominator is not explicitly separated from postoperative blood availability (18/26). Factor: specified assay and threshold; serum/plasma wording inconsistent. Outcome: standard mJOA, assessor blinding unclear. Confounding: baseline mJOA and disease duration not adjusted in the detectable/non-detectable recovery contrast. Analysis: exploratory group comparison; no independent prognostic effect established.
Du et al. (2018) [48]	Early serum NSE/S100B and ≥1-grade AIS improvement at six months; n=60 (21/39)	M	L	M	M	H	H	Participation: selected acute cohort with limited recruitment-flow information. Attrition: all 60 assigned to reported outcome groups. Factor: same-batch assays, but group labels/concentrations conflict within the text. Outcome: AIS improvement specified; blinding unclear. Confounding: major baseline motor/sensory differences between outcome groups are unadjusted. Analysis: outcome-derived cutoffs and exploratory serial tests increase optimism and selection risk; these are individual-factor results.

Table S10d. QUIPS domain judgments for the 14 prognostic-factor reports								
**Report**	**Factor–outcome appraisal unit**	**P**	**A**	**F**	**O**	**C**	**S**	**Domain rationale**
Rieder et al. (2019) [49]	Early serum markers and six-month AIS/FIM; recruited n=52; endpoint-specific n incompletely reported	M	H	L	M	H	M	Participation: isolated, conscious ICU SCI sample. Attrition: 19% mortality, only 16 paired early samples, and unclear six-month analyte-specific denominators; survivor selection cannot be resolved. Factor: specified duplicate assays. Outcome: established scales, blinding unclear. Confounding: severity, critical illness and treatment not adequately adjusted for the marker–outcome associations. Analysis: conventional correlations but incomplete analytical denominators and multiplicity safeguards.
Capirossi et al. (2020) [50]	Early CSF panel and discharge SCIM/motor scores or change; 9/11 analysed; discharge 47–320 days	L	H	M	M	H	H	Participation: consecutive recruitment with explicit eligibility. Attrition: death and contaminated CSF excluded from the analysed cohort; unavailable marker values not imputed. Factor: multiplex assay described, with selective detectability. Outcome: trained assessment, variable discharge horizon. Confounding: baseline impairment, co-injury and rehabilitation not adjusted. Analysis: many marker–scale correlations in nine patients, selected by magnitude, without adequate multiplicity protection.
Holmström et al. (2020) [62]	Preoperative CSF proteins and three-month clinical/questionnaire outcome after untethering; 12 patients, 15 operations	L	M	M	M	H	H	Participation: consecutive acquired-tethering cohort. Attrition: result-specific postoperative denominators unclear. Factor: specified assays; CSF collection differs by comparator. Outcome: standard instruments with telephone questionnaire follow-up and unclear blinding. Confounding: aetiology, baseline deficit and surgical course not jointly adjusted. Analysis: repeated operations are not independent patients; the simple correlation/comparison strategy does not clearly handle this dependence or multiplicity.
Wichmann et al. (2022) [64]	Early CSF/blood proteins and later AIS conversion; 11/15 followed, 4 conversions	M	H	M	M	H	M	Participation: selected acute, alert SCI patients. Attrition: four consent withdrawals; outcome-related differences and sensitivity analyses not documented. Factor: Olink procedures specified; GDNF below detection in CSF, so this result cannot be used. Outcome: AIS assessed in the chronic phase; blinding unclear. Confounding: baseline severity/level not adequately controlled for conversion. Analysis: multiple-testing adjustment is reported; sparse events leave the exploratory association vulnerable to residual selection and imprecision.

Table S10d. QUIPS domain judgments for the 14 prognostic-factor reports								
**Report**	**Factor–outcome appraisal unit**	**P**	**A**	**F**	**O**	**C**	**S**	**Domain rationale**
Wichmann et al. (2023) [65]	Early structural/inflammatory markers and later AIS conversion/SCIM; same cohort, 11/15 followed	M	H	M	M	H	M	Participation/attrition: same selected cohort and four withdrawals as the 2022 report; this is not replication. Factor: standardized platforms and haemolysis exclusions, but range capping/imputation can affect estimates. Outcome: trained AIS/SCIM assessment; blinding unclear. Confounding: baseline impairment/level not adequately resolved for future outcome. Analysis: regression and model checks are described, but clinical-outcome analyses are sparse and exploratory; later-outcome null findings are retained.
Yang et al. (2023) [52]	Early CSF/serum panel and discharge AIS improvement; n=15 AIS A/B, 4 improvers; <3 months	M	L	M	M	H	M	Participation: surgical/CSF-drain eligibility specified, but eligible-patient flow and participation proportion insufficiently documented. The restricted phenotype separately limits applicability. Attrition: all 15 completed the reported study. Factor: specified panels, but sampling is linked to drainage/surgery. Outcome: variable discharge timing and inconsistent initial-group numbers in figure text. Confounding: baseline deficit, surgery and drain selection not adjusted for improvement. Analysis: adjusted multiple comparisons are reported, but only four events and principally null improvement tests support no validated prognosis.
Raheja et al. (2025) [56]	Early serum markers and six-month AIS status; 9/26 assessed; 10 deaths and 7 losses	M	H	L	M	H	H	Participation: selected adults without head injury/polytrauma. Attrition: the prognosis contrast is conditional on nine followed survivors; seven losses and competing deaths are not adequately handled. Factor: timed serum ELISAs/storage specified. Outcome: later AIS status is distinct from grade conversion; blinding unclear. Confounding: initial injury severity not adequately adjusted. Analysis: data-derived cutoffs and selected subgroup ROC results, including perfect discrimination, are highly optimistic and lack validation.
Seijas et al. (2025) [19]	Baseline DHEA/DHEAS and later Rasch-transformed SCIM-III functioning during rehabilitation; n=80	M	M	M	M	H	M	Participation: selected consenting biobank participants with paired serum samples, 80/97 assessed. Attrition: 11 unpaired specimens excluded; missing clinical data imputed under MAR, with early discharge altering visit availability. Factor: ELISA and paired same-plate processing specified, but 07:00–14:00 sampling and matching of circadian timing remain concerns. Outcome: trained professionals used validated SCIM-III/Rasch scores, but blinding to hormone results is not specified. Confounding: the displayed fully adjusted discharge model includes age, sex and health variables, but not baseline neurological severity or initial functioning. Analysis: mixed models, CIs, imputation and Bonferroni sensitivity are reported; sparse sex/subgroup estimates and the transformed outcome constrain interpretation.

Table S10d. QUIPS domain judgments for the 14 prognostic-factor reports								
**Report**	**Factor–outcome appraisal unit**	**P**	**A**	**F**	**O**	**C**	**S**	**Domain rationale**
Vedantam et al. (2025) [67]	Preoperative serum proteins and three-month pinch/function change after DCM surgery; n=20, result-specific n smaller	M	M	M	M	H	H	Participation: prospective selected surgical DCM cohort. Attrition: all have three-month follow-up, but strength and biomarker data are missing for some tests. Factor: specified assays; below-quantification values excluded/extrapolated and matrix wording inconsistent. Outcome: standard functional measures, blinding unclear. Confounding: the small association models do not adequately establish independence from baseline deficit/comorbidity. Analysis: numerous exploratory marker/function regressions without sufficient multiplicity or missing-data safeguards; no individual prediction model is claimed here.

QUIPS domains. P, study participation; A, attrition; F, prognostic-factor measurement; O, outcome measurement; C, confounding; S, statistical analysis/reporting. The domains are interpreted for the stated factor–outcome relationship, not for diagnostic comparisons with healthy controls.

Note: These are domain judgments, not a validated overall QUIPS score or a certainty-of-evidence grade. Future AIS status, AIS conversion and motor-score change remain distinct outcomes. A null result does not lower or raise risk of bias.

Abbreviations: SCI, spinal cord injury; AIS, American Spinal Injury Association Impairment Scale; ISNCSCI, International Standards for Neurological Classification of Spinal Cord Injury; CSF, cerebrospinal fluid; GDNF, glial cell line-derived neurotrophic factor; NSE, neuron-specific enolase; NfH, neurofilament heavy chain; pNF-H, phosphorylated neurofilament heavy chain; S100B, S100 calcium-binding protein B; ROC, receiver operating characteristic; SCIM, Spinal Cord Independence Measure; FIM, Functional Independence Measure; JOA, Japanese Orthopaedic Association score; mJOA, modified Japanese Orthopaedic Association score; ELISA, enzyme-linked immunosorbent assay; QUIPS, Quality in Prognosis Studies; DCM, degenerative cervical myelopathy; ICU, intensive care unit; L, low risk; M, moderate risk; H, high risk; P, study participation; A, attrition; F, prognostic-factor measurement; O, outcome measurement; C, confounding; S, statistical analysis and reporting. Response codes retain their instrument-specific meanings. QUIPS: L, low; M, moderate; H, high risk.

Table S10e. PROBAST+AI domain profiles and reasons for the 10 prediction-model reports						
**Report**	**Index model and outcome**	**Quality D1–D4**	**Bias D1–D4**	**Appl. P / Pr / O**	**Overall**	**Rationale**
Kwon et al. (2010) [33]	CSF S100B/GFAP/IL-8 model for six-month segmental upper-extremity motor recovery; cervical n=20	U / U / U / H	U / U / U / H	U / L / L	Q H B H Ap U	Participants: selected examineable acute trial cohort; the analysed marker model is observational, not a randomized treatment contrast. Predictors/outcome: specified timed assays and neurological testing; blinding incompletely reported. Analysis: bootstrap modelling reported, but optimism correction and the complete prognostic pipeline are insufficiently specified; effective sample is small relative to candidate selection. The 18/9 split in the article concerns initial-severity classification, not this motor-recovery model. Prognostic calibration and external validation are absent.
Kwon et al. (2017) [34]	CSF inflammatory/structural indices for six-month AIS conversion; 48/50 assessed	U / U / U / H	U / U / U / H	U / L / L	Q H; B H; Ap U	Participants: selected acute AIS A–C cohort; one death and one loss at six months. Predictors/outcome: specified assays/ISNCSCI, assessor blinding unclear. Analysis: indices are derived after bivariate comparisons; subgroup logistic/LDA models and leave-one-out validation reported. Whether all index construction/selection was repeated within resampling is unclear. Hosmer–Lemeshow testing alone does not adequately establish calibration; small outcome strata and no external validation limit transport.
Streijger et al. (2017) [47]	24-hour CSF sparse PLS model for six-month total motor-score change; 27/29 assessed	U / L / U / H	U / L / U / H	U / L / L	Q H; B H; Ap U	Participants: selected early acute AIS A–C cohort. Predictors: randomized assay plates, blinded quality review and explicit LLOQ handling. Outcome: six-month ISNCSCI, blinding unclear. Analysis: sparse modelling and leave-one-out tuning mitigate overfitting, but 165 targets and 27 outcomes make model selection unstable. The description does not establish a separate nested loop repeating all tuning/preprocessing. Cross-validated R^2 is reported; calibration/prediction-error assessment is insufficient. External evaluation is absent.
Stukas et al. (2023) [51]	Early serum NF-L/GFAP combination for six-month AIS conversion within baseline AIS A; outcome n=74	U / U / U / H	U / U / U / H	U / L / L	Q H; B H; Ap U	Participants: pooled acute cohorts; 113/118 have follow-up, but conversion model uses the AIS A subset. Predictors/outcome: specified Simoa assays and ISNCSCI; some values exceed assay limits and blinding incompletely described. Analysis: leave-one-out LDA evaluation reported, alongside several sampling times, outcomes and biomarker/clinical combinations. Full pipeline selection within resampling and missing-data strategy are unclear. Effective conversion events, repeated model comparisons and inadequate calibration create high analytical concern. Programme overlap is separately recorded in S6c.

**Table S10e. PROBAST+AI domain profiles and reasons for the 10 prediction-model reports**						
**Report**	**Index model and outcome**	**Quality D1–D4**	**Bias D1–D4**	**Appl. P / Pr / O**	**Overall**	**Rationale**
Ding et al. (2024) [21]	CRP+BASIC logistic model for one-year AIS status; retrospective cervical SCI n=374	U / U / U / H	U / U / U / H	U / U / L	Q H; B H; Ap U	Participants: retrospective surgical cervical cohort, with data/follow-up selection not fully resolved. Predictors: CRP matrix/platform unspecified; MRI and clinical predictors described. Outcome: future AIS status, not necessarily conversion; blinding unclear. Analysis: univariable screening followed by logistic modelling, with apparent ROC performance on development data. No optimism correction/shrinkage or adequate calibration assessment is reported; coding/OR direction affects inference. Larger n alone does not remove these analytical limitations.
Pu et al. (2024) [20]	Combined NSE+neuritin prognostic classification; SCI plus TBI n=60; GOS groups 42/11/7	H / U / H / H	H / U / H / H	H / U / H	Q H; B H; Ap H	Participants: all have combined TBI/SCI; stated clear-consciousness eligibility conflicts with very low reported GCS. Predictors: neuritin is repeatedly called neurofilament and matrix/timing/model specification are unclear. Outcome: GOS horizon and dichotomization insufficiently specified, and global brain/cord outcome cannot isolate SCI recovery. Analysis: many candidates, only 18 poor/deceased outcomes, nonreproducible combined-rule specification and apparent performance without validation/calibration. These limitations preclude clinical interpretation of near-perfect accuracy.
Azad et al. (2025) [54]	SCII multi-analyte model for six-month AIS conversion; proteomic prognosis n=34 (18/16)	U / U / U / H	U / U / U / H	U / L / L	Q H; B H; Ap U	Participants: mixed traumatic/compressive cord injury, with only 34 in the proteomic prognosis comparison. Predictors: cfDNA/ddPCR and NULISA assays described; blinding unclear. Outcome: six-month conversion, distinct from diagnostic SCI/non-SCI classification. Analysis: proteins ranked by observed outcome differences and forward-selected, then combined with cfDNA in the same cohort. No resampling/optimism correction or adequate calibration is reported; effective n is inadequate for the broad candidate search. Non-SCI controls do not externally validate this prognostic model.
Fraussen et al. (2025) [55]	Baseline NfL+GFAP+CNTN1 logistic combinations for AIS conversion (reported groups n=33) and ≥5-point motor gain (reported groups n=29). Complete-case ROC n unstated.	U / L / H / H	U / L / H / H	U / L / U	Q H; B H; Ap U	Participants: 37 initially, 34 endpoint records; result-specific subsets differ. Predictors: blinded duplicate assays with batch bridging. Outcome: latest available assessment rather than a uniform prediction horizon; assessor blinding unclear. Analysis: multiple single/combined marker logistic/ROC comparisons in small event subsets; cross-validation explicitly not performed and calibration not adequately reported. Mixed-model MAR handling for biomarker trajectories does not establish adequate missing-data handling for the separate complete-case prediction analyses.

Table S10e. PROBAST+AI domain profiles and reasons for the 10 prediction-model reports

Report	Index model and outcome	Quality D1–D4	Bias D1–D4	Appl. P / Pr / O	Overall	Rationale
Hei et al. (2026) [58]	Admission AIS+IMLL+tau logistic model for later AIS conversion; n=71 (38/33)	U / U / U / H	U / U / U / H	U / U / U	Q H; B H; Ap U	Participants: single-centre cervical cohort; exclusions for incomplete records and inability to assess AIS may select prognosis. Predictors: blinded duplicate MRI ratings; the specimen is charted as serum according to the collection and assay Methods; conflicting plasma terminology in the title and Results remains a reporting limitation. Outcome: Methods specify six months after discharge, while other wording differs; outpatient/telephone ascertainment and blinding need clarification. Analysis: p<0.10 candidate screening and apparent ROC evaluation, without optimism correction or adequate calibration. Candidate selection and missing-record handling are insufficiently documented.
Sheinberg et al. (2026) [59]	CSF exosomal MIF/HGF/IL-1RA/IL-10 panel for later AIS improvement; 25 patients, model subgroup n unclear	U / L / H / H	U / L / H / H	U / L / U	Q H; B H; Ap U	Participants: 25 patients; 38 CSF RNA specimens do not define the protein-model n. Predictors: blinded duplicate protein assays, with patient-level averaging described. Outcome: ≥1 versus >1-grade improvement wording conflicts; exact model subgroup/event counts are unclear. Analysis: proteins and optimal combinations selected by ROC in the discovery data; no internal/external protein-model validation or adequate calibration. The separate ten-patient qPCR set validates one miRNA measurement, not the four-protein prognostic model.

Prediction assessment. All ten entries involve model development plus apparent/internal performance evaluation; none constitutes an independent external evaluation of the stated prognostic model. D1, participants/data sources; D2, predictors; D3, outcome; D4, analysis. Development quality and evaluation bias use the same first-three-domain profiles because the assessed model uses the same cohort and measurement process; analysis is appraised separately using the appropriate questions. Applicability domains are participants (P), predictors (Pr) and outcome (O). Overall entries report quality concern (Q), evaluation bias (B) and applicability concern (Ap).

Table S10e. PROBAST+AI domain profiles and reasons for the 10 prediction-model reports

Report	Index model and outcome	Quality D1–D4	Bias D1–D4	Appl. P / Pr / O	Overall	Rationale
PROBAST+AI intended use. Population: adults in the corresponding traumatic, compressive or perioperative SCI stratum, with exceptions explicitly flagged. Index: the specified early fluid-marker combination, sometimes with baseline clinical/MRI data. Comparator: the clinical/severity or individual-marker comparator reported, when available. Outcomes: later neurological status or improvement, analysed distinctly. Timing: acute measurement before follow-up, usually three to twelve months; uncertainty in the study horizon is flagged. Setting/use: prognosis after acute SCI or cord surgery, not selection of a biomarker-guided treatment. Applicability judgments concern this review purpose and do not certify transport to other SCI strata or laboratories.						
Note: Every model has high analytical concern in this assessment, so overall development quality concern and performance bias are H; this conclusion does not depend solely on small sample size or absence of external validation. Internal validation can be adequate when it repeats the entire development pipeline. Here the reported safeguards do not adequately resolve the specified selection/optimism/calibration concerns. Applicability is a separate judgment. Fraussen AIS conversion and motor-gain models share this profile; event numbers and thresholds are kept separate. Neither Pu global GOS nor an initial-severity classifier establishes SCI-specific recovery prediction.						

Abbreviations: SCI, spinal cord injury; TBI, traumatic brain injury; AIS, American Spinal Injury Association Impairment Scale; ISNCSCI, International Standards for Neurological Classification of Spinal Cord Injury; CSF, cerebrospinal fluid; CRP, C-reactive protein; IL, interleukin; IL-1RA, interleukin-1 receptor antagonist; IL-8, interleukin-8; IL-10, interleukin-10; GFAP, glial fibrillary acidic protein; NSE, neuron-specific enolase; NfL, neurofilament light chain; S100B, S100 calcium-binding protein B; CNTN1, contactin-1; ROC, receiver operating characteristic; OR, odds ratio; MRI, magnetic resonance imaging; BASIC, Brain and Spinal Injury Center; IMLL, intramedullary lesion length; GOS, Glasgow Outcome Scale; Simoa, single molecule array; LLOQ, lower limit of quantification; miRNA, microRNA; qPCR, quantitative polymerase chain reaction; ddPCR, droplet digital polymerase chain reaction; cfDNA, cell-free deoxyribonucleic acid; MIF, macrophage migration inhibitory factor; HGF, hepatocyte growth factor; GCS, Glasgow Coma Scale; NULISA, nucleic acid-linked immunosandwich assay; PROBAST+AI, Prediction model Risk Of Bias ASsessment Tool with updated provisions for regression and artificial intelligence methods; L, low risk; H, high risk; U, unclear; MAR, missing at random; LDA, linear discriminant analysis; PLS, partial least squares; NF-L, neurofilament light chain; Q, development quality; B, evaluation risk of bias; Ap, applicability; P, participants; Pr, predictors; O, outcome. Response codes retain their instrument-specific meanings. PROBAST+AI: L, low; H, high; U, unclear.

**Table S10f. PROBAST+AI signalling-question answers for the specified models**					
**Report**	**D1 1.1–1.3**	**D2 2.1–2.4**	**D3 3.1–3.4**	**Development D4 4.1–4.5**	**Evaluation D4 4.1–4.7**
Kwon et al. (2010) [33]	Y / Y / NI	Y / PY / NI / Y	Y / Y / NI / Y	PN / NI / NI / NA / PY	NI / PN / NI / NA / NA / NI / PN
Kwon et al. (2017) [34]	Y / Y / NI	Y / Y / NI / Y	Y / Y / NI / Y	PN / PY / NI / NA / PN	PY / PN / NI / NA / NA / NI / PN
Streijger et al. (2017) [47]	Y / Y / NI	Y / Y / PY / Y	Y / Y / NI / Y	PN / PY / NI / NA / Y	Y / PN / NI / NA / NA / NI / PN
Stukas et al. (2023) [51]	Y / Y / NI	Y / Y / NI / Y	Y / Y / NI / Y	PN / PY / NI / NA / PN	Y / PN / NI / NA / NA / NI / PN
Ding et al. (2024) [21]	PY / Y / NI	NI / NI / NI / Y	Y / Y / NI / Y	NI / PY / NI / NA / N	N / NI / NI / NA / NA / NA / PN
Pu et al. (2024) [20]	PY / Y / PN	NI / NI / NI / NI	PN / NI / NI / NI	PN / NI / NI / NA / N	N / PN / NI / NA / NA / NA / N
Azad et al. (2025) [54]	Y / Y / NI	Y / Y / NI / Y	Y / Y / NI / Y	PN / PY / NI / NA / N	N / PN / NI / NA / NA / NA / PN
Fraussen et al. (2025) [55]	Y / Y / NI	Y / Y / Y / Y	Y / PN / NI / PN	PN / PY / NI / NA / N	N / PN / NI / NA / NA / NA / PN
Hei et al. (2026) [58]	Y / Y / NI	Y / PY / NI / Y	Y / NI / NI / NI	PN / NI / NI / NA / N	N / PN / NI / NA / NA / NA / PN
Sheinberg et al. (2026) [59]	Y / Y / NI	Y / Y / Y / Y	PN / NI / NI / PY	PN / PY / NI / NA / N	N / PN / NI / NA / NA / NA / PN

Code order. Each slash-separated answer follows the ascending question numbers in its header. Y, yes; PY, probably yes; PN, probably no; N, no; NI, no information; NA, not applicable. D1–D3 answers apply to both development and internal evaluation. NI is distinct from N. Conditional NA is used where no class-imbalance correction, train/test splitting or resampling is reported for the assessed model; the initial-severity split in Kwon 2010 is not assigned to its prognosis model.

Question labels (paraphrased). D1: data source, design, representativeness. D2: predictor measurement, preprocessing consistency, outcome-blind assessment, availability at intended use. D3: outcome definition, consistency, predictor-blind assessment, interval. Development D4: sample adequacy for candidate complexity, predictor handling, missing/censored data, recalibration after imbalance correction, overfitting safeguards. Evaluation D4: avoid apparent-only performance, evaluation sample adequacy, missing/censored data, evaluate original prevalence after imbalance correction, prevent splitting leakage, repeat all pipeline steps within resampling, assess predictive performance adequately. Reasons are in S10e.

Abbreviations: NA, not applicable; PROBAST+AI, Prediction model Risk Of Bias ASsessment Tool with updated provisions for regression and artificial intelligence methods; Y, yes; NI, no information; PN, probably no; PY, probably yes. Response codes retain their instrument-specific meanings. Y, yes; PY, probably yes; PN, probably no; N, no; NI, no information; NA, not applicable.

Table S10g. JBI analytical cross-sectional appraisal of six reports										
**Report**	**Appraisal unit**	**1**	**2**	**3**	**4**	**5**	**6**	**7**	**8**	**Criterion rationale**
Mechanick et al. (1997) [35]	Concurrent serum cortisol and thyroid measures versus SCI severity/completeness/level; SCI n=49; separate TBI comparators n=33	U	Y	Y	Y	Y	U	U	Y	Q1 U: retrospective, clinically selected inpatient metabolic-bone evaluation; complete recruitment criteria/record availability unclear. Q2–4 Y: ASIA severity and clinical serum endocrine tests specified. Q5 Y: age and illness differences identified. Q6 U: adjusted attenuation is reported for PTH/1,25-vitamin D, not separately established for the qualifying cortisol/thyroid comparisons. Q7 U: tests are described, but target-specific effect sizes, uncertainty and analytical denominators are insufficiently reported. Q8 Y: setting and clinical groups described. PTH findings are contextual and do not independently establish eligibility or recovery prognosis.
Morse et al. (2008) [36]	Concurrent plasma hsCRP and habitual mobility; analysed n=63 men	Y	Y	U	Y	Y	Y	U	Y	Q3 U: standardized self-reported mobility, but independent validity/reliability not established. Q4 Y: specified hsCRP assay. Q5–6 Y: adiposity/illness addressed in regression. Q7 U: excluded CRP outlier and conflicting mobility counts limit inference; variable screening may leave residual confounding. Q1–2/8 Y: eligibility, SCI definition and setting reported. Concurrent association cannot establish direction.
Goldstein et al. (2017) [39]	Concurrent plasma hsCRP and mobility/adiposity; analysed n=338/360	Y	Y	U	Y	Y	Y	Y	Y	Q3 U: self-reported mobility/illness measures without sufficient validation detail. Q4 Y: specified hsCRP assay. Q5–7 Y: predefined illness considerations and five adjusted adiposity models, with transformation/multiple-comparison handling. Q1–2/8 Y: exclusions and population described. Residual confounding and complete-case selection remain; 26 participants overlap with Morse, and one adjusted mobility model is null.
Ahadi et al. (2015) [17]	Early serum GFAP/NSE/pNF-H and initial severity; SCI n=26, fracture controls n=9	Y	Y	Y	U	Y	N	U	Y	Q4 U: analyte-specific denominators, figure labels and text significance are inconsistent. Q5 Y: co-brain injury and severity are considered in selection/descriptions. Q6 N: clinical severity, surgery/critical illness and timing are not adequately jointly controlled. Q7 U: inconsistencies limit interpretation of serial group comparisons. Q1–3/8 Y: selection, ASIA/CT definitions and setting reported. Death exclusions/early deaths complicate completeness; the overall trauma cohort included participants aged 16, but SCI-specific age composition was not separately reported.

**Table S10g. JBI analytical cross-sectional appraisal of six reports**										
**Report**	**Appraisal unit**	**1**	**2**	**3**	**4**	**5**	**6**	**7**	**8**	**Criterion rationale**
Haddadi et al. (2024) [53]	Serum/CSF S100B and initial complete versus incomplete SCI; n=31 (9/22)	Y	Y	Y	U	Y	N	Y	Y	Q4 U: serum platform described more clearly than the CSF assay and sampling comparability. Q5 Y: demographic/co-injury variables considered. Q6 N: key severity/timing differences not adequately adjusted; nonsignificant demographic tests do not establish no confounding. Q7 Y: nonparametric group/ROC analyses fit the stated concurrent comparison, although uncertainty and cutoff optimism remain. Q1–3/8 Y: eligibility, ASIA groups and setting described; no future recovery endpoint.
Morrison et al. (2025) [66]	Plasma immune markers and initial AIS A/B versus C/D; SCI n=36, ELISA n=35	Y	Y	Y	Y	Y	N	Y	Y	Q1–4/8 Y: eligibility, ISNCSCI grouping, assay quality control and setting described. Q5 Y: concomitant trauma/injury burden measured. Q6 N: completeness-associated inflammation is not adequately separated from co-injury burden. Q7 Y: group tests with FDR/Dunn procedures described. Different assay denominators (ELISA/RNA/flow) restrict cross-assay inference; initial completeness is a concurrent phenotype.
JBI analytical cross-sectional tool 2026. Q1, eligibility; Q2, condition criteria; Q3, exposure measurement; Q4, outcome measurement; Q5, confounders identified; Q6, confounding strategy; Q7, analysis; Q8, population/setting description. Q8 concerns reporting and is not an internal-bias domain. Mobility/AIS phenotype is treated as exposure and the laboratory marker as the concurrently measured outcome for these comparisons.										
Note: Columns 1–8 correspond to Q1–Q8 above. item-level critical appraisal; Y, yes; N, no; U, unclear; NA, not applicable. No summed quality score, arbitrary risk category or exclusion decision is assigned. Reasons specify unmet/unclear criteria; affirmative items indicate reported safeguards, not proof that all bias has been removed.										

Abbreviations: SCI, spinal cord injury; AIS, American Spinal Injury Association Impairment Scale; ASIA, American Spinal Injury Association; ISNCSCI, International Standards for Neurological Classification of Spinal Cord Injury; CSF, cerebrospinal fluid; CRP, C-reactive protein; hsCRP, high-sensitivity C-reactive protein; GFAP, glial fibrillary acidic protein; NSE, neuron-specific enolase; pNF-H, phosphorylated neurofilament heavy chain; S100B, S100 calcium-binding protein B; PTH, parathyroid hormone; ROC, receiver operating characteristic; ELISA, enzyme-linked immunosorbent assay; FDR, false discovery rate; RNA, ribonucleic acid; JBI, JBI critical-appraisal instruments; NA, not applicable; N, no; H, high risk; Y, yes; U, unclear; CT, computed tomography. Response codes retain their instrument-specific meanings. JBI: Y, yes; N, no; U, unclear; NA, not applicable.

Table S10h. JBI case-control appraisal of one report

Report	Appraisal unit	1	2	3	4	5	6	7	8	9	10	Criterion rationale
Cuce et al. (2019) [40]	SCI with neuropathic pain (15) versus without pain (7); ACTH/cortisol responses; healthy controls (10) secondary	U	N	Y	Y	Y	Y	N	Y	U	Y	Q1 U/Q2 N: the pain/no-pain SCI groups are not matched for lesion phenotype/duration; age/sex matching pertains to healthy controls. Q3–5 Y: consistent SCI eligibility and standardized morning hormone challenges. Q6 Y/Q7 N: potential age/sex, lesion and drug influences identified but not adequately jointly adjusted. Q8 Y: defined neuropathic pain/standard scales. Q9 U: concurrent physiology does not establish exposure before pain onset. Q10 Y: rank correlations/group tests appropriate for an exploratory concurrent comparison.

JBI case-control checklist. Q1, group comparability; Q2, matching; Q3, common identification criteria; Q4, exposure validity; Q5, consistent exposure measurement; Q6, confounders identified; Q7, confounding strategy; Q8, outcome measurement; Q9, meaningful exposure interval; Q10, analysis. The primary comparison here is neuropathic pain versus no pain within SCI, rather than healthy volunteers.

Note: Columns 1–10 correspond to Q1–Q10 above. item-level critical appraisal; Y, yes; N, no; U, unclear; NA, not applicable. No summed quality score, arbitrary risk category or exclusion decision is assigned. Reasons specify unmet/unclear criteria; affirmative items indicate reported safeguards, not proof that all bias has been removed.

Abbreviations: SCI, spinal cord injury; ACTH, adrenocorticotropic hormone; JBI, JBI critical-appraisal instruments; NA, not applicable; N, no; Y, yes; U, unclear. Response codes retain their instrument-specific meanings. JBI: Y, yes; N, no; U, unclear; NA, not applicable.

Table S10i. JBI cohort appraisal of four descriptive longitudinal reports

Report	Appraisal unit	1	2	3	4	5	6	7	8	9	10	11	Criterion rationale
Singh et al. (2017) [46]	Serial plasma pNF-H and contemporaneous AIS over 90 days; SCI n=28	Y	Y	Y	Y	N	NA	Y	Y	U	U	U	Q1–3 Y: severity groups drawn from one defined cohort, using common AIS/ELISA procedures. Q4 Y/Q5 N: severity/treatment selection described, without an adequate confounding strategy. Q6 NA: trajectories concern existing SCI, not incident disease. Q7–8 Y: repeated established measures over the stated acute horizon. Q9–10 U: variable serial specimen availability and missing-data handling incompletely explained. Q11 U: inconsistent peak-day/summary reporting limits interpretation; numeric AIS summaries do not establish adjusted change prognosis.
Lin et al. (2021) [63]	Genotype/severity strata and serial inflammatory/pain trajectories; SCI n=45	Y	Y	Y	Y	U	NA	Y	Y	U	Y	Y	Q1–4 Y: common prospective cohort, specified genotype/plasma procedures and clinical severity variables. Q5 U: severity stratification and genotype/time terms address some influences; adequacy for age/medication/co-injury is unclear. Q6 NA: existing SCI and repeated symptoms. Q7–8 Y: consistent scales and serial timing. Q9 U: endpoint-specific follow-up completeness not fully clear. Q10–11 Y: 20-dataset multiple imputation and Rubin pooling described; the MAR assumption remains uncertain, and a nonsignificant MCAR test does not prove random missingness.
Itodo et al. (2022) [18]	Serum androgen trajectories during inpatient rehabilitation by injury phenotype; n=86	Y	Y	Y	Y	Y	NA	U	Y	U	U	Y	Q1–5 Y: common rehabilitation cohort, standardized injury strata and adjustment for age/BMI/level/completeness/drugs. Q6 NA: endocrine trajectories after existing SCI. Q7 U: blood obtained between 07:00 and 14:00 without sufficiently documented matched circadian timing. Q8 Y: admission/discharge horizon fits the endocrine question. Q9–10 U: 14 missing baseline neurological records and endpoint-specific complete-case handling require clarification. Q11 Y: paired/mixed modelling described. Recruitment permitted age >16; no neurological-recovery linkage is established.

Table S10i. JBI cohort appraisal of four descriptive longitudinal reports

Report	Appraisal unit	1	2	3	4	5	6	7	8	9	10	11	Criterion rationale
Waliullah et al. (2025) [57]	Serial serum S100B by initial SCI completeness at baseline, two and six weeks; cases n=26	Y	Y	Y	Y	N	NA	U	Y	U	U	U	Q1–4 Y: common cohort, specified AIS/ELISA and recorded clinical variables. Q5 N: severity/management influences not adequately adjusted. Q6 NA: existing SCI biomarker trajectories. Q7 U/Q11 U: serum concentration/serial significance statements conflict within the report, limiting interpretation of summaries and comparisons. Q8 Y: six weeks fits the acute trajectory question. Q9–10 U: serial clinical/specimen completeness and missing-data handling insufficiently reported.

JBI cohort checklist. Q1, group/source comparability; Q2, consistent exposure classification; Q3, exposure validity; Q4, confounders identified; Q5, confounding strategy; Q6, outcome-free baseline; Q7, outcome measurement; Q8, follow-up horizon; Q9, completeness/reasons for loss; Q10, incomplete-follow-up strategy; Q11, analysis. Exposure strata are baseline injury phenotype or genotype; outcomes are serial marker/symptom trajectories. Q6 is NA because these questions concern existing SCI and repeated states, not new incident disease. These NA judgments are explicit contextual decisions, not affirmative safeguards.

Note: Columns 1–11 correspond to Q1–Q11 above. item-level critical appraisal; Y, yes; N, no; U, unclear; NA, not applicable. No summed quality score, arbitrary risk category or exclusion decision is assigned. Reasons specify unmet/unclear criteria; affirmative items indicate reported safeguards, not proof that all bias has been removed.

Abbreviations: SCI, spinal cord injury; AIS, American Spinal Injury Association Impairment Scale; pNF-H, phosphorylated neurofilament heavy chain; S100B, S100 calcium-binding protein B; BMI, body mass index; ELISA, enzyme-linked immunosorbent assay; JBI, JBI critical-appraisal instruments; NA, not applicable; N, no; MCAR, missing completely at random; H, high risk; Y, yes; U, unclear; MAR, missing at random. Response codes retain their instrument-specific meanings. JBI: Y, yes; N, no; U, unclear; NA, not applicable.

Table S10j. JBI case-series appraisal of two reports												
**Report**	**Appraisal unit**	**1**	**2**	**3**	**4**	**5**	**6**	**7**	**8**	**9**	**10**	**Criterion rationale**
Ungureanu et al. (2014) [45]	Daily CSF pNF-H and descriptive neurological evolution; acute SCI n=15	U	Y	Y	U	U	Y	Y	U	U	U	Q1 U: detailed eligibility incompletely specified. Q2–3 Y: ASIA and imaging/assay procedures described. Q4–5 U: consecutive and complete inclusion not established. Q6–7 Y: demographic/injury and operative details reported. Q8 U: early death, variable serial draws and clinical horizons not fully reconciled. Q9 U: recruitment-site population insufficiently characterized. Q10 U: statistical strategy for biomarker–evolution claims insufficiently specified. These descriptive patterns are not an adjusted prognostic effect.
Belogurov et al. (2026) [68]	Human perioperative CSF/plasma cytokines and discharge Karnofsky; 8/10 tumour patients retained	Y	Y	Y	U	U	Y	Y	U	Y	N	Q1–3 Y: defined tumour/surgery eligibility and histological classification; two different histologies excluded. Q4–5 U: consecutive/complete eligible recruitment not established. Q6–7/9 Y: patient/tumour, treatment and single-centre details reported. Q8 U: human correlation-specific n/follow-up completeness unclear. Q10 N: numerous human marker/clinical correlations without adequate multiplicity protection or clear analytical denominators. Corticosteroids and surgery qualify attribution. Drug intervention results are from rodents, not these patients.

JBI case-series checklist. Q1, eligibility; Q2, standardized condition measurement; Q3, valid case identification; Q4, consecutive inclusion; Q5, complete inclusion; Q6, demographics; Q7, clinical information; Q8, outcomes/follow-up reporting; Q9, site/clinic description; Q10, analysis. Reporting items are interpreted as reporting/completeness criteria, without conversion into a pooled internal-bias score.

Note: Columns 1–10 correspond to Q1–Q10 above. item-level critical appraisal; Y, yes; N, no; U, unclear; NA, not applicable. No summed quality score, arbitrary risk category or exclusion decision is assigned. Reasons specify unmet/unclear criteria; affirmative items indicate reported safeguards, not proof that all bias has been removed.

Abbreviations: SCI, spinal cord injury; ASIA, American Spinal Injury Association; CSF, cerebrospinal fluid; pNF-H, phosphorylated neurofilament heavy chain; JBI, JBI critical-appraisal instruments; NA, not applicable; N, no; H, high risk; Y, yes; U, unclear. Response codes retain their instrument-specific meanings. JBI: Y, yes; N, no; U, unclear; NA, not applicable.

Table S10k. JBI quasi-experimental appraisal of two uncontrolled before–after reports											
**Report**	**Appraisal unit**	**1**	**2**	**3**	**4**	**5**	**6**	**7**	**8**	**9**	**Criterion rationale**
Harness et al. (2014) [37]	Single activity-based exercise bout and immediate serum BDNF/cortisol; n=15, cortisol sequence n=12	Y	N	NA	NA	N	Y	Y	U	Y	Q1 Y: exercise precedes the acute post-bout measurement. Q2 N: no independent control. Q3–4 NA: one cohort, not separate intervention/control groups. Q5 N: only one pre-bout baseline despite multiple post-modality samples. Q6–7 Y: same sampling/assay procedures. Q8 U: cortisol-specific missingness (12/15) not sufficiently resolved. Q9 Y: repeated-measures testing described. Fixed exercise order, time-of-day drift and carryover prevent isolation of modality effects; this is not sustained rehabilitation recovery.
Todd et al. (2021) [41]	Single arm-crank bout and immediate/90-minute neuropathic pain/inflammation; 10 enrolled, 8 analysed	Y	N	NA	NA	N	Y	U	U	U	Q1 Y: intervention before post-bout outcomes. Q2 N: no independent control. Q3–4 NA: single cohort. Q5 N: one pre-exercise baseline. Q6 Y: common assessment sequence. Q7 U: modified instantaneous self-report scale without blinded assessment; objective assays separately described. Q8 U: two exclusions and absent sensitivity assessment leave effect-related completeness unclear. Q9 U: exploratory post-hoc marker/pain correlations and ambiguous printed interval column; achieved intensity differs by lesion group. Acute affect/pain is not a training-course recovery endpoint.

Revised JBI quasi-experimental checklist, 2023 version (methods publication 2024). Q1, temporal order; Q2, control group; Q3, participant comparability; Q4, comparable other care; Q5, multiple measurements before and after; Q6, consistent outcome assessment; Q7, reliable outcomes; Q8, follow-up completeness/handling; Q9, analysis. Q3–4 are NA for the assessed single-cohort before–after contrasts; exploratory comparisons between lesion subgroups do not supply an untreated control. One baseline plus several post-exercise measurements does not meet Q5.

Note: Columns 1–9 correspond to Q1–Q9 above. item-level critical appraisal; Y, yes; N, no; U, unclear; NA, not applicable. No summed quality score, arbitrary risk category or exclusion decision is assigned. Reasons specify unmet/unclear criteria; affirmative items indicate reported safeguards, not proof that all bias has been removed.

Methodological references for Tables S10b to S10k

Tool sources and versions are listed in Table S10l and use the manuscript bibliography. Checklist labels are abbreviated; the official instruments govern domain and item judgments.

Abbreviations: BDNF, brain-derived neurotrophic factor; JBI, JBI critical-appraisal instruments; NA, not applicable; N, no; Y, yes; U, unclear. Response codes retain their instrument-specific meanings. JBI: Y, yes; N, no; U, unclear; NA, not applicable.

Table S10l. Sources and versions of the methodological appraisal tools	
**Tool**	**Methodological source**
QUIPS	Hayden, J.A.; van der Windt, D.A.; Cartwright, J.L.; Côté, P.; Bombardier, C. Assessing bias in studies of prognostic factors. Ann. Intern. Med. 2013, 158, 280–286. https://doi.org/10.7326/0003-4819-158-4-201302190-00009. [24]
PROBAST+AI	Moons, K.G.M.; Damen, J.A.A.; Kaul, T.; Hooft, L.; Andaur Navarro, C.L.; Dhiman, P.; et al. PROBAST+AI: An updated quality, risk of bias, and applicability assessment tool for prediction models using regression or artificial intelligence methods. BMJ 2025, 388, e082505. https://doi.org/10.1136/bmj-2024-082505. [25]
JBI analytical cross-sectional	Barker, T.H.; Hasanoff, S.; Aromataris, E.; Stone, J.C.; Leonardi-Bee, J.; Sears, K.; Klugar, M.; Tufanaru, C.; Moola, S.; Liu, X.-L.; et al. The revised JBI critical appraisal tool for the assessment of risk of bias for analytical cross-sectional studies. JBI Evid. Synth. 2026, 24, 401–408. https://doi.org/10.11124/JBIES-24-00523. [28]
JBI quasi-experimental	Barker, T.H.; Habibi, N.; Aromataris, E.; Stone, J.C.; Leonardi-Bee, J.; Sears, K.; Hasanoff, S.; Klugar, M.; Tufanaru, C.; Moola, S.; et al. The revised JBI critical appraisal tool for the assessment of risk of bias for quasi-experimental studies. JBI Evid. Synth. 2024, 22, 378–388. https://doi.org/10.11124/JBIES-23-00268. [31]
JBI case series	Munn, Z.; Barker, T.H.; Moola, S.; Tufanaru, C.; Stern, C.; McArthur, A.; Stephenson, M.; Aromataris, E. Methodological quality of case series studies: An introduction to the JBI critical appraisal tool. JBI Evid. Synth. 2020, 18, 2127–2133. https://doi.org/10.11124/JBISRIR-D-19-00099. [30]
JBI cohort and case-control	JBI. Critical Appraisal Tools. JBI, Adelaide, Australia. Available online: https://jbi.global/critical-appraisal-tools (accessed on 5 October 2026). [29]
RoB 2	Sterne, J.A.C.; Savović, J.; Page, M.J.; Elbers, R.G.; Blencowe, N.S.; Boutron, I.; Cates, C.J.; Cheng, H.-Y.; Corbett, M.S.; Eldridge, S.M.; et al. RoB 2: A revised tool for assessing risk of bias in randomised trials. BMJ 2019, 366, l4898. https://doi.org/10.1136/bmj.l4898. [22] Crossover guidance (18 March 2021): Cochrane RoB 2 Development Group. Revised Cochrane risk-of-bias tool for randomized trials (RoB 2): Additional considerations for crossover trials, 18 March 2021. Cochrane 2021. Available online: https://www.riskofbias.info/welcome/rob-2-0-tool/rob-2-for-crossover-trials (accessed on 5 October 2026). [23]
ROBINS-I selection boundary	Risk of bias tools programme. ROBINS-I resources, including the V2 programme: https://www.riskofbias.info/welcome/robins-i-v2. No controlled nonrandomized intervention effect was identified among the 39 primary appraisal units; no ROBINS-I judgments are assigned.

Abbreviations: JBI, JBI critical-appraisal instruments; RoB 2, revised Cochrane risk-of-bias tool for randomized trials; QUIPS, Quality in Prognosis Studies; PROBAST+AI, Prediction model Risk Of Bias ASsessment Tool with updated provisions for regression and artificial intelligence methods; L, low risk; M, moderate risk; H, high risk; Y, yes; ROBINS-I, Risk Of Bias In Non-randomized Studies of Interventions.

Table S10m. Separate structured assessment of material secondary biomarker associations			
**Report and secondary analytical unit**	**Selection, completeness and measurement**	**Confounding and analysis**	**Interpretive consequence**
Allison et al. (2016) [38]. Inflammatory-marker changes versus 12-week NPQ change; exploratory backward regression, n=20.	All 20 trial participants completed follow-up. Serum analytes were assessed in triplicate and the blood examiner was blinded to assignment. NPQ was self-reported by participants aware of treatment. Biomarker and pain changes cover the same interval.	Backward selection was performed across multiple inflammatory candidates and pain components. CES-D change was considered in an additional model, but adequate adjustment for assigned treatment, baseline clinical differences and other mediator–outcome confounding was not demonstrated. No independent model evaluation or formal causal mediation analysis was reported.	A trial-embedded observational change–change association. Randomization of diet does not randomize biomarker change, and the RoB 2 treatment-effect judgment does not establish mediation or independent individual prognosis.
Agulló-Ortuño et al. (2024) [42]. Within late sequence, ΔIL-8 versus Δ10MWT from baseline to week 12; Pearson r=−0.776, p=0.003.	The complete biomarker cohort was 40 of 50 randomized participants, but functional availability varied. The pairwise denominator for this correlation was not reported. Cytokine and walking changes cover the same interval.	Bivariate within-sequence correlation without multivariable confounder control. Numerous cytokine–function/time comparisons were explored and the table selectively presents statistically significant correlations; multiplicity control for these correlations was not established. Baseline neurological status, natural recovery and treatment-period history were not separated.	An exploratory observational association with incomplete pairwise reporting. It does not establish an aquatic-therapy mechanism, an incremental prognostic marker or a biomarker that selects treatment benefit.
Todd et al. (2021) [41]. Baseline IL-1ra versus immediate residualized NPS change after one exercise bout; Pearson r=0.83, p=0.010, n=8; 90-minute association nonsignificant.	Ten enrolled: one withdrew because of a pressure sore and blood could not be obtained from one participant. IL-1ra was measured in duplicate, with reported intra-assay CV 5.91%. NPS was adapted for instantaneous pain and collected without blinded assessment.	Residualized pain change accounts for baseline pain statistically but does not control lesion phenotype, cannabis use, achieved exercise intensity or other response determinants. The baseline-factor analysis was pooled across eight participants and formed part of multiple cytokine/time-point comparisons. The report describes no untreated comparator or multiplicity-adjusted inference for this association.	A baseline-factor/acute-response association distinct from the uncontrolled before–after contrast. The finding does not establish a predictive treatment-effect interaction, sustained benefit or a validated prognostic threshold.
Cuce et al. (2019) [40]. Within the neuropathic-pain subgroup, peak ACTH-stimulated cortisol versus NRS pain, n=15; low- and standard-dose challenges.	Defined chronic SCI pain subgroup with established pain classification; standardized morning endocrine testing and assay procedures. NRS summarizes pain over the preceding week, whereas cortisol is elicited during a physiological challenge.	Unadjusted Spearman correlations. Pain phenotype, injury characteristics and other determinants were not jointly controlled. Several cortisol-response measures and pain scales were examined across two challenge doses; multiplicity protection for the correlations was not established.	A concurrent pain–adrenal-response association, distinct from between-group physiology. It neither establishes psychological-stress causation nor shows cortisol-mediated neurological recovery.

Note: These result-specific contextual assessments use S10a and supplement the formal primary units in S10b–S10k. They do not produce instrument scores or alter report counts. Concurrent severity and case–control discrimination findings within prognostic reports remain descriptive. PROBAST+AI judgments in S10e–S10f concern specified future-outcome models and do not constitute formal diagnostic-accuracy appraisal.

Abbreviations: NPQ, Neuropathic Pain Questionnaire; CES-D, Center for Epidemiologic Studies Depression Scale; IL, interleukin; 10MWT, 10-metre walk test; NPS, Neuropathic Pain Scale; CV, coefficient of variation; ACTH, adrenocorticotropic hormone; NRS, numerical rating scale; Δ, change; RoB 2, revised Cochrane risk-of-bias tool for randomized trials; PROBAST+AI, Prediction model Risk Of Bias ASsessment Tool with updated provisions for regression and artificial intelligence methods.

**Table S11. Report-specific context for bias, precision and applicability**			
**Report**	**Participation and attrition**	**Biomarker and endpoint measurement**	**Confounding and analysis/reporting**
Mechanick et al. (1997) [35]	Clinically selected metabolic-bone sample; TBI separate; Retrospective chart completeness unclear	Clinical serum measures; SEM retained; Concurrent ASIA only	Age, severity and illness may confound. Adjustment in PTH/vitamin-D analyses does not establish adequate control for qualifying cortisol/thyroid comparisons; concurrent results, no future prognosis.
Morse et al. (2008) [36]	Mobility-selected men; no statin use; One CRP outlier excluded; mobility counts conflict	Plasma hsCRP standardized; Self-reported mobility; concurrent	BMI/illness adjustment; residual confounding; No temporality; no recovery model
Kwon et al. (2010) [33]	Selected examineable acute cohort; Motor model n=20; small test split	CSF kinetics; growth factors below detection; Six-month motor outcome; baseline AIS	Severity/level influence model; Internal split/bootstrapping; no external validation
Hayakawa et al. (2012) [43]	Small acute cervical sample; Variable missing draws/follow-up windows	Below-limit pNF-H set to zero; Final AIS group differs from conversion	Initial severity not isolated; Small subgroup comparisons; no validated prognosis
Harness et al. (2014) [37]	Existing chronic ABT participants; Cortisol sequence n=12 of 15	Midday serum; fixed modality order; Motor-score correlation; no durable recovery	No control; modality/carryover effects; Null BDNF/motor tests; acute response only
Pouw et al. (2014) [44]	Two centres; selected early CSF; 16 included/23 screened; one NfH sample insufficient	Single ≤24-h CSF; Different 6-/12-month endpoints	Very small baseline-severity subgroups; Descriptive fold differences; no external validation
Takahashi et al. (2014) [60]	Compressive worsening/chronic phenotype; 14 analysed vs initial 15 wording	Preoperative CSF pNF-H; JOA at six months; null recovery relation	Disease course and surgery; Small groups; no calibrated prognosis
Ungureanu et al. (2014) [45]	15 traumatic case-series patients; Early death; serial draws variable	Lumbar CSF; freezing detail incomplete; Individual AIS trajectories	Surgery/co-interventions; Descriptive kinetics; no adjusted recovery coefficient
Ahadi et al. (2015) [17]	Overall trauma-cohort age range 16–64 years, without a separate SCI age range. Fracture controls. Eight deaths. Analyte-specific n unclear.	Serum assay values/text inconsistencies; Initial severity/early survival only	Severity and critical illness; No longitudinal functional prognosis
Kato et al. (2015) [61]	Chronic compressive myelopathy; young controls; Postoperative serum 18/26	Serum/plasma wording; detection threshold; mJOA/recovery at three months	Disease duration/surgery; Only six detectable baseline samples; recovery null
Allison et al. (2016) [38]	20 chronic SCI; randomized treatment; 20 complete; one later diet record missing	Timed serum; examiner blinded; Subjective sensory pain; unblinded participants	Stable medication; observational biomarker analysis; Multiple cytokines; exploratory model, no causal mediation
Goldstein et al. (2017) [39]	338/360 chronic participants; Excluded biomarker/covariate records	Plasma hsCRP standardized; Concurrent self-reported mobility	Adiposity/illness; five adjusted models; One model null; no longitudinal directionality
Kwon et al. (2017) [34]	Selected acute AIS A–C cohort; 48/50 clinical follow-up	CSF; growth-factor detectability limited; Six-month conversion/motor outcomes	Baseline severity/level; Internal resampling; SEs and 95% CIs reported; no external validation

**Table S11. Report-specific context for bias, precision and applicability**			
**Report**	**Participation and attrition**	**Biomarker and endpoint measurement**	**Confounding and analysis/reporting**
Singh et al. (2017) [46]	Nonoperative/refused-surgery cohort; Serial specimen denominators vary	Plasma pNF-H; Numeric AIS coding; 90-day series	Severity and treatment selection; Concurrent correlations; no adjusted prognostic model
Streijger et al. (2017) [47]	29 acute programme patients; 27 clinical follow-up	LC-MRM; below-LLOQ handling; Severity/conversion/motor change separated	Adjusted AIS/level/motor baseline; FDR removes conversion signals; internal cross-validation only
Du et al. (2018) [48]	60 selected acute traumatic SCI; Outcome-group n=21/39	Same-batch serum ELISA; Six-month AIS improvement	Baseline motor/sensory imbalance; Unadjusted ROC; no external validation
Cuce et al. (2019) [40]	22 chronic SCI; pain subgroup 15; Correlation n=15; no longitudinal attrition	Morning ACTH challenges; two doses; Concurrent pain NRS	Pain drugs/stress/phenotype; Correlations without CI; no recovery inference
Rieder et al. (2019) [49]	Isolated acute ICU SCI; Mortality; 16 paired samples of 52	48-h/day-7 serum; analyte n differs; Six-month AIS/FIM total	No adjusted marker model; Null tests; endpoint-specific n incompletely reported
Capirossi et al. (2020) [50]	11 recruited; 9 analysed rehabilitation patients; Death/contaminated CSF excluded	Multiplex 38-analyte CSF; Discharge 47–320 days; SCIM/motor	Co-injury, baseline severity, rehabilitation; High-dimensional n=9 correlations; no CI/validation
Holmström et al. (2020) [62]	12 acquired tethering patients; 15 surgical occasions, not 15 patients	Different CSF collection by comparator; AIS/EQ-5D/JOACMEQ; three-month outcome	Aetiology and surgery; Repeated cases; null clinical-score associations
Lin et al. (2021) [63]	45 SCI; genotype subgroups; Serial missingness multiply imputed	EDTA plasma multiplex; Concurrent pain/inflammatory trajectories	Severity/genotype/time; Interaction tests; not treatment mediation
Todd et al. (2021) [41]	Physically active chronic NP participants; 10 enrolled/8 analysed; subgroups of four	Fasting plasma; pre/post/90 minutes; Acute pain/affect, not training recovery	Uncontrolled; achieved exertion differs; Post-hoc correlations; printed CI column ambiguous
Itodo et al. (2022) [18]	86 rehabilitation patients; >16 permitted; Baseline neurological data missing in 14	Serum; sampling 07:00–14:00; Endocrine trajectory, not neurological change link	Adjusted age/BMI/level/completeness/drugs; Sex-specific estimates; no recovery mediation
Wichmann et al. (2022) [64]	15 acute SCI; same 2023 cohort; 11 follow-up; four conversions	Olink log2 units; CSF GDNF undetectable; AIS/motor/SCIM follow-up	Small phenotype groups; Multiplicity-adjusted GDNF signal; no threshold validation
Stukas et al. (2023) [51]	118 selected AIS A–C patients; 113 follow-up; AIS A n=74, cervical motor n=65	Paired serum/CSF Simoa; repeated specimens; Conversion/motor thresholds differ	Includes initial severity/sensory ZPP; Internally derived thresholds; programme overlap
Wichmann et al. (2023) [65]	Same 15 acute patients; 11 clinical follow-up	Simoa/MSD; serum NSE haemolysis exclusion; Initial AIS vs conversion/SCIM	Small severity strata; Range capping/imputation; later-outcome null
Yang et al. (2023) [52]	15 AIS A/B; clinical drain selection; Four discharge improvers	CSF/serum panels; selected drainage; Discharge <3 months, variable time	Durotomy/surgery; baseline severity; Many comparisons; mainly null improvement findings

Table S11. Report-specific context for bias, precision and applicability			
**Report**	**Participation and attrition**	**Biomarker and endpoint measurement**	**Confounding and analysis/reporting**
Agulló-Ortuño et al. (2024) [42]	AIS C/D; assisted standing required; 40/50 biomarkers; functional n smaller	EDTA plasma; repeated multiplex; 10MWT vs other function; no circuit test	Sequence/time/usual therapy; no washout; Some FDR tests; pairwise correlation n NR
Ding et al. (2024) [21]	Retrospective cervical/surgical selection; Exclusions and one-year selection	Blood CRP matrix/platform NR; Later AIS status, not necessarily conversion	MRI/severity adjustment; OR coding ambiguous; Development ROC; no external validation
Haddadi et al. (2024) [53]	31 selected traumatic SCI; Complete n=9/incomplete n=22	Serum and CSF assay definitions differ; Initial completeness only	Baseline severity; ROC CI NR; no longitudinal prognosis
Pu et al. (2024) [20]	All SCI plus TBI; Outcome groups 42/11/7	Serum-labelled NSE/neuritin; wording inconsistent; Global GOS horizon insufficiently specified	Brain injury cannot be separated; Apparent near-perfect performance; no validation/CI
Azad et al. (2025) [54]	50 mixed traumatic/compressive patients; Proteomic prognosis n=34	cfDNA ddPCR plus NULISA; Six-month AIS conversion	Clinical phenotype and baseline severity; Multi-analyte development AUC; CI NR
Fraussen et al. (2025) [55]	37 multicentre traumatic SCI; 34 endpoint records. Reported outcome-group totals: 33 conversion, 29 motor. Separate ROC n not stated.	Plasma Simoa/Luminex; blinded duplicates; Variable latest endpoint; motor change distinct	Baseline injury severity; Multiple predictors; no cross-validation
Morrison et al. (2025) [66]	36 acute SCI; co-injuries common; ELISA 35; RNA 36; flow 14	Plasma panels; half-LLOQ imputation; Initial motor completeness only	Injury burden/co-trauma; Different assay n; no prospective prognosis
Raheja et al. (2025) [56]	26 adults; no head injury/polytrauma; 10 deaths+7 losses; nine follow-up	Serum ELISA; analyte completeness unclear; Later AIS status; small survivor endpoint	Baseline severity/survival selection; Perfect subgroup AUC highly unstable; no validation
Seijas et al. (2025) [19]	80 paired-sample biobank participants; ≥16 permitted; possible overlap with Itodo; 65 men/15 women	Serum ELISA, 07:00–14:00; clinical missingness imputed under MAR; baseline median 42 days; subsequent SCIM-III functioning	Baseline neurological severity/function incompletely controlled; subgroup findings and transformed percentages; observed hormone associations do not establish benefit of supplementation
Vedantam et al. (2025) [67]	20 degenerative myelopathy; Missing strength/NfL/cytokine samples	Serum by processing; some values unquantifiable; Three-month pinch/function	Chronic disease and surgery; Multiple models in small cohort; no external validation
Waliullah et al. (2025) [57]	26 acute SCI; no steroids; Serial endpoint completeness unclear	Serum concentrations conflict within report; Severity at three time points	Initial completeness and management; Serial group tests; no change-regression prognosis
Belogurov et al. (2026) [68]	8 analysed tumour/surgical patients; 10 enrolled; human correlation n unclear	CSF/plasma repeated perioperative panels; Discharge Karnofsky; no human drug endpoint	High-dose corticosteroids/surgery; Rodent drug benefit separate; no human biomarker–MEP pathway

Table S11. Report-specific context for bias, precision and applicability			
**Report**	**Participation and attrition**	**Biomarker and endpoint measurement**	**Confounding and analysis/reporting**
Hei et al. (2026) [58]	71 single-centre cervical SCI; Outcome-group n=38/33	Serum according to collection and assay Methods; conflicting plasma terminology in the title and Results; Methods six months; inconsistent outcome wording	Admission AIS+IMLL+tau model; Development AUC; no external validation
Sheinberg et al. (2026) [59]	25 selected acute SCI patients; 38 quality-controlled RNA specimens; distinct 10-patient qPCR set	CSF exosome fraction; blinded duplicates; AIS improvement definition inconsistent	Initial severity and injury level; Protein models unvalidated externally; qPCR validates one miRNA only
Note: This descriptive context complements the standardized assessments in S10b–S10k; it is not a separate six-domain score or a certainty grade. The primary appraisal units are specified in those tables. Concerns qualify attribution, prognosis, precision or applicability. Randomization of treatment does not randomize biomarker exposure, and overlapping reports do not constitute independent replication.			

Abbreviations: SCI, spinal cord injury; TBI, traumatic brain injury; AIS, American Spinal Injury Association Impairment Scale; ASIA, American Spinal Injury Association; CSF, cerebrospinal fluid; CRP, C-reactive protein; hsCRP, high-sensitivity C-reactive protein; BDNF, brain-derived neurotrophic factor; GDNF, glial cell line-derived neurotrophic factor; NSE, neuron-specific enolase; NfL, neurofilament light chain; NfH, neurofilament heavy chain; pNF-H, phosphorylated neurofilament heavy chain; SEM, standard error of the mean; SE, standard error; CI, confidence interval; AUC, area under the curve; ROC, receiver operating characteristic; OR, odds ratio; BMI, body mass index; ACTH, adrenocorticotropic hormone; MRI, magnetic resonance imaging; IMLL, intramedullary lesion length; SCIM, Spinal Cord Independence Measure; FIM, Functional Independence Measure; JOA, Japanese Orthopaedic Association score; mJOA, modified Japanese Orthopaedic Association score; JOACMEQ, Japanese Orthopaedic Association Cervical Myelopathy Evaluation Questionnaire; EQ-5D, EuroQol five-dimension instrument; GOS, Glasgow Outcome Scale; NP, neuropathic pain; NRS, numerical rating scale; 10MWT, 10-metre walk test; EDTA, ethylenediaminetetraacetic acid; ELISA, enzyme-linked immunosorbent assay; LC-MRM, liquid chromatography multiple-reaction monitoring; MSD, Meso Scale Discovery; Simoa, single molecule array; LLOQ, lower limit of quantification; FDR, false discovery rate; RNA, ribonucleic acid; miRNA, microRNA; qPCR, quantitative polymerase chain reaction; ddPCR, droplet digital polymerase chain reaction; cfDNA, cell-free deoxyribonucleic acid; MEP, motor evoked potential; ZPP, zone of partial preservation; NR, not reported; ABT, activity-based training; ICU, intensive care unit; NULISA, nucleic acid-linked immunosandwich assay.

Table S12. Prescribed and achieved rehabilitation or challenge exposure

Report	Prescribed exposure	Achieved exposure, intensity, assistance and interruptions	Clinical relationship and concurrent care
Harness et al. (2014) [37]	Single 2-h bout: standing, BWSTT, WBV and FES, approximately 30 min each.	BWSTT prescribed 10×2-min intervals/1-min rests with 60% support; WBV 15×1-min intervals/rests; FES 30 min. Actual participant-specific active minutes/repetitions NR. Fixed sequence.	Acute biomarker response and motor-score association; current ABT participants; no durable recovery assessment.
Allison et al. (2016) [38]	12-week diet plus supplements, not physical training.	Mean self-reported adherence 89% (70–100%); one participant missing later records. All 20 completed clinical trial.	Pain/cytokine treatment contrasts; stable pain medication; no formal mediation.
Rieder et al. (2019) [49]	Inpatient physiotherapy twice/day; outpatient ≥6 months, 3/week.	Session minutes, repetitions, progression, achieved dose/adherence and assistance NR.	Biomarker prognosis was null; concurrent care not quantified as an explanatory exposure.
Capirossi et al. (2020) [50]	Inpatient rehabilitation.	Session frequency/minutes, repetitions, progression, adherence and assistance NR.	Early CSF correlated with discharge motor/SCIM; no dose-response model.
Todd et al. (2021) [41]	Single 30-min arm-crank bout at 60% peak power; reduce to 50% when exertion threshold exceeded.	RPE monitored; paraplegia subgroup exceeded intended intensity. Hand/positioning assistance as needed. Two exclusions before final n=8; no longitudinal adherence/progression.	Immediate/90-min pain and affect; no control or training course.
Itodo et al. (2022) [18]	Initial inpatient rehabilitation.	Median rehabilitation duration 5.6 months; active treatment dose NR.	Endocrine trajectory, not endocrine-change–neurological-recovery analysis; medication covariates considered.
Agulló-Ortuño et al. (2024) [42]	18 sessions, 3/week, 40 min/session for 6 weeks; early versus late aquatic sequence.	RPE targets/monitoring reported. Actual individual completed minutes, repetitions and adherence NR. Assisted standing required. Withdrawals/discharge/infection and inadequate samples contributed to exclusion of ten biomarker cases.	Usual land therapy throughout 12 weeks; exploratory IL-8–10MWT change relationship; no washout or formal mediation.
Seijas et al. (2025) [19]	Initial inpatient rehabilitation in four Swiss centres	Median follow-up 167 days (IQR 128–224); active session minutes, repetitions, assistance, achieved dose and adherence not quantified	Serum androgen–SCIM-III associations under usual rehabilitation; no randomized hormone intervention, dose mediation or biomarker×treatment interaction
Belogurov et al. (2026) [68]	Rehabilitation from postoperative day 2 to discharge.	Actual active dose/adherence NR.	Perioperative steroids: methylprednisolone 1 g and dexamethasone 8 mg over four days; human cytokine–discharge status, not clinical antagonist efficacy.

Table S12. Prescribed and achieved rehabilitation or challenge exposure			
**Report**	**Prescribed exposure**	**Achieved exposure, intensity, assistance and interruptions**	**Clinical relationship and concurrent care**
Reporting counts across the corpus	Three physical protocols provide session duration: one course (aquatic) and two single bouts (Harness/Todd). Rieder additionally provides session frequency.	No physical training-course report provides individual completed active minutes or achieved repetitions. Harness/Todd provide acute intensity/support detail; neither supplies longitudinal progression/adherence. One report provides dietary adherence.	No report tests achieved-dose mediation or a biomarker×randomized-treatment interaction. Withdrawal reporting is not a quantified treatment-interruption dose.
Note: The other 32 reports do not quantify rehabilitation exposure as a reviewed intervention/challenge. Clinical co-interventions may still have occurred. Prescribed intervals and physiological test doses are not equated with completed active rehabilitation. ACTH tests in Cuce are pharmacological challenges, not rehabilitation.			

Abbreviations: CSF, cerebrospinal fluid; IL, interleukin; IL-8, interleukin-8; ACTH, adrenocorticotropic hormone; SCIM, Spinal Cord Independence Measure; 10MWT, 10-metre walk test; BWSTT, body-weight-supported treadmill training; FES, functional electrical stimulation; WBV, whole-body vibration; RPE, rating of perceived exertion; NR, not reported; ABT, activity-based training.

Table S13. Contribution of post hoc eligibility exceptions to the descriptive synthesis		
**Report and exception**	**Contribution retained in the main map**	**Interpretation when omitted**
Ahadi et al. (2015) [17]. Overall trauma-cohort age range 16–64 years. SCI-specific age range and adult-only estimates unavailable.	Serum GFAP, pNF-H and NSE relationships with initial AIS severity and early survival.	One small SCI severity series with unresolved age composition is omitted. Other neural injury-marker reports retain concurrent severity and subsequent neurological analyses. The omitted findings do not establish adult-specific effects.
Itodo et al. (2022) [18]: SwiSCI recruitment permitted age >16 years; adult-only estimates unavailable.	Serum DHEA/DHEAS and broader androgen trajectories during initial rehabilitation; baseline injury-completeness relationships.	The direct rehabilitation-period adrenal-androgen trajectory analysis is removed. Other reports retain basal or stimulated endocrine measurements but do not replace this trajectory evidence.
Seijas et al. (2025) [19]: SwiSCI recruitment permitted age ≥16 years; adult-only estimates unavailable.	Baseline DHEA/DHEAS and Rasch-transformed SCIM-III functioning; subgroup/time-dependent findings and null fully adjusted discharge estimates.	The adrenal-androgen–independence analysis is removed. Other reports assess FIM or SCIM-III with neural/inflammatory markers; these do not establish an alternative endocrine–function relationship.
Pu et al. (2024) [20]: all 60 participants had concomitant SCI and TBI.	NSE-containing analyses against Glasgow Outcome Scale prognosis in combined neurotrauma; the source describes the endpoint as TBI prognosis.	Combined-neurotrauma prognosis is removed. Spinal neurological severity, status and change remain represented elsewhere; Pu does not independently support spinal recovery in either synthesis.
Ding et al. (2024) [21]: CRP blood matrix not identified as serum or plasma.	CRP, MRI severity measures and one-year AIS A–C versus D–E status; CRP-plus-BASIC model.	The matrix-uncertain CRP prognostic estimates and CRP–BASIC model are removed. Inflammation–mobility/pain and other multimodal biomarker analyses remain, without serving as validation of this model.

Note: The main evidence map retains all 41 reports. The descriptive sensitivity comparison was undertaken post hoc after the initial synthesis. The descriptive sensitivity comparison jointly sets aside the five listed reports, leaving 36 reports. It examines whether the narrative interpretations remain supported; no participant-level analysis, pooling or recalculation of published estimates was performed. The comparison subset does not establish that all remaining reports represent independent cohorts or uniformly verified adult-only samples.

The age exception in Itodo and Seijas concerns permissive recruitment criteria and unavailable adult-only estimates; actual inclusion of participants aged <18 years is not established. Ahadi reports the 16–64-year range for the 35-person trauma cohort, including nine fracture controls; the exact number or SCI status of participants aged <18 is not reported. Itodo and Seijas may include overlapping SwiSCI participants and are complementary reports rather than independent replications. No neurotrophic-domain report is omitted by this comparison. Abbreviations: AIS, American Spinal Injury Association Impairment Scale; BASIC, Brain and Spinal Injury Center; CRP, C-reactive protein; DHEA, dehydroepiandrosterone; DHEAS, dehydroepiandrosterone sulfate; FIM, Functional Independence Measure; GFAP, glial fibrillary acidic protein; MRI, magnetic resonance imaging; NSE, neuron-specific enolase; pNF-H, phosphorylated neurofilament heavy chain; SCI, spinal cord injury; SCIM-III, Spinal Cord Independence Measure version III; SwiSCI, Swiss Spinal Cord Injury Cohort Study; TBI, traumatic brain injury.

Abbreviations: PRISMA-ScR, Preferred Reporting Items for Systematic Reviews and Meta-Analyses extension for Scoping Reviews; PRISMA, Preferred Reporting Items for Systematic Reviews and Meta-Analyses.

Fluid biomarker measurement coverage

41 reports

Domains may overlap within a report.

Report	E	I	T	N
Mechanick 1997	M	—	—	—
Morse 2008	—	M	—	—
Kwon 2010	—	M	A	M
Hayakawa 2012	—	—	—	M
Harness 2014	M	—	M	—
Pouw 2014	—	—	—	M
Takahashi 2014	—	—	—	M
Ungureanu 2014	—	—	—	M
Ahadi 2015	—	—	—	M
Kato 2015	—	—	—	M
Allison 2016	—	M	—	—
Goldstein 2017	—	M	—	—
Kwon 2017	—	M	A	M
Singh 2017	—	—	—	M
Streijger 2017	—	—	—	M
Du 2018	—	—	—	M
Cuce 2019	M	—	—	—
Rieder 2019	—	M	M	M
Capirossi 2020	—	M	M	M
Holmström 2020	—	—	—	M
Lin 2021	—	M	—	—

Report	E	I	T	N
Todd 2021	—	M	—	—
Itodo 2022	M	—	—	—
Wichmann 2022	—	M	M	—
Stukas 2023	—	—	—	M
Wichmann 2023	—	M	—	M
Yang 2023	—	M	—	M
Agulló-Ortuño 2024	—	M	—	—
Ding 2024	—	M	—	—
Haddadi 2024	—	—	—	M
Pu 2024	—	—	—	M
Azad 2025	—	M	—	M
Fraussen 2025	—	—	—	M
Morrison 2025	—	M	—	—
Raheja 2025	—	—	—	M
Seijas 2025	M	—	—	—
Vedantam 2025	—	M	M	M
Waliullah 2025	—	—	—	M
Belogurov 2026	—	M	—	—
Hei 2026	—	—	—	M
Sheinberg 2026	—	M	M	M

M Quantitative measurement

— Domain not assessed

A Assayed without reliable quantitative findings

Measurement does not establish a positive clinical association.

E Neuroendocrine (5 reports)

I Inflammatory (19 reports)

T Neurotrophic (8 reports: 6 M, 2 A)

N Neural and glial injury (26 reports)

Figure S1. Biomarker domains assessed across the 41 included reports. Each row represents one report, ordered chronologically and alphabetically by first author within each publication year. The left block contains 21 reports and the right block contains 20. The sequence runs from top to bottom in the left block and then continues in the right block. E denotes neuroendocrine biomarkers, I inflammatory biomarkers, T neurotrophic factors, and N neural and glial injury biomarkers. M indicates that a quantitative measurement of an eligible biomarker was reported in the domain. A indicates that an eligible analyte was assayed without reliable quantitative findings. The two A cells identify the neurotrophic domain in Kwon et al. 2010 and Kwon et al. 2017. The em dash (—) indicates that the domain was not assessed and does not denote a null clinical association. A report with a quantified biomarker and a null clinical finding remains coded M. The non-exclusive counts are five neuroendocrine, 19 inflammatory, and 26 neural and glial injury reports, together with eight reports assessing neurotrophic analytes, of which six are coded M and two A. Domain membership is non-exclusive. Quantitative measurement does not establish a positive clinical association, prognostic validity, or neuroplasticity. Reports may describe overlapping cohorts. Assessment of multiple domains in one report does not establish that relationships between those domains were tested. Neuroendocrine measurements represent distinct physiological constructs and should not be interpreted collectively as measures of hypothalamic–pituitary–adrenal activity or psychological stress. Clinical relationships and the integration with imaging or residual neurological function actually examined are described in Table S8b. Established or possible cohort dependence is documented in Table S6c.
